# Supplementary material for: ‘It is what was handed over to us as our heritage and must not be taken away just like that’: Traditional birth attendants’ attitudes towards the elimination of intergenerational female genital mutilation/cutting in Osun State, Nigeria
Source: PLOS Glob Public Health. 2024 May 20;4(5):e0001585. doi: 10.1371/journal.pgph.0001585 (PMC11104658; doi:10.1371/journal.pgph.0001585)
Supplement: S1 File — (DOCX) [file pgph.0001585.s001.docx]

TRANSCRIPTS OF THE INTERVIEWS OF THE PARTICIPANTS

TBA INTERVIEW 1 ENGLISH P1 A45 TE- participant 1 Age 45 Tertiary Education

Interviewer: Good afternoon, sir

Respondent: Good afternoon ma

Interviewer: My name is Mrs. Omolara Fafowora. I am a student conducting a study on female circumcision. I am here to meet you as a key informant and a community leader.

Respondent: okay

Interviewer: I thank you for honouring my request to visit you.

Respondent: You are welcome.

Interviewer: Can you please tell me how old you are?

Respondent: I am up to 45 years old.

Interviewer: what level of education did you attain?

Respondent: I was educated to higher level of learning (Higher National Diploma).

Interviewer: tertiary….

Respondent: Yes, tertiary education.

Interviewer: Since when have you been living in this community?

Respondent: this is where I have been living right from the onset. I was born and bred.

Interviewer: This is where you were born?

Respondent: Yes, from childhood.

Interviewer: so how did you turn to be a Traditional Birth Attendant (TBA)?

Respondent: It is like a child born into a family that traditionally specialises in drum beating. specializes in drum beating...

Interviewer: Hmm

Respondent: the child will know it, as they go for drumming outing, he will be following them

Interviewer: like inheritance….

Respondent: we matured, we were born into it, we didn’t learn it, our forefathers also inherited it, they migrated from time immemorial from Oyo

Interviewer: We can say that it runs in the family

Interviewer: that is when you have…….

Respondent: I have been following my father.

Interviewer: okay

Respondent: I was born into it, I didn’t learn it, my forefathers also inherited it.

Interviewer: hmm

Respondent: Yes, ma

Interviewer: Can you please tell me when you really started this work of TBA?

Respondent: I started around age 15. Before that age, I only follow him around with other children.

Interviewer: very interesting

Respondent: The money realised would be handed over to him. This was on until he asked me to stop giving him the money.

Interviewer: okay

Interviewer: with what you have told me now, can you tell me how long you have been practising as a TBA?

Respondent: If I am not mistaken, it will be more than 25 years that I have been performing the procedure on my own.

Interviewer: Really

Respondent: Yes, it has been more than 25 years

Interviewer: On the average, how many women do you attend to in a month?

Respondent: initially, this place used to be full of women but now I barely attend to about 8 women in a month.

Interviewer: You mean civilization has impacted the number of people that patronise you?

Respondent: Yes. The figure varies though.

Interviewer: Thank you sir. I want to know if this is the only source of income you have.

Respondent: No, you can see for yourself.

Interviewer: with what you have been telling me since sir, you are affirming that being a TBA is transferred from one generation to another.

Respondent: Yes.

Interviewer: alright, sir. I asked if it is only circumcision that is your source of income?

Respondent: Like I have said earlier, I have other work that I do as you have seen yourself.

Interviewer: okay

Respondent: For instance, my father also learned carpentry work and was into business in his early life.

Interviewer: Thank you for the information so far. Like I told you, I am here to meet you as the community leader. Sir, I wish to know if you have participated in any government effort to stop the female genital mutilation/cutting in Osun state?

Respondent: I remember some time ago, a group of people from the government came that we should not perform FGMC again. The elders among us informed the government officials that if the law says it should not be done again, it is alright. A seminar was also organised then Mapo hall in Ibadan. In that seminar, we were educated on FGMC. The trainers brought some dolls to show us how the female body is.

Interviewer: Like an enlightenment programme

Respondent: Yes. There was an interactive session where we were allowed to express ourselves. The TBAs said that the government is only looking at it from their perspective and not reasoning with the TBAs. So, with that I can say that I have participated in government efforts before.

Interviewer: so what was the outcome of the workshop from your side?

Respondent: ahhhhhh, it doesn’t have any impact the side of the TBAs at all.

Interviewer: hmm

Respondent: civilisation of some few individuals may be the reason why the government is kicking against it. FGMC may also be seen as ritual procedure because of civilisation.

Interviewer: so you mean civilisation is a major facilitator in eliminating female circumcision

Respondent: that may be the reason why it was reduced a bit

Interviewer: ok

Respondent: The people that have the understanding that all the things the government is saying are not really like that do bring their children for circumcision, FGMC inclusive.

Interviewer: hmm

Respondent: I do not believe that it affects the girls adversely. We do have sisters who have undergone FGMC, and nothing was wrong with them. They are okay in everything they do

Interviewer: hmm

Respondent: There is none of them that we heard any history of bad consequences of FGMC such as preventing her from giving birth. It is the other way round that if a woman is not circumcised, it will affect the children they give birth to, leading to still birth

Interviewer: Alright

Respondent: so, that in the ancient times, those that have still birth were usually asked to go for re-circumcision. They may say they should re-circumcise them, because they believed then that, as they give birth, the head of the baby has touched the clitoris.

Interviewer: Is that so?

Respondent: Yes, the government said they did the research, but our fathers also did research, in traditional way. In fact, there is a way a woman is positioned that the head of the baby will not touch the clitoris but only few people do it successfully.

Interviewer: hmm

Respondent: they did the research that if the clitoris that was not circumcised has grown,………are you listening?

Interviewer: Yes sir. I am with you sir.

Respondent: that the new baby that is coming through the birth canal, as the new baby’s head touches the uncircumcised clitoris that had grown, the baby may die or not be able to have children…

Interviewer: new baby head touches the clitoris; the baby may die or not be able to have children…

Respondent: Yes. If a woman is not circumcised, during delivery if the baby’s head touches the clitoris, he/she may die or not be able to have children

Interviewer: hmm

Respondent: As a result of these, it is necessary to circumcise female children

Interviewer: thank you

Respondent: yes ma

Interviewer: thank you very much, but as a person that has been involved in female genital mutilation/cutting for a long time

Respondent: yes

Interviewer: You said earlier that there are different ways of performing FGMC. Can you please explain further?

Respondent: The type that we perform, aaaahhhh,

Interviewer: Yes sir.

Respondent: You know it is like the operation that the white do, it depends on how good individual is.

Interviewer: differs

Respondent: it differs from person to person,

Interviewer: Alright

Respondent: The clitoris can be cut deep or cut slightly such that when it is healed, you will hardly know if something happened there. That is the reason it is good for FGMC to be done by the people that have been doing it very well.

There are basically there three ways of cutting the female genitals. The lower part of the female genitals is what will be cut and the upper part of it will not be cut. Another method is to cut the upper part and the lower part. The last method is cutting the upper part only. There is none among the three methods that has shortcomings.

Interviewer: If I get you right there are three types of female circumcision

Respondent: the blade for circumcising male children is different from the one used for female circumcision which is round in shape.

Interviewer: thank you, in your own opinion, why do you think it is important have this traditional circumcision should be stopped?

Respondent: Traditionallly, it is very important to perform FGMC on girls as our culture dictates. Girls do not die from the circumcision performed Yoruba adage that says, children don’t die in the circumciser’s hand. ( children do not die when true TBAs perform circumcision), instead they die from other diseases please)

Interviewer: children don’t die in circumciser’s hand?

Respondent: if there is no reason, a female doesn’t bear “Kumolu”

Interviewer: okay

Respondent: Traditional circumciser don’t circumcise a child and the child dies

What usually happens is that if the female child is scared, the child may excrete, or urinate, that is all, and they won’t stop it, they will use cloth to cover the excreta.

Interviewer: hmm

Respondent: I feel, with my civilization and my little education, there is no danger in circumcising a girl.

Interviewer: You mean education and civilisation does not affect FGMC

Respondent: I will say NO. You are not killing a female child by circumcising her, rather she is being protected from the dangers attached to not doing it such as promiscuity, infections, excessive sexual desire etc.

Interviewer: Alright sir.

Respondent: The Quran, (Muslim Holy Book), supports the circumcision of both male and female children. It stated that a little out of the female clitoris should be removed as it is seen as dirt,

Interviewer: Thank you sir. You keep saying that FGMC poses no danger to the female child. Tell me the benefits of FGMC.

Respondent: I say it again that there is no danger at all associated with circumcising a female child. The benefits are enormous, and hydra headed.

A girl that is not circumcised will be sexually sensitive to a male excessively (nymphomania). But when the circumcision is done, there will not be such excessive sensitivity to the opposite sex. If ordinary cloth of her body just touched her in the sensitive part of her body because her genital was not cut, she will be sexually aroused. Such girls are never satisfied with one man having sexual intercourse with them.

Interviewer: hmm

Respondent: A girl that is circumcised will not be jumping from one man to another. Her sexual arousal is controlled wherever she finds herself.

Interviewer: hmm

Respondent: In the olden days, majority of our mothers were not promiscuous, it is only their husband that they are married to that they stick to. But presently that we are campaigning against FGMC, the body will be itching her all the time, once she sees a man immediately her body will respond to the man such that a man will not satisfy her. That is why you see a lot of immoral acts everywhere now.

Interviewer: thank you

Respondent: maybe you still have questions.

Interviewer: Yes sir. Another question that is related…..

Respondent: okay,

Interviewer: Family is seen as unit within a community. As a community leader, what do you think the family of circumcised female children stands to benefit?

Respondent: By circumcising their children?

Interviewer: yes.

Respondent that the: ahhhh, one of the benefits is that the girl child will not disgrace the family. A girl child that is not circumcised will have her body excessively responding sexually to man. There is the tendency that she will bring shame to the family when she is continues to be sexually involved with different men.

On the other hand, a girl child that is circumcised will not be promiscuous or have the tendency of promiscuity. Therefore, will not bring shame to her family.

Interviewer: what about the community the girl child came from? Do they also have any benefit they can derive?

Respondent: that they can derive?

Interviewer: Yes sir.

Respondent: aaaahhh, well, the community too will be a better place without transmission of any sexually transmitted infections such as HIV/AIDS and Hepatitis and other associated vices.

Interviewer: that is true, thank you. Based on you experience and all you have said to me sir,

Respondent: yes ma.

Interviewer: what will be your view about eliminating this tradition?

Respondent: Female circumcision?

Interviewer: Yes. Based on your experience, what is your view about stopping this tradition?

Respondent: ahhhhh, as a person, I don’t agree to stopping girl child circumcision. I have said it earlier that based on the level of education I have and I also came from that is practicing FGMC, I do not see it being stopped. I did it for my girls and there is no problem associated with it, I don’t think it should be stopped. It will be a great disadvantage if agree.

Interviewer: hmmmmm

Respondent: If the government insist, it is alright. There is nothing like it does not allow people to enjoy sex. It is all lies.

Interviewer: hmm

Respondent: Transmission of infection is another issue that has been raised. Since the ancient times there has not been anything like infection. They use snail, black soap and osun. With these 3 things combined together, there is nothing like sickness or transmission of infection.

Interviewer: How is that possible?

respondent: there isn’t, snail is anti-dote. it is antidote, the water that drips from its cracked base is put on the circumcised clitoris with black soap, there is nothing like infection.

Interviewer: hmmmm

Respondent: I am not in support of stopping FGMC. Not that because I am a TBA family. Not at all, even if the TBAs (“olooola’s”) are stopped from doing it and it is done in the hospital, I can go and do it in the hospital. it is not compulsory it is done in the traditional way.

Interviewer: hmm

Respondent: the government decreed law that whoever is involved in FGMC will be arrested does not stop us from doing it. There is no justification to stop it. No girl child that has any problem through the circumcision in the traditional way.

Interviewer: hmm

Respondent: It is education and civilisation that is making people to have the understanding that there is HIV/AIDS or Hepatitis infection. Nothing of such before.

Interviewer: hmm

Respondent: So, I am not in support of stopping FGMC. If the government succeeds in stopping it, the negative effects will be enormous.

Interviewer: Can you please explain further?

Respondent: The disadvantages of governments’ effort to stopping FGMC will be enormous. See what is happening nowadays in our communities. You have to be extra careful and be very good as a parent to raise your female children well. some may be promiscuous from secondary school or primary school. This is because if we no longer allowed to circumcise a female child, their clitoris will continue to grow. Immediately a male touches her. She will instantly be sexually excited. No one will be able to control them.

Interviewer: hmm

Respondent: so am not in support of them stopping it. Especially doing it in the traditional way because I have not seen any adverse effect of it at all.

Interviewer: I want to infer that you confirmed that FGMC is still being done in the traditional way despite the government’s prohibition law.

Respondent: Yes.

Interviewer: Thank you sir. Can you tell me your view about the strategies the government has been using to stop FGMC?

Respondent: I have nothing to say about it than all they are doing is out of civilisation and hatred. What has been established cannot perish. It is just like asking us to abandon our language because of civilisation. There is no where we meet that we can do without speaking our language. Girl child circumcision cannot perish.

Interviewer: That is true

Respondent: it can’t perish

Interviewer: hmm

Respondent: if the government asks us not to do female circumcision again, we will agree but the people in the community will still be bringing their female children for circumcision.

Interviewer: Do you mean that female circumcision cannot stop?

Respondent: Yes, because as the community members are bringing their female children, will be circumcising them.

Interviewer: Really?

Respondent: I will say yes because I have the understanding. The type of understanding that I have, it is not everybody that can have it outside.

Interviewer: hmm

Respondent: A friend of mine is now more convinced. He noticed that Faisa, one of his daughters sometimes uses hand to scratch her private part. He brought her for circumcision, and it was done. Since that time there is no complain whatsoever.

Interviewer: You said the daughter was scratching her private part. How old is the girl sir?

Respondent: Yes. The girl was circumcised, and she is no longer complaining of itching. Nothing is wrong about circumcising a girl child.

Interviewer: How old is the girl?

Respondent: She is about six or seven years.

Respondent: With all the prohibition laws, people still do it in Osun state in the traditional way.

Interviewer: Why is it so sir?

Respondent: Apart from it being a traditional form of service, daily earning is still made out of it. Though majority of the TBA have other work that they do, it is not enough, and the government is not doing anything about it.

Interviewer: hmm

Respondent: what does government want to provide for them to eat? It can’t be enough, it can’t be sufficient, so it can’t stop

Interviewer: hmm

Respondent: Besides, girl circumcision is inherited. The lineage will be protected.

Interviewer: Sir, it is only the girl circumcision that the government is prohibiting. You are still allowed to do that of the boy.

Respondent: I am talking about the traditional heritage here. It can’t stop, it can’t stop, it may be reduced. The law, civilisation coupled with ignorance is stopping people, but it cannot be stopped.

Interviewer: hmm

Respondent: why did you say I shouldn’t do it again, the ones I have done before, what problem do they have? hmmm they are married, is there any issue that is associated with circumcision?

The argument of the government that says a girl should not be circumcised is not valid. Some points were mentioned but those points do not hold ground all.

Interviewer: Can you please tell me those points the government is putting up?

Respondent: I have mentioned them before. The government said FGMC spreads infection such as HIV/AIDS, Hepatitis B and others. I think they also say there is also difficulty when the woman is in labor delivering babies. If all these things have been happening ever since we have been doing it, and our mothers were doing it, I am sure you too would have been affected. Perhaps you would have died at birth. P1 A45 KI

Respondent: the law they made that, don’t do this, don’t do that, cannot hold. Some people come from Lagos especially during the festive period to circumcise their daughters in the traditional way.

Interviewer: Really?

Respondent: Yes. They come to circumcise their daughters at home in the traditional way.

Interviewer: hmm

Respondent: if they go to Lagos, to Abuja, or other places, once it is festive time, they bring their children home for traditional circumcision. Especially the female ones to prevent complications.

Interviewer: hmm

Respondent: ……and they won’t go to you, or doctor or even tell the government anything that we have gone to circumcise in Oloola’s (traditional circumcisers) place, when they do not have problem with it.

Interviewer: hmm

Respondent: Has there been any child’s death in Oloola’s (traditional circumciser) place? No child’s death now, which child has HIV from Oloola’s circumcision (traditional Circumcision)? No.

Respondent: there is a tribe called the Ijebus (a part of Yoruba)

Interviewer: hmm

Respondent: This group of people puts alum on the clitoris of the baby preventing it from growing.

Respondent: that genital will not grow at all.

Interviewer: Is that an alternative to circumcision?

Respondent: Yes. The TBAs were not chanced to go there to circumcise because of war at that time. The Ijebu people then devised that means to prevent the clitoris from growing.

Interviewer: What you are telling me is that they know the importance of female circumcision?

Respondent: Exactly. So, what are we saying?

Interviewer: hmm thanks so much sir

Respondent: yes, ma

Interviewer: thank you sir, we are very grateful

Respondent: yes, ma

Interviewer: but I don’t know if you have some other things that you want to add to what had already been said?

Respondent: I only want to repeat that it is civilisation that is making us to see that this is good, and this is bad. There is no negative effect that circumcision brings. There are ways some illnesses were being managed in the olden days without the performing surgeries on the person. But nowadays because of civilisation, the doctor will immediately open up.

Interviewer: People say that the instruments being used are not sterilised and the environment where the circumcision is done is not clean at all. What do you want to say to that? how are they doing this thing; it is too local.

Respondent: If those are the reasons why the government want to eradicate it, they should teach us the modern ways of doing things.

Interviewer: alright

Respondent: make modern blades for us, train us modern ways of sterilisation.

Interviewer: okay

Respondent: government can produce that for the benefit of the community. The government is for every citizen, it is part of government’s responsibilities. At least if they see any danger from a place, and they should quickly work to stop it.

Interviewer: Sir, that is what the government is trying to do.

Respondent: No, they should not stop it. whatever the adjustment that is necessary they should do. May be in terms of the blade training on sterilisation and waste disposal etc.

The government is not supposed to stop it, because I do not see any shortcoming it at all, not because I am involved. Female circumcision should continue.

Interviewer: hmm

Respondent: It should not be eradicated

Interviewer: thank you sir

Respondent: you are welcome

Interviewer: I really appreciate, if I have any reason to come back to verify some things, I hope you will still answer

Respondent: I will, no problem

Interviewer: thank you very much sir.

Respondent: thank you

TBA INTERVIEW MAMA ENGLISH 4 (TBA 6) Review P2 A70 SF

Interviewer: good afternoon ma

Respondent: Good afternoon. I have been told that you are coming. I was also informed about your intentions. There is no problem. I will attend to you.

Interviewer: thank you ma.

Interviewer: may I know your age ma?

Respondent: Huh!

Interviewer: How old are you ma?

Respondent: Ah!

Interviewer: (Smiles)

Respondent: About 70 years old

Interviewer: Did you attend any former school?

Respondent: Me?

Interviewer: Yes ma

Respondent: Ah! I didn’t attend any school. I remember when they established schools, during the era Awolowo, I attended up to primary one, but I didn’t reach two.

Interviewer: Thank you ma. Alhaja, we don’t count somebody else’s children for them. How many children did God give you?

Respondent: That God gave me?

Interviewer: Yes ma

Respondent: They are up to seven

Interviewer: How many women are there?

Respondent: Women, there many women among them

Interviewer: They are all women?

Respondent: No. Only four of them are women

Interviewer: Did you circumcise them?

Respondent: Ah! In those days. They must be circumcised. And their girl children were circumcised too.

Interviewer: That is interesting ma. Please tell me more about how they are doing with regards to the circumcision.

Respondent: Nothing is wrong with my girls and their daughters. They are perfectly doing well and not suffering from all the lies they are mentioning on the radio.

Interviewer: Thank you ma. Is TBA the only source of income for you?

Respondent: You mean for TBAs?

Interviewer: Yes ma

Respondent: Ah! If not the money in the Cocoa that is there. How much do they give us? 2000, 1000. But you know, it is our heritage which must be promoted. It is not that it really fetches more money for us. Do you understand me? It is not what the TBAs are eating from. But what was handed over to us must not be taken away just like that! The work doesn’t feed us.

Interviewer: if I get you right ma, you mean you are also a farmer

Respondent: Yes

Interviewer: (Laughs)

Respondent: Our parents didn’t release us then for business.

Interviewer: Hmm

Respondent: We were engaged in farming. They didn’t release us for business

Interviewer: Is this the place you’ve been living since you started working?

Respondent: You mean where I was born?

Interviewer: Yes ma

Respondent: I was born in this town. At a point I travelled but I came back. So, I will say that I stay here.

Interviewer: Thank you ma. As a community leader, I wish to know if you have ever participated in any of the government efforts to stop FGM/C in Osun state.

Respondent: Very well my child. During one of the seminars that we attended, the TBAs defended the girl child circumcision and emphasis was made that it is should not be stopped as it is our traditional heritage which must not be sold out.

Interviewer: For clarity sake ma. Can you tell me your view about stopping FGMC?

Respondent: like I mentioned earlier on, girl child circumcision is our traditional heritage. Let the government continue to campaign against it. In my own view, I don’t see it stopping.

Respondent: There are set of people that are calling themselves TBAs, they are not at all. It is the circumcision that they do that doesn’t allow a woman to give birth, it is the circumcision that they do that doesn’t allow woman to enjoy her husband. They just say everything in different ways. People bring their child to us for circumcision, we don’t advertise circumcision to people, TBAs doesn’t advertise themselves, people bring their child to them for circumcision

Interviewer: Hmm

Respondent: People bring circumcision to us just like you came in.

Interviewer: Ma, do we say that you were more like apprentice to be able to do circumcision?

Respondent: We don't learn it anywhere; we grew up to meet it. We don’t learn it, as soon as they give birth us, our father will give the circumcision knife to us till we grow old, whoever that will learn must have been moving with their children. We are not like doctors that went to learn surgery. We didn’t learn surgery. We just got to know how to do it because it is our inheritance. If the government knows that TBAs is useful and meaningful, among them that are carrying rumours all around. Who among them that was not circumcised? There is a prince from Ogbomosho among them, for you to know. It’s just that there is no office, my name is heard all over Nigeria.

Interviewer: Hmm

Respondent: For your information, TBAs are in Nigeria and other foreign countries. The people that are campaigning against FGMC have been to Alaafin’s Palace of Oyo, Alaafin (traditional ruler) has argued it with them, It is a cultural and traditional issue that we are protecting.

Interviewer: Why did you say it is a traditional and cultural issue?

Respondent: They bring themselves to us. And any kings of the earth gathered together, that doesn’t have TBAs in his palace is not yet king.

Respondent: let me tell you an instance. One TBAs circumcised for someone in America, and they arrested him.

Interviewer: Go on ma.

Respondent: Luckily the Alaafin of Oyo (traditional Ruler) was in America for a visit. He had to intervene. They ask him that how could the thing be done like that? And he said it was true. It is the culture of anyone that hails from Yoruba land. The traditional ruler then pulled off his clothes and asked them to look at him that that’s how it was done for him. He told them he didn’t die because the circumcision was done by the TBAs. But if whosoever that is not TBAs try to do it, the baby die in his/her hands.

Interviewer: The baby will die?

Respondent: The baby will die in his/her hands. We are registered TBAs with the government. We even monitor the activities of our members. If you are claiming to be one and you are not, you will be sanctioned.

Interviewer: very interesting ma.

Respondent: When we were at Abuja, the government representatives and the whites were there also. They made presentations that we circumcised using knife, scissors, blades, etc. The TBAs were patiently listening. When they were done with the presentations, the leaders or our team responded that we do not use such instruments and that we don’t do circumcision the way others are doing it. We have our own circumcision knife, if we are not the one holding it, it will be difficult for whoever uses it. After he finished speaking, he was applauded.

Interviewer: You said that the way others do circumcision is not how you do yours. Please tell me how you do your circumcision, especially the female circumcision.

Respondent: with me, there is no way I will not explain the two. Just be patient with me. There is just a little something on the clitoris that we hold. You must not cut your fingernails because that is what is used to hold the clitoris. We don’t circumcise in the afternoon. Latest, it is six o'clock in the morning.

Respondent: It is those who circumcise with blade that cuts the entire libido away.

Interviewer: Hmm

Respondent: It's not that hard. What we usually hold with finger is a very small part of the clitoris like this.

Interviewer: Hmm

Respondent: It will be drawn up and cut you off just like this. That’s all.

Interviewer: Hmm

Respondent: Once she urinated like this. That’s all.

Interviewer: Thank you ma.

Respondent: For a male, there are three set of flesh on his penis. There are three set flesh. First one here, when they get to the second one, the third one will be lifted up like this and they will cut it

Interviewer: Hmm

Respondent: They have outsourced all Nigerian properties and benefits. Can you find any statue at home any longer? In those days if you delivered a set of twins. If one dies, or both die, they will bath it. My grandparents, and his siblings will all sit at the corridor like this and put “osun” on its body. Those statues were at the long run taken away by the whites. They were taken away by the whites.

Interviewer: exactly ma

Respondent: That’s the example of what’s going on in the world. Assuming the corona virus started here, all our wealthy people will have left the country and ran to the foreign countries. They would have left the poor in there to live with it. Fraudsters are now in the world, and we blacks are foolish.

No matter how long, the judgment of God is coming, but now he will still be watching.

Interviewer: May God help us.

Respondent: The fake TBAs are abusing things. They are the people you are supposed to arrest. Are they not the ones that are letting us stop the work? The female genital mutilation that you stop us from doing is known, were your great grandmothers not circumcised? Were you not born?

Interviewer: I was perfectly born

Respondent: And what our forefather uses still exist. They don’t go to the doctor. But those who is not their job, who are circumcising with blade, when they do it and it goes wrong, they will now take it to doctors, the doctors will think it is from TBAs. That one is not from TBAs. It is unfortunate that we haven’t taken it so seriously. Their cassette, if you watch it, you will be afraid of God.

Interviewer: Thank you ma. How do you sterilise and keep your instruments?

Respondent: Though we do use the same instrument for the circumcision. Now that AIDS is rampant in the town, then we now have many of the instruments. If we use one for a child, we’ll use the other one for another child. We’ll now put it in a neutralizing chemical.

Interviewer: disinfectant?

Respondent: Yes. It means that it will not be used again that day.

Interviewer: okay

Respondent: Were you circumcised?

Interviewer: I don’t know.

Respondent: You don’t know! Okay. Of course, you will do it when the time comes. They circumcised me. And I didn’t go to the doctor to give birth. Every child I have were born in the house. They actually circumcised me. I said it too. I said they circumcised me. You now say it should be done by doctor.

Interviewer: Hmm

Respondent: Is it a doctor's job? Surgery is a doctor’s job. We are not angry. It is about our forefathers that you wanted to take the female circumcision out of our hands. what are you going to give us, government? What are you going to give us? We were promised a program.

Interviewer: Hmm! Were these efforts successful?

Respondent: How can it be successful when the government is not faithful to its promises. Do you think we are fools? We are not foolish.

There was another program that we went to at ibadan. We were celebrating with the hope of getting what we were promised.

Interviewer: Let me cut you here. What did the government promise you?

Respondent: As at that time we were promised to be empowered after we told them that we have agreed to stop FGMC. Afterall it’s not my girls that they will turn into dog through promiscuity.

Interviewer: Hmm, hmm

Respondent: You see, what we’ll eat, will sometimes not make us wise. See what ladies are turning themselves into. Women are the one looking for men now. Women are looking for men, ladies now turn to dogs.

Interviewer: Hmm

Respondent: Why won't girls turn to dog? The clitoris of the female is equivalent to the penis of the male. The way a male penis rises is the same way the female clitoris rises. So, if the clitoris is not cut, women will be too sexually sensitive which can be an embarrassment to her ( nymphomania). Any girl that has it in her genitals will be looking for men around (promiscuity). Though among those who are circumcised, some are promiscuous. Did you get it now? If a circumcised person is like that, what will be the situation of woman who wasn’t circumcised.

Interviewer: I’m getting it ma.

Respondent: Those who are doing it are shouting around. They arrested someone when we are traveling to Abuja. The security operatives pretended as if they wanted to circumcise their girl child. As the TBA puts the tray on the floor, and he laid the child on it. They ask him to wait and stop.

Interviewer: it was a set up!

Respondent: Yes. They were telling him that FGM/C is abolished by law, he’s doing it, so they arrested him.

Interviewer: Hmm

Respondent: he was taken to the police station. Behold, the same TBA that was brought to the station circumcised the son of their boss three days ago. The boss immediately said no this is not the type of people we are looking for. He was released immediately.

Interviewer: you mean the law enforcement agents that are supposed to enforce the prohibition act are complacent.

Responder: I just told you

Interviewer: Thanks for your words since. I said before you go, what are they doing with snail’s shell?

Respondent: You want to learn? (Smiles)

Interviewer: It is important that one knows it. I’ve wanted to ask

Respondent: Yes, they have been trying to figure out how to do circumcision and we are successful

Interviewer: Hmm

Respondent: And we know that once in a while there may be no customers, it happens like that sometimes. At present, the TBAs now have government approved instrument of circumcision, apart from the ones we were using before.

Interviewer: You have really made frantic efforts to push through.

Respondent: If you circumcise a girl, she will definitely give birth smoothly. It is not true that a circumcised woman goes through caesarean section to deliver their babies. I do not also agree that a woman can have vaginal tears during delivery. P2 A70 PE

Are your mother grandmother and great grandmother not circumcised? Did she not give birth to you?

Interviewer: she did

Respondent: I that am talking here was circumcised. And I have never given birth in the hospital, all the children given to me by God, not once or twice

Interviewer: Hmm

Respondent: Where does the law come from? That is why they did caesarean section for people. That’s why they did, you see, there are some diseases that some doctors are not aware of. And that’s why they do caesarean section for some pregnant women

Interviewer: Thank you so much for your responses. As the community leader, can you encourage women to allow their girl children to be circumcised?

Respondent: Yes, but I will not force you. In the days of our mothers, they were aware of it, and they know how they go around it.

Interviewer: Hmm

Respondent: Nothing last forever in it. But the whole Nigerian culture has been taken out to foreign countries. It’s we TBAs alone that has out smart them now. Listen, as long as life exists, I will not destroy it.

Interviewer: Hmm

Respondent: How many do they want to arrest? There are TBAs that are more educated than them, that are even more exposed than them.

Interviewer: Hmm

Respondent: After all we didn’t collect salary from the government. We are doing our work. Look at the number years we have launched our programme, that the white people came, the government has not yet redeemed the promises made.

Interviewer: What do you think the government can do abolish the dangerous trend of FGM/C?

Respondent: It’s not that TBAs will actually not make money

Interviewer: Hmm

Respondent: it will be difficult for the government to abolish female circumcision because it is a practise that was handed over to us from generation to generation.

Interviewer: Hmm

Respondent: it is not possible for Someone to stop us.

Interviewer: Please ma, can you explain to me other reasons why it will be difficult for the government to stop FGM/C?

Respondent: other reasons?

Interviewer: Yes ma

Respondent: apart from the fact that it is a cultural heritage, inside the book of Islam (Quran), the clitoris is considered as a dirt that must be removed on the 8th day of the birth of the baby.

Respondent: we get remuneration from the female circumcision. So if the government wants to stop it, we must also be collecting salary

Interviewer: Hmm, hmm

Respondent: We must have a monthly remuneration, for “Oloola”

Interviewer: I'm still coming to that

Respondent: Yes?

Interviewer: I said I'm coming to that

Respondent: Ehn ehn

Interviewer: That’s part of the additional questions I will ask you

Respondent: Ehn.

Interviewer: Let’s look at this way, it will be up to about 15 children, 10 to 15 children that get circumcised within a month.

Respondent: before civilisation, it was more than that. But now, we don’t take care of it any longer, it is only those who come. we don't advertise it

Interviewer: You said it earlier

Respondent: It is only those that are please with it come to us.

Interviewer: You said it earlier. Have you ever been involved in a governmental program to ensure that circumcision stops? Is there any time that you have participated or that have been involved at all.

Respondent: That's what I said when they brought the issue to me. The whites that came when we had a seminar at Ibadan. They mentioned it. The kings also said, "What do you want to do for them in place of collecting female circumcision from them?" And they said they will do something for them.

Interviewer: Hmm

Respondent: It should be obvious, we should be paid like government employees

Interviewer: So, the aftermath of the program, what came out of it, what benefit comes out of it.

Respondent: Nothing comes out of it, for about three to four years now

Interviewer: Just like

Respondent: There’s no TBAs that is really suffering

Interviewer: Yes

Respondent: But the origins of our ancestors, we should just end it like that. It won’t stop with me.

Interviewer: You know there are different types of circumcision. What type is it? Just tell me exactly, what type do you do among them all?

Respondent: The type the TBAs does. I have told you

Interviewer: You’ll just cut little part of clitoris? Type 1

Respondent: Just a little part of the clitoris. You will just little part of it like this. It’s not something much. It is just lime pinching a baby with fingernail.

Interviewer: okay ma

Respondent: One will slowly take it away. What is there, God has put it there. It’s not something much. Those who did not know about it, and they are doing it were the ones that cut it like that.

Interviewer: Cut it all off. In your experience, why, is it so important for a girl to be circumcised?

Respondent: I have told you the importance earlier

Interviewer: Why should we?

Respondent: Was it not that, for her to start having sex all around. For a man not to be enough for her.

Interviewer: If she’s circumcised, what are the benefits?

Respondent: She should be circumcised?

Interviewer: Yes

Respondent: She won’t be a prostitute,

Interviewer: Hmm

Respondent: She will be the person who will keep herself. We also know that they are liars. they said that a woman who is circumcised will not enjoy her husband.

Interviewer: Hmm, thank you

Respondent: Which one pays you? They are feeling as the men feels too, let’s not deceive ourselves. I have a woman. That woman, it was only me and her, and I have never had relations with any woman. The man who says so is lying. Do you understand me?

Interviewer: Hmm

Respondent: Those who have three, four are even doing it

Interviewer: Hmm

Respondent: That’s how it is for the woman too. What arose in man, is what we take away on the head of that of the woman. If you don’t take it away. If it touches his thigh like this. She will be for looking man around

Interviewer: Hmm

Respondent: She can even pay men do it with her. You can see for yourself what is happening around.

Interviewer: When we are through, I will tell you what I have experienced with this research.

Respondent: Ehn

Interviewer: Ah!. Hmm

Respondent: What did you say?

Interviewer: I said what I have heard concerning this very thing, it brings out the many different things. Do you know that recently we are hearing about the cases of rapes that it has increased? if it is done by men, but what of women who also rape men. That’s a point we picked from what you just said.

Respondent: That is what pleases them now

Interviewer: I'm even ashamed of it. Thank you, ma. That is for a woman, for the family, for such for a family. What benefit is there for a family, a family where a circumcised lady came from? And perhaps the community she came from, what are the benefits they get for circumcising a girl?

Respondent: Ah! The advantage the parents of girl children will peace of mind without fears of their girls bringing shameful acts that can stain the name of the family. When a man is going out and a woman going out, it is dangerous for both of them. Men can go out. And a man who is intelligent will limit his going out, so that he won’t implicate his wife. But if the wife is going out, and the husband is going out, then you both will eventually meet each other.

Interviewer: you mean it is better to circumcise the female to prevent her from being promiscuous

Response: that is what I am saying. It is very important to circumcise the girl child. She is going to be a role model to the children because generally women are closer to the children. Any questionable behaviour such as promiscuity should be prevented. Thereby preserving the image or name of the family.

Interviewer: it has been shown that performing female circumcision on girl child does not allow them to enjoy sex later in life. Please can you shed light on this?

Respondent: that is one of the lies the government is peddling about circumcision. But they have forgotten that it’s only a minute part of the clitoris that is cut and not the whole clitoris. it is not true at all.

Interviewer: What about the children? Thank you, ma. Despite the experience you have had in this field as an heir, what is your perspective about this, though you have said it earlier, but I still want you to explicitly say what your perspective about stopping female circumcision is.

Respondent: That they should not be circumcised?

Interviewer: Yes

Respondent: Ah!, I am not in support of the government stopping the girl child circumcision. That has been the campaign and I am not hiding it. At least, it will not happen in my time. Anyone who is pleased can leave her daughter uncircumcised. The health professionals cannot do the female circumcision because they do not know what precisely to cut and what not to cut.

Interviewer: Hmm

Respondent: That’s how that part is

Interviewer: Hmm

Respondent: It is not compulsory. We, we will not advertise circumcision. No one is going around, advertising it. And if they bring child for TBAs for female circumcision, we’ll ask you “have you told the government about it?” did they agreed that you should do it? You own your child; it is a choice for you. It is a choice; it is not compulsory. No one is carrying circumcision around

Interviewer: Hmm

Respondent: Even boys, it is whoever that it pleases, should bring him to TBAs. We are not hungry and not really satisfied. But it’s our job.

Interviewer: As a leader in this community, what is the people’s perspective towards it

Respondent: Education has been having a negative impact on female circumcision because many of our children are well-educated now, they cannot perform female circumcision

Interviewer: Hmm

Respondent: If they see it, they can do it, if they don't see it, then they can't do it

Interviewer: Hmm

Respondent: Do you understand me?

Interviewer: Yes ma

Respondent: How much are we collecting? What is up to the wealth that God gives a person? They should come to our houses and see how well we are living.

Interviewer: Hmm. Thank you, ma. Thank you. Thank you for your time. I don't know if there is anything more that you like to share with me.

Respondent: Ah! I have nothing more to offer you than that. You are taking this to the government?

Interviewer: I am not taking it to the government. The work, I am a student. Alhaja, that’s why I explained it to you that I am a student

Respondent: That’s true.

Interviewer: I'm not from the government. Apart from receiving permission from the government. And if I didn’t obtain the permission, they won’t allow me to start the work in my school. I sent it to them in school. And they also gave me permission to continue. So, I'm not from the government

Respondent: You see circumcision. Those who are saying it. They understand what they are saying. Some people are here that are government officials that they sent the information to them. And the white also send money to them. It is part of the secret that was revealed when we are in Abuja.

Interviewer: what secret ma?

Respondent: The secret that they have collected funds and benefitting from it with the fake TBAs without benefitting us. The TBAs you now see are the real ones; you didn’t do anything for us. They saw us on that day, and they were surprised.

Interviewer: why would the organisers be surprised?

Respondent: They didn’t know that we are many and they didn’t know that we would come. It is the representative of each city were around here.

Interviewer: Thank you for your time. God will bless your old age ma. May God renew your strength. Thank you.

TBA 5B P3 A53 SM

Interviewer: Good evening, sir. My name is Omolara Fafowora. I am a student. I have come to interview you as a community leader and stakeholder in FGMC.

Respondent: you are welcome

Interviewer: I thank you for honouring me with your time sir.

Interviewer: can you please tell me your age?

Respondent: I am 53 years old.

Interviewer: sir, what is your level of education?

Respondent: I have a BSc certificate.

Interviewer: Do you live in this community?

Respondent: Yes. That is why I am able to continue this practice of circumcision.

Interviewer: can you please tell me for how long you have been living in this community?

Respondent: like I told you earlier, this is where I was born.

Interviewer: how did you become a TBA?

Respondent: it is being practised in my family. So, it is a kind of continuity in my family. Both men and women in my family culturally have their knives for circumcision for both male and female. Then only clause is that once a woman is married, she cannot perform FGMC again although she will still have her knives with her. But things are a different now because even those not from circumciser’s family are doing it now.

Interviewer: How did you manage to go to school if you have been practising this long?

Respondent: the government has made education easy by allowing people to go to school on part time basis.

Interviewer: As a TBA, permit me to ask if you have participated in any government efforts to stop FGMC.

Respondent: Government efforts to stop FGMC?

Interviewer: Yes sir.

Respondent: Well, I heard of it, but I didn’t take part

Interviewer: why did you say that sir?

Respondent: because of what I know, and I am sure of about girl child circumcision which I have told you.

Interviewer: Thank you sir.

Interviewer: In your opinion, why is it important for young girls to have this tradition done on them?

Respondent: Firstly, circumcision is our tradition in Yoruba land. There are different idols in Yoruba land and their mode of worship differs. In the worship of some idols, circumcising the young ones is an integral part of their worship. If you don’t follow this tradition, it may be dangerous. That is, the person involved may not be able to give birth and then you avoid the clitoris touching the head of the baby.

Interviewer: please explain further. You said that you are a Muslim. Is it also dangerous in Islamic religion?

Respondent:the first thing we know, and we are sure of is that any girl child that is not circumcised will be promiscuous. whereas a circumcised girl will not be promiscuous. There are times when parents bring their girls who are suspected to be wayward to us to know the status of circumcision. That’s one powerful point that we hold on to because there are different kinds of children. There are times when parents bring even the old girls to us, even though she was circumcised, they will want us to confirm because of her waywardness. Not long ago, I remember a particular time a girl was brought to us, it was reported that she was masturbating by inserting sticks into her private part (vagina). When they checked, it was then discovered that she was not circumcised. You see, the female genital is similar to that of the male it has a cover on it. If you check that of an uncircumcised male, there is usually some whitish spot around it. It is the same for female too but that of the male has a tiny hole where it can be pressed down. The female one does not have any hole, but it is usually blocked.

Interviewer: As a person who has been involved in FGMC for a long time, can you please explain the type of female circumcision you are involved in?

Respondent: Yes, I want to use that of male child to explain that of the female child. If it is someone who has grown without circumcision, the germs will be many and become black. That’s the way it is for the female too. The germs will grow under the skin, stretching the blood vessel and then this will begin to disturb the girl. So, circumcision is good to avoid promiscuity. That is apart from religion. Concerning the religion that we practice; Islam also support circumcision and we are not wiser than our religion. With these two points, it is important that we circumcise our children so that they can live peacefully in the nearest future.

Interviewer: if I get you right, you mean female circumcision is done to prevent infection and promiscuity.

Respondent: That is what I am telling you. Girl circumcision is done to prevent infection and promiscuity.

Interviewer: About the idol worship that you said earlier. You said you are no longer doing that, tell me more.

Respondent: Yes, because of the Islamic religion that we practice. Before now, we normally attend to all cases concerning tribal mark incision. When a child faints, they bring the child, and we incise tribal marks on him, and he will stop fainting. Also, for a child having convulsions, we will incise the child and put somethings there then the convulsions will stop. Those are the things that make the tradition of tribal marks important. Circumcision is done for the girl child in addition so that they will not be promiscuous. Girls have been brought here to be checked if they were circumcised because of their waywardness. People do circumcision to reduce promiscuity in the society. That is why people want to do it. It is our tradition and we found out it is dangerous if we don’t do it that’s why we are doing it.2

Interviewer: Based on your experience over the years, what is your opinion about stopping female circumcision?

Respondent: There is no one that is bigger than the Government. But my opinion is that the government should not stop female circumcision. Anyone that is interested in doing it should be allowed to do it and anyone that is not interested should be left alone.

Interviewer: Why do you think the government want female circumcision stopped?

Respondent: I read the reasons why the government is campaigning for an end to FGMC among which is the girl child will not be able to enjoy sex if circumcised. In our present society, we have ladies who are married and still have extra marital relationship and they have been circumcised. We have seen that those who have been circumcised but are into are into prostitution. If they don’t enjoy sex, will they be prostituting around?

Apart from the fact our religion is against the prohibition of circumcision, they also said it will hinder childbearing. The western people, are their children more in numbers than ours? In our society, some people have ten children. Can the white people have ten children? No, when they are not mad. They only have between two and three. There are people that have seven children, some of our mothers have about fifteen children without any problem. So, the reasons given by the government to stop female circumcision are not good enough.

You see, with the level of my education and with the circumcision that I am doing, there is nothing wrong with female circumcision. It is very beneficiary to us!

Interviewer: Sir, there are other vital reasons such as infection

Respondent: Yes, that is one major reason given by the white people that we considered important. In Yoruba land, there is a strong belief that a child does not die in the hands of the circumciser. The transmission of diseases/virus is not possible while circumcising because there are things that our fathers do to prevent transmission of disease. We don’t use the same instruments for two people, and we ensure that all instruments are cleaned before use.

Interviewer: What are the instruments used in circumcision?

Respondent: We use sharp objects. The doctors use scissor, but we use a sharp object like a knife. It has a long and flat blade.

The blacksmiths are the one who manufacture this object, I mean the local blacksmiths. Most of the children of the blacksmith around here have all left home. There is no blacksmith that knows how to make such object in Oshogbo anymore. We now go to Oyo to get the object. It is just the few of us that are around here that are still performing female circumcision. The children have left home.

Interviewer: thank you sir. Please tell me what you use in prevention of transmission of infection.

Respondent: We wash it in soap and rinse it very well and keep it in any disinfectant after which we rinse and wrap them in clean cloth. We have an association where all these things are being discussed.

Interviewer: Thank you sir. You said no one is bigger than the government. That means you stop circumcision because of the fear of government.

Respondent: The fear of government is the reason why we stopped circumcision. It is not because we don’t like doing it, but we don’t want to go against the law.

Interviewer: You said that you are no longer doing it but there are still people doing it?

Respondent: We don’t know those that are still doing it.

Interviewer: Sir, it has been confirmed that FGMC has not been completely stopped in Osun state.

Respondent: Although, I did not study law that’s why I don’t really understand all the laws against circumcision. I would like to beg the government to allow those who want to do it to go ahead and do it. As we speak, there are government hospitals that say they are not doing it again and we also have others who are still interested in doing it, but they are afraid of what the government will do. We are just begging the government because we cannot say one person is doing it here, another person is doing it there.

Interviewer: Can you advise parents to take their girl child for circumcision?

Respondent: I can’t advise anyone to do it but once a child is up to eighteen years, he/she has the right to do what he/she wants about his/her life. When a child is up to eighteen years and wants to be circumcised, then the elders can advise her to do it.

Interviewer: In your own opinion, do you feel everyone has stopped the tradition?

Respondent: No, not everyone has stopped it but when I don’t know those who are still doing it there is nothing, I can say about it. Going by what we are seeing in the society, the prohibition law has not been completely adhered to.

Interviewer: I like the answer you gave that not everyone has stopped this tradition and that the reasons the government gave are not strong ones. Can you tell me more?

Respondent: Not everyone has stopped it because the reasons are not strong enough. If a child has challenges in the future, it may be traced to the fact that child was not circumcised. Some people will say the head of new-born child should not touch the clitoris of the mother and so on. The Islamic religion supports circumcision although with specification of how far the cutting can be done. There are about two ways that it can be done in Islam. First, some only cut the head, some will cut off the skin covering the private part, but they won’t touch the blood vessel while some after opening up the skin will cut off the head of the blood vessel completely. Those are the three ways we do it. But what the tradition says is that the skin (clitoris) covering the private part be removed. Some people say if you don’t remove the skin (cliotris), just like the male organ grows, the female one grows too. If you allow it to grow and it starts touching her pants, it will begin to disturb her.

Interviewer: The tradition says we should remove the skin (clitoris).

Respondent: No, the tradition says you remove the skin (clitoris) and then cut it. But the tradition does not tell how to cut it down. Because there is still one other way. After removing the skin (clitoris) you will cut the blood vessel and anywhere around the private part where the blood vessel can resurface again will also be cut off. But our own tradition in Yoruba land does not permit that method, some other tribes do it. They go as far as shaving off the surface of the private part.

Interviewer: You said the government should reverse their decision and allow anyone interested person to be circumcised.

Respondent: The government should allow anyone that wants to be circumcised to be circumcised. Even during the old days, there are people who still did not do circumcision, I mean in my own tribe. There are tribes that don’t do it at all. But in my tribe, maybe due to lack of money or their carefree attitude because when I was young, they normally bring grown up people here to be circumcised.

Interviewer: I met a woman who said she was circumcised when she was about seven months pregnant so that her baby’s head will not touch the clitoris during delivery.

Respondent: Those days, grown up children are brought here when we were young. They no longer bring them here again.

Interviewer: How did you know the government is campaigning against this tradition? Through what medium did you hear?

Respondent: We heard through the radio

Interviewer: You said some elders were invited?

Respondent: Some of the elders who are educated were invited for seminars on the reasons why they should stop it. My personal opinion, what I see about the thing is that some of the elders collected money so that they can keep quiet, although, no one is above the law. The thing is not really from their mind but if they don’t take the money the government will still stop it anyway. We were called to a meeting in Ibadan, we bought uniform clothes, they promised to give people money and buy grinding machines for them. Several seminars were organized, they even came to Oshogbo for one of their meetings. Recently, Olorunda local government through the governor of Osun state called the people together again. Although the meeting did not yield any result because the real circumcisers were not present in the meeting. They just gathered some people and asked them to pretend as circumcisers, but they are fake. The government has tried.

Interviewer: Are you saying that the government only favours their own people who are not even circumciser?

Respondent: If the government is sincere about putting a stop to the female circumcision, the real traditional circumcisers would be involved not the ordinary people who are claiming to be circumcisers.

Interviewer: If I get you right sir, government efforts did not go a long way to reach those that are truly concerned in this matter.

Respondent: Yes.

Interviewer: What should be the right channel for the government to follow if they want to cancel the tradition of circumcising the female child?

Respondent: It’s hard to see a signpost of any circumciser these days. Except maybe you already know me, and you call me then I will come. That is how they come to us and say they want to be circumcised. We may not know them. Just the way you came, I will ask if it’s a girl or a boy, if you say it’s a girl, I will tell you to leave but if it’s a boy, then I will tell you to stay. Firstly, there is an adage that says it easier to collect what is a child’s hands than what is in his heart. I think the best way is to help the circumcisers and their children to be exposed to modern lifestyles. Secondly, someone who is wallowing in poverty and sees a way out it, definitely he will follow that way. Female circumcision is a major source of income for some TBAs, so it will be difficult for such people not to practice it again. Then, the government should continue to engage the media as means in conveying the message of putting an end to the female circumcision.

Interviewer: You were saying something about calling the circumciser together, exposing them to modern things. How can that be done?

Respondent: I have already mentioned one of the ways they can enlightened them. For instance, when they promise them anything they should fulfil the promise. I also notice that there are times when the government will fulfil those promises but those who received it will not distribute to the others because he is the one recognized by the government. There are many other things. The government is trying but their efforts is not reaching the concerned people. It’s difficult to see someone that says he will come to remote area for any circumcision. If you see any circumciser now and you asked him to sign an agreement and you give the necessary things, he will still be afraid. You signed an agreement, and you did not obey it. But when they bring the fake circumciser, they oblige them. I hope you understand

Interviewer: Tank you sir. You have talked about your part, how are the people in the society view the stopping of FGMC?

Respondent: The people’s opinion, let me first talk about the circumcisers before I forget. There is reason the circumcisers do not really support the government, to add to the other points, the circumcisers are sceptical that the government want to stop people from female circumcision because it is like a surgical operation, so that the doctors can start doing it and make more money. You understand? When we were young, there are many people coming for tribal mark incision but now, it has reduced because the doctors were not doing it in those days. Even if the doctors needed to do it then, they will still bring the person here. I remember that very well as young as I was because they don’t know how to do it. In fact, it can cause impotency in man. But when the doctors started knowing how to do circumcision for male, they don’t do for female. Some of them may be doing secretly, I know some doctors that we talk but they don’t do female circumcision. So, the circumcisers will be thinking that if they can ban that of female circumcision, that they may still ban that of male too saying it is an offence and you don’t have the right to do it. It may be thought that the government want to take away their means of livelihood. Right now, they have stopped that of female, although we have male customers because not everyone prefers the hospital, some people practice midwifery at home, in fact, I know some of them. I am now thinking that how much is the hospital charging that people are giving birth at home? But the circumcisers are afraid that the government will still ban that of male circumcision. But if there is an agreement that they will not stop the male circumcision, then that will be okay. Please say the last question you asked me again.

Interviewer: What is the opinion of the community members concerning FGMC?

Respondent: People’s opinion is two ways. The first one is that a child will be promiscuous without doing circumcision. Some people are not civilized, they follow the multitude. There are people that support the government, and we also see some people that will beg the circumciser to do it for them, they will promise not to tell anyone about it. Some people have stopped doing while some stood their ground claiming they will continue to do it may be due to their tradition or religion or they just insist on doing it but there are some who don’t do it again.

Interviewer: Thank you so much. Although you have mentioned some of the points, but I want you to add to it. You said government efforts don’t get to the right people. What can the government do?

Respondent: Another point is that many believed that the white people that brought the modern methods don’t do things for free. They believed there is something behind it, that’s food for thought for everybody. if there is no gain for the government, the government will not take it seriously. There are many things that we need in the communities that the government should be serious about, but they are not serious about it. Why is it circumcision they are now serious about?

Interviewer: What are the things the government should be serious about?

Respondent: There are many things. Firstly, let me start by saying that we don’t believe the white people. If the white people bring anything we don’t believe it because they have brought some other things before like lesbianism, gay marriage. We reject it and the government of Nigeria also rejected it, we don’t have faith in them anymore.

Interviewer: I know about breastfeeding babies, in the past, Nigerian mothers were breastfeeding their children well and the children were healthy. The whites have their children eat canned food and become very big, but they fall sick easily unlike Nigerian children that are breastfed. When it was first researched, canned food was introduced. At that time, it was that anyone that does not buy the canned food for her child should not give birth, so people stopped breastfeeding their children and started feeding them with the canned food. It was noticed that the children were growing without strength in them. So, we went back to breastfeeding our children.

Respondent: Yes, you are right. Another thing is that when they want to start the circumcision, you will pay cheap money to do it in the hospital but by the time they stop the native circumcisers from doing it, the knowledge will go into extinction and people will only go to the hospital for it. By then, the hospital will say it is an operation and they will charge big money for it. So, the people and the circumcisers are afraid that the money government have spent on publicizing this thing will be gotten back from them. People are afraid. Therefore, if the government want to cancel it, have already told you what they should do. If the government want to stop the female circumcision, the circumcisers should be empowered because an idle hand is the devil’s workshop. Another thing they can do is to have an agreement with the circumcisers to allow them to circumcise anyone that is interested in female circumcision doing it whether for the face or the private part. If it is the sharp objects used in the process of circumcision that the government is scared of, then they should organize a training seminar for the circumcisers on how to use and take care of the sharp objects. The circumcisers know how to take care of their instruments, but they said they are not satisfied with their method. They said infectious diseases can be transmitted through the instruments used by the circumcisers. But they should train them on how to use the instruments and teach them on the proper way of doing circumcision. But the TBAs are afraid of losing their fathers’ tradition.

Interviewer: As the community leader, do you know if the people are aware of that government programmes are still on, or the government just did it and stopped after some time?

Respondent: Yes, for the programmes, when one government administration comes on, they will shout and shout and after some time, they will stop. When another administration takes over, they will also make noise and after a while, they will be silent and that has been the trend.

Interviewer: Thanks a lot. Are there other things you like to add? If there is anything I did not ask you, but you think is necessary concerning female circumcision.

Respondent: Girl circumcision has been in existence here for a long time but there are some tribes that don’t do it. They are the ones who brought prostitution to our land through their promiscuity and so-called civilisation. In Yorubaland here, the type of care you render to new-born determines how he/she will live in the future. That is why we take extra caution. Female circumcision is part of the care that has been laid down for a girl child to prepare her for the future. No parent will want to be disgraced in future when it will be too late.

We don’t do prostitution in this land in those days when girl circumcision was being fully practised. People form those other tribes that we cannot mention come to the Yoruba land. But in this modern age, prostitution has become a common thing in our society, there is no shame about it anymore. Government even issue licenses to them. So, there is fear in the minds of the people that will prostitution not become what our children will be doing? When we see the tribe that don’t circumcise their female children and we see what they are doing up and down and has become a normal thing in the society. Even parent can obtain prostitution form for their daughters now. So, it’s something we are really worried about. Also, our religion is not against circumcision. That’s what we see about it.

Interviewer: You mean Islam supports circumcision?

Respondent: Islamic religion supports circumcision. There are three ways to do it, but Islamic religion is against two of those methods, only one of them has received acceptance by the Islamic faithful. This is one that says the skin (clitoris) be removed without cutting of the blood vessel. The clitoris is removed to clean all the dirt from the private part. That is what Islam supports and it is done everywhere they practice sharia law. In Nigeria, the Hausas are known for this act, they practice it very well. In the east, among the Igbo people, it is not well practiced but the Yorubas are in between. That is the way it is. The government is biased in their treatment of circumcision. They are strict with some people while they completely left some other people unpunished. And we are the same Nigeria operating under the same law. They don’t disturb them in the north the way they trouble us here. Hope you understand what I am trying to say. They disturb us here. The publicity is not as serious over there compared to what they do here so those are the challenges the circumcisers face in this part of the country.

Interviewer: Hmmm, thank you so much

TBA 3a ENGLISH P4 A86 PE

Interviewer: sir, you are likely to be of what age?

Respondent: around 86 years

Respondents: It is the issue of give and take. Everyone we circumcised is all alive today. No child can die in the hand of circumcision surgeon and all of our girls were circumcised. Here, our children are not having sex on the street because they were duly circumcised.

Interviewer: sir, it is said that females no longer enjoy sex when circumcised.

Respondents: it is only an assumption that circumcised women do not enjoy sex. When a circumcised woman has sexual intercourse with men, she will glue to it as if they tie penis and virginal together. Another woman they did not circumcise, it just like the penis is inserted into a big pot. And they will say, they are not enjoying sex. They have not encountered a man who is expert in sexual intercourse. If they live, they began to shake their bottom.

Now it has gotten to the level where women start to rape men, whereas it is the other way round that we have heard before.

I have a wife in this house, she said when she stood beside man, she began to romance them. It was her father that deflowered her, that’s terrible. As penis becomes hard, so also is women clitoris.

I was on program sometimes when a woman began to talk about her husband, what he will do for them, to help him then she begs me to come and marry her. The she duped a king almost about 4million naira. She wrote herself to that king that she wants to marry him, the woman was rich, and she came that she wants to marry another man. Now that the woman has come back to Nigeria from abroad, she has seen another man to marry.

The question is what can be done to arrest this situation. We receive white culture, and the world turned upside down. We are in the country where sex has no meaning. When there girl circumcision, did you see our ladies on the street? There is HIV everywhere killing youths, May God help our land. The world does not want us to speak the truth, but to lie. The white that says we should not do circumcision married to a circumcision surgeon in Iwo.

Interviewer: No sir.

Respondent: But now they engage in all forms of prostitution to make money. It is a physical benefit if you don’t circumcise because we are marrying prostitute now. Girl circumcision will prevent all these sexual vices such as prostitution and rape thereby preserving the image and honour of the family and the community at large.

I have been to several places, and I know what is going on in the outside world, in fact, I just came back from the UK. With my experiences nothing can change my position about female circumcision

Interviewer: thank you sir for the contribution.

Respondent: Have you gone to Oyo

Interviewer: No

Respondent: Go to Oyo and meet Alaafin. When we talk about king that knows history, Alaafin is one of them. White people have rubbished the world and they are doing exactly that. When they discover drug for HIV/AIDS, they didn’t allow its usage because they don’t want anyone else to produce it. Like my wife that her father deflowered, even in her husband’s house, he slept with her. And we want her father’s house to settle the matter. It was then that the father promised that he would not have sex with her daughter again.

On empowering the TBAs, the government of Kwara state said that they empower circumcision surgeon. You still want to hear; I went for a program, the Guardian Newspaper crew was there, we did it in Mapo. Kings were also there including Olubadan of Ibadanland.

I have issue with my health now, so I can’t say much, I defecated 25 times and they asked me to sleep.

TBA 1 round 2 P5 A63 SF

Interviewer: Good evening ma. My name is fafowora. I am here as a student researcher to ask you a few questions about eliminating FGMC in Osun state.

Respondent: you are welcome.

Interviewer: can I know your age ma?

Respondent: I am 63 years old

Interviewer: what is your level of education?

Respondent: I finished secondary school education.

Interviewer: I want to know if you live in this community and for how long have you been living here.

Respondent: I was born here and has been living here.

Interviewer: please tell me how long you have been a TBA.

Respondent: it has been a long time

Interviewer: like how many years ma

Respondent: it will be around 15 years now

Interviewer: Mummy is there any other work you are doing apart from being a TBA.

Respondent: I learnt tailoring work, but I don’t do it again. I only sew my own clothes.

Interview: As a community leader, how did you come about abandoning FGM/C?

Respondent: I learnt a lesson in a place that we should stop the practice of circumcising the girl child that it could affect the girl during childbirth.

Interviewer: I want to ask about circumcision for the girl. We know it is common practice for the male child. But we don’t know if it is still done for the girl child. One of them even said she will do it for her girl child because it stores dirt in her private part.

Respondent: I used to do it before, I can’t deceive you but since there is a new law. We normally go for meetings at living hope hospital in Ede because the doctor there is our leader that used to train us but because it is the government that said we should stop the practice, although it is a traditional practice, but we have stopped it. It’s been over eight years since I last did it. You understand. Some people will come insisting that should do it for them, but I said no because the law of the land is against it. We should always obey the law. There is something the doctors have seen before they said it should be stopped. Just like you have gone for nursing, except you are in the medical line, you may not really know the effects. What we know is that it usually affects our mothers when they want to give birth to children. When they have difficulties in childbearing then we can say something has been done to their private part. You know that our level of expertise differs.

A case was brought from Lagos to me that I had to work on it again but at the same time we are not allowed to do incision at home. But that of the boy I normally do it. Even some people say if they do it at the general hospital that it is not properly done and that they use ring in the process. I don’t know about that one and I have never done it. The ring will be inserted to bring out all the dirt from the private part. Some of these things may not heal properly and they will bring it here for me to do but God has been helping us. There are nurses who come for assistance when they give birth. About three years ago, someone call me to come do circumcision for her girl child in their house and I decline because I don’t move from house to house to do circumcision. I do that here, where God has placed me.

Interviewer: I am a student ma. You can see that I did not collect your name or number that can be used to identify you. Please ma I want you to relax with me, I am not here to arrest you.

Respondent: I know. I am only explaining things to you. When you bring your case to me here, I will pray for you and pray inside water with psalms. When you are in the labour room, there are certain things you can do and there are things you cannot do. We are not allowed to give injection to the pregnant women talk less of circumcising them. But the one I know and I’m confident about without any fear is that of boy child circumcision which i normally do. But for the girl child we are not allowed to do it. What they are emphasizing is that it usually affects them during childbearing. That’s the lesson I learnt form that. For the boy I do it. Some people say that if God want a girl’s circumcision, He would have done their private part like that of a boy. That’s the way I was taught. We were also told that it makes childbearing difficult for women. That’s what I learnt from a nurse.

Interviewer: Thanks for the explanation. Truly, we have heard that the practice of circumcising the girl should stop but in your own view and with your years of experience working with women do you feel that there is no benefit if we continue this practice.

Respondent: I don’t know any benefit in the practice, but some people complain that it causes itches in the private part if the girl child is not circumcised. The government said we should stop doing it and I don’t do it.

Interviewer: why should we circumcise girl? What are the reasons for it?

Respondent: It is not good to do it. The doctors said it is not good. Write it down. The professionals said it is not good to do it.

Interviewer: Even though they said it not good to do it, don’t you think some people still do it for their daughters?

Respondent: well, as for me I don’t do it and there is no way I can know what others are doing.

Interviewer: A friend of mine was saying earlier today that an elderly woman asked her neighbor to bring her daughter to be circumcised

Respondent: Let her go and do it

Interviewer: Although they said we should not do it again. Some people still feel it has advantage

Respondent: You have just said it. The person who says that should take her child to where they will do it for her. Someone brought her child from Ife-city sometimes ago. I told her I cannot do it after all she is going to pay me, but I said no. If you see where they will do it for you, you can take your child there. But the doctors have already said that we should not do it. We have attended seminar at least on two occasions that we should not do such thing again.

Interviewer: You mentioned that you collect money for female circumcision. Since you said that it is a service being rendered, why do you collect money ma?

Respondent: Ha! My child, it is also a source of income for me. Although I am a trained tailor, but I cannot have a single source of income. I don’t advertise, they brought their girl child for me to render my service and I get paid.

Interviewer: Thank you ma. Please, can you tell me how much you collect per circumcision?

Respondent: with the way things are now, it ranges between #1000-#1500

Interviewer: Apart from the seminars/training that you have attended, what other things do you think the government can do to help stop this practice completely.

Respondent: the government should intensify their efforts in enlightening the TBA by frequently organising seminars on issues concerning female circumcision. This is because the government is not doing enough. The seminar we went for was only organized once or twice and that is what they do when issues of public concerns arise.

Interviewer: Thank you ma. You mentioned the other time that you are no longer doing it. But for those who are still doing it what do you think the government can do so that they can stop?

Respondent: Listen, may God help us not all TBAs are known by the government. When you call me, I said I don’t know you except I see you.

Interviewer: Yes.

Respondent: if you don’t come here how can i recognize you. But I want you to know something just like what the bible said that if your heart does not condemn you. But what I know is that whatever the doctors said we should not do I believe it is bad. For me, I cannot begin to question the doctors why they said we should not do what they said we should not do.

Interviewer: I am not asking for the reason. You have not truly understood my question. I already said I am carrying out research.

Respondent: Yes, I know that.

Interviewer: It is true that they were doing it sometimes back, but they stopped it because the government said that they should not do it again. But we observe that some people are still doing it because they feel it is good, so I am asking that as a woman and someone who has been involved in this act before, is there any benefit in doing it because some people says that if you don’t do it for your girls they may become promiscuous and wayward.

Respondent: What they said is that if you circumcise a female child and you pour the water outside so that fowl and dog feed on it, such person will become promiscuous. That’s what I heard. Not the people that were not circumcised at all.

Interviewer: Okay

Respondent: Even in the case of tribal marks, after it has been done you must not pour the water in a public place like a gutter but inside soak away. Not just anywhere.

Interviewer: Like I said, some people still feel it is right for a girl to do it. Part of their reason is that if they don’t circumcise a girl child, she will be prone to promiscuity that before anyone moves near her, she is already excited sexually.

Respondent: Maybe that is the reason. We all have different nature, but it is what we are taught that I am doing. I did it for my children when they came here because it has not been stopped at the time.

Interviewer: So, the reason why they are doing it is because of culture not because of any major benefit

Respondent: We grew to know about circumcision. It is written in the Bible that on the eighth day the baby should be circumcised there is no specification if it is a boy or girl until the gonernment announced its prohibition. So that’s why we do it on the eighth day. There is nothing like infection transmission, bleeding and so many other things that are being said about female circumcision. It is just cultural to do it.

Interviewer: Okay, just like we christened a baby on the eighth day, so they believed the same for circumcision too. That’s why they do it for both boys and girls.

Respondent: Yes, on the eighth day we incise the children, but I don’t do it again. If you ask anyone around here, they will tell you that I am not doing again.

Interviewer: I know. I am a student doing research, I am not here to arrest anyone. That’s what my research is all about; I only want to know if there is any benefit derived from it.

Respondent: The doctors are the ones that should explain this to you. We were not told the benefit. They only emphasized on the disadvantages.

Interviewer: The doctors only emphasized the evil in it without talking about the benefits but those who are doing it outside still feels there are some benefits in it that’s why they keep doing it. But after the government stopped it, what is the reaction of the masses about it?

Respondent: There are places where they stopped the practice after the government has banned it. If you give birth in this place now, you are free to go with your baby. I will not ask you to do anything after that. But you are free to do it outside. That is up to you. Once you give birth here safely and sound, that is all that matter. The reason why some people want to do their tribal marks here is because they see it on others, and they liked it and wants me to do for them too. It’s someone who told you about me.

Interviewer: One of my friends said that her daughter is itching her vagina because they said that it is not good to circumcise girl.

Respondent: If that is the reason why are there many promiscuous young people outside, may God speak to the doctors to change their mind to allow people to start doing it again. Because I don’t know why they said they should not do it.

Interviewer: You don’t know why the doctors said that, but you are just following the word of the bible. But you said that you attended a seminar on abandoning FGMC

Respondent: I am just following the law of the government; we really don’t know why they stopped it. Some years back, there was nothing like government hospital, just like Baba Living Hope said that he was born in a midwife house. He has become great today. Only God knows the keys to this work. But we don’t know why they stopped this practice.

Interviewer: Thank you mummy. I am rounding up, just two more questions. Part of what the government has put in place to stop the FGM/C is that people like you who are TBAs are invited to the hospital where they educate you on what to do. But for the populace, what is the government doing to educate them on the danger of circumcision?

Respondent: I don’t know what the government is doing.

Interviewer: What of what they can do? What do you think the government can do?

Respondent: I don’t know what the government can do to stop it.

Interviewer: For instance, what if they announce it on the radio and television?

Respondent: You know when we have seminar; Covid-19 is the reason why we didn’t do the seminar last year. When we have seminar, they will print the booklet so that everything they will say will be on it for us to read. We don’t know why they said we should not do it again. I kept repeating this statement. I am sure there is a reason why they said it should be stopped. Probably they have read a book on it. I’m sure they should teach you people about these things. You understand? They said don’t do this thing because of the danger in it.

Interviewer: what is the danger in it?

Respondent: They said it causes a lot of difficulties during childbirth. That is the only reason they told us. Were you not taught all these things?

Interviewer: Now I understand. Last question so that I can write it well. I realize that you understand that the government does not want you to do it. For those who are not TBAs like you but mothers, in what way is the government reaching out to them to make sure they are properly educated?

Respondent: They announce it on radio sometimes, but you know somebody like me I have not switched on the radio since, but people are aware of it. Only few people will say they don’t know about it. Almost everyone is aware.

Interviewer: As they are announcing it on the radio, some people are in support of it while some people do not like it at all. For those who are still against stopping child circumcision what do you think the government can do about them?

Respondent: They should keep announcing it until they agree. It is through the announcement that they people will come to terms with the idea. Those who are still doing have their reasons and those of us who have stopped are just following the law and we shall have the benefits of following the law. Hope you understand. May God help all of us. The law is very important for us to follow. Just yesterday, I was speaking with of the senior nurse at the hospital telling her that they are no longer doing seminars and workshops like before and she said she heard me. All of that is so everything can work out well. We have to keep improving on the work so that pregnant women will deliver safely. That’s why they educate us.

Interviewer: Okay ma, as a woman that was circumcised, can you tell the difference between when circumcised and when not?

Respondent: There is no way I can know the difference when I don’t know those who are not circumcised.

Interviewer: So, there is no one around you that you have seen so that you can tell the difference?

Respondent: When I don’t know those affected by not being circumcised. No one has complained to me about any issue about circumcision.

Interviewer: What is your view about female circumcision? Do you want it stopped or not?

Respondent: The government will decide that.

Interviewer: But what is your personal view about it? Do you feel it should be stopped?

Respondent: As it is, it does not hinder my own work. Whether you circumcised your child or not, that’s not my problem. My job is to obey the law of the government.

Interviewer: Apart from the law now. Do you fell it should be stopped?

Respondent: I cannot decide by myself. That question is different.

Interviewer: I am only asking for your opinion?

Respondent: Though my opinion does not overrule that of government, but I honestly wish that the government does not stop girl circumcision to avoid negative effects of leaving the clitoris uncut. Besides, female circumcision has been part of the routine care for the new-born girls to prepare them for the future. The things that we do before without any repercussion, now that there is a law, we can’t do it anyhow again.

Interviewer: But sometimes when the government makes a law, like now that they close the border to stop all importation so that we can promote local made products. Some people are not happy about it while others are happy about it. But you know that not all laws from the government are usually right. Some people may be happy about it while some will not be. For instance, the border is closed in the south here and goods are expensive, but the case is different in the north. So, not all government law is right.

Respondent: Laws about the body is different from the law about goods and commodities. Someone who is sick with a cancerous sickness and the government has made laws concerning the sickness, but you choose to consult a local nurse. You have only done that to you own detriment. But I keep saying that they know what they see for them to have said that it should be stopped. Although they really did not tell us why other than the fact that it causes difficulties during childbirth but, it depends on the individual. As everything is going on fine may he continue like that. Jesus will continue to help us.

Interviewer: Amen. Thank you, ma. So, the training they gave you and the announcement on radio is what has been done so far.

Respondent: Yes, the announcement on radio and the training given to us. No one can say he/she has not heard about it. Other small hospitals maybe doing it, but I don’t do it. It’s been over ten years I last did that.

Interviewer: Thank you ma. May God continue to help you. We don’t know whether they still announce it on radio.

Respondent: I am sure people listen to radio a lot. Include it in your research also, the government should keep announcing it on the radio and television. The rest is in their hand. I heard it on the radio because I do listen to radio and even you can hear it on NTA, that’s the general television station for all

Interviewer: Did you campaign for the government on stopping child circumcision.

Respondent: I did not campaign for the government. My body is already old. I just want you know that whatever government said we should not I don’t do it. I follow whatever instructions given.

But anything that will affect our body and we have been warned not to do it. As for me I won’t do it.

Interviewer: Thank you ma

Respondent: All the law is to our own benefit. They may not tell us the details of everything. But they said it is dangerous and that’s what they emphasize.

Interviewer: Amen in Jesus name. Thank you, ma. You ministry will not fail. God bless you ma.

TBA 4 round 2 P6 A80 PE

Interviewer: Good morning ma. I am fafowora, a student who is carrying out study on the elimination of women-to-women intergenerational FGMC of girls.

Respondent: you are welcome

Interviewer: ma, I want to know if you have any form of formal education.

Respondent: Yes, a little. I have primary education. In those days there was not much emphasis on education.

Interviewer: do you live in this community and for how long ma?

Respondent: this where I was born. Though at a point, I travelled with my aunt, but I still came back and married here.

Interviewer: can you please tell me your age?

Respondent: Ha! Hmm! You can see it for yourself but thank God for keeping me and maintaining me. I will be around 80 years of age.

Interviewer: As a community leader, have you ever participated in any government efforts to stop FGM/C in osun state?

Respondent: not at all. I only heard it on the radio that female circumcision should be stopped.

Interviewer: What are the benefits of circumcising a girl?

Respondent: The benefits are, you see, in the olden days when we are young, they used to tell us that the head of the child must not touch the clitoris of the mother that the baby will die.

Interviewer: What of nowadays?

Respondent: In these modern times if you agree with it. We pray our children will not die. We don’t know the kind of diseases that people are facing these days. I am the one that cut for my girls and their children. (intergenerational) Before you cut that of a boy child, you must comb and remove the hair if he’s grown up. Because there may be maggot there. It is the female one that doesn’t have maggot. So, you wash the dirt first.

Interviewer: You said that you were the one that cut for your girl children and grand girl children. Can I get to interview them?

Respondent: they are not staying around here. They do not have any problem that they are all doing well and did not develop any of the issues they are saying around. They are all alive and doing well.

Interviewer: Part of the reasons why we must circumcise the female child is that the head of the baby must not touch clitoris of the mother.

Respondent: Yes, it must not touch it. If the baby head touches the clitoris that’s why some people say that the baby die, that the baby will not last seven days. That’s why we circumcised the baby, both boy and girl. You can’t give birth to a girl without circumcising that child. Some people will say there is no need for the female circumcision. What if there is an accident and the hair on the head mistakenly touch the clitoris?

Interviewer: Apart from this, what other benefits are there?

Respondent: So that the baby will not die by touching the clitoris.

Interviewer: Is that the only benefit there in?

Respondent: That’s the only benefit in it.

Interviewer: What will happen if the girl child is not circumcised?

Respondent: It is not a good thing if girl child is not circumcised, if you don’t circumcise girl child when she gives birth and the baby’s head touches the clitoris, the baby will die. This is because it is a taboo for the baby’s head to touch the mother’s clitoris. That’s why some babies die during childbirth and the parent don’t know why. Because they don’t understand why. When a woman is in labour and the baby is about to come and touches the clitoris, that can result into death.

Interviewer: Thank you Grandma. Some people say that if a girl is not circumcised, she will be promiscuous, is that true?

Respondent: Yes, uncircumcised girl can be promiscuous because of the sexual excitement, she will not be easily satisfied with a man so she will be moving from one man to another seeking sexual satisfaction. A circumcised girl can also be promiscuous if the part of the clitoris that is cut is not properly buried. It depends on the way the circumcision is handled. When you are done with the circumcision, you must bury the waste very well. If not, if the chicken digs it out, that girl will be promiscuous running after guys. After the circumcision has been done, the mother must ensure to dig deep into the ground and put the waste there and then cover it with the earth very well, if possible, match your legs on it. In so doing, the chickens will not be able to bring it out again and the girl will not be running after men.

Interviewer: Thank you ma. Is there any advantage for the family, the community and society at large?

Respondent: girl circumcision assists in raising decent girls that would not be tempted to be wayward. When a girl is circumcised, the excessive sexual excitement is reduced making her to be less prone to promiscuity.

Respondent: Those days when they were shouting that circumcision should be stopped for the male and female child. That of female is even dangerous. If you have daughter and you don’t take care of her, don’t you know that the vaginal is the same as the penis? I am trying to explain some things to you because you came here to learn. If you leave the clitoris of the female child, she will be promiscuous.

Interviewer: As a community leader, do you agree with the government stopping the circumcision of the girl child?

Respondent: No, I don’t agree with the government’s decision. The end is not good. As for me, I did it for my children both male and female. None of my children is promiscuous because I circumcised them and took care of them all. I bury it properly so that chicken will not be able to unearth it. Once it is unearthed, that is embarrassment for the girl.

Interviewer: So, you don’t agree with the government?

Respondent: No, I don’t agree with the government on the issue of girl circumcision.

Interviewer: why don’t you agree ma?

Respondent: you people think we don’t know what we are doing. We are also very vast in knowledge and highly influential. I have been performing female circumcision without any negative issues, so it is not possible to trick me into stopping it. Not just possible

Interviewer: Why do you think the government would want it stopped?

Respondent: Some people don’t know how to do it. They can cut deep into the flesh, and it will become sore and painful. That is why they said we must stop circumcising the girl child. So, if they don’t do it well it will become a problem. When I did it for my children, it was done well without any complication. They are not asking for sex around looking for who to sleep with. There is none I circumcised that became promiscuous. So, it is about how you do the circumcision and how you take of it. That’s why they said we should stop FGM/C. But for those who are wise and intelligent and skilful in doing circumcision, there is nothing bad if they do it. It is to reduce the problem of the world. If you leave it like that the child will be promiscuous

Interviewer: What should we tell the government?

Respondent: I do not support the government stopping the girl circumcision as it is part of the treatment and care of the baby to prepare them for the future. We were also told that female circumcision causes infections which is not true at all! Honestly, I don’t know what the government want to gain from stopping it.

Interviewer: They should not stop circumcision?

Respondent: They should not stop it. POSITIVE ATTITUDE

Interviewer: As a community leader, can you advise someone to take his/her girl child for circumcision?

Respondent: Yes, I can advise someone, and I will start with you. When your children start having girls ensure you do it for them. Give the baby to the person that can do it well so that there won’t be complications. Hope you understand what I am saying? Make sure you remove the waste including the water, don’t let the water pour on the ground so that chickens will swallow it. So, take care of it very well. When you are doing it put a big bowl under your legs so that the water will pour inside it to avoid spilling on the ground. Do you understand me? Because I don’t know you and you don’t know me, but God knows you. So, if you do it that way there won’t be any complications. POSITIVE PERCEPTION

Interviewer: In this area, do people still circumcise their children?

Respondent: Some people don’t agree to do it for their children. Their refusal to do it is not good because they don’t know what it can cause for the child. If they know the effects of not doing it, they will change their mind about it. The reason why they stopped that of female is that they don’t know how to do it.

Interviewer: Government should train and teach those who are not doing well so that they can start doing it well.

Respondent: They can teach those who are interested in learning how to do it properly so that they can do it for others and for their own children. Those who will do it must be cleaned and skilfully trained for it.

Interviewer: Thank you ma, thank you so much. May you enjoy your old age. I am so happy I met you because you have the experience of circumcision.

Interviewer: Grandma, as a person that has been involved in FGM/C for a long time, tell me about it and the type you perform.

Respondent: Do you want me to explain to you?

Interviewer: Yes ma.

Respondent: I do circumcision for both boys and girls. They bring them from the village to me and I will collect the necessary things and start the procedure. Once the circumcision is done, whether for a boy or a girl, we don’t wash it until it has healed completely. After I have done all that is needed to be done then they can wash it and that’s all. Nothing will happen after that.

The important thing is that we must buy snail. Snail is the cure for the pain of circumcision and control of bleeding.

Interviewer: What other things do you use in the process of doing circumcision for people?

Respondent: We use knife for the incision. We don’t use such knife to cut anything like wood or something else. We don’t use it again

Interviewer: Will you throw the knife away after you have used it to circumcise one person?

Respondent: No, if you don’t have the big knife and you use small ones like razor blade, you must dispose it after use. You don’t use it again for another person.

Interviewer: What if it is a knife that you used for the circumcision?

Respondent: For the big knife, there was one that we normally use then, we call it ‘’Ajoyin’’ you can sharpen it again. It got lost in my house then. Then, I will pick it for circumcision, my eyes defect does not mean I cannot do circumcision.

Interviewer: I want you to explain that of female circumcision.

Respondent: For the girl, it is the same process, you make the baby lie with her back on your laps, then you take something like a needle, you will notice that there are two tiny things there, when you want to do that, you will use a blade to cut it gently so that its head will not grow big like yam. Hope you understand, so when you cut the tiny head in between it. Have you not seen what I am talking about before?

Interviewer: I have seen it ma.

Respondent: If you don’t cut those tiny little things, overtime it will become so big that it will start affecting the private part. Once you cut those tiny things besides the vagina. Please listen. I hope you are not in a hurry.

Interviewer: No ma, I came to meet you.

Respondent: Okay ma. So, once you cut the tiny things, you must do it gently so that it won’t cut the flesh with it to avoid bleeding. Once you cut the flesh, it will cause bleeding. You cut gently and carefully. Although blood will come but not as much as when you cut the flesh with it. You also cut the second one too. Even when you want to cut the private part, you must avoid cutting from down so that you won’t cut the vagina flesh with it, if you cut from down, it may result into pain. Hope you understand?

Interviewer: So that means circumcision is done on two parts of the vagina?

Respondent: No, if this is a vagina, the tiny things will be on one side here and the other side there. You must not say that it is too big, and you want to cut from the bottom that means you want to kill the baby or take her to the hospital. Do you know what they call ‘’ido’’, that is the clitoris? So, you carefully cut the clitoris. Once you cut for one side, you don’t go back there again. In the olden days, no one reckon with the foreign method because it looks difficult. They say we should not cut the clitoris. I did for all my children, both boys and girls.

Interviewer: Is it good to circumcise the girl child?

Respondent: There is nothing wrong with it. Just listen to me. I am not talking with my eyes; it is my mouth and I know what I am doing. For the girl child, we do. You must not cut the private part anyhow. What you are going to cut is already there and you will do it carefully well. Hope you understand?

Respondent: I did for all my children and the children of the women here with me.

Interviewer: At what age do you normally do circumcision for the children? Is it when they are old?

Respondent: We normally do it at a tender age because it will be painful when they are getting old. I usually do it a day after they have been christened. I also don’t wash the circumcised part until it has healed completely. If I, do it for someone else baby, I won’t wash it. After I have done the necessary thing, I will collect snail.

Interviewer: What do you use the snail to do?

Respondent: After doing the circumcision, you will put the water from the snail on the place you have cut for the male child and even for the female child, you will put the water from the snail on the surface. I have done it for a lot of people. A lot of people bring their children to me for circumcision. I can charge from eight hundred naira to one thousand and two hundred naira. So, when you come for circumcision, I will ask for the snail. You must not eat the snail after the circumcision. After using the water from the snail, the mother must not eat the snail, or any other family must not eat it.

Interviewer: sorry to cut you ma. Is the circumcision the only source of income since you have many mothers patronising you?

Respondent: Yes. Sincerely it has been a source of income to me because I have been into circumcision for a very long time. And since many people patronises me, I make good money out of it. But at the same time, I engage in petty trading too.

Interviewer: Hope you get what I am saying and hope I am not taking your time.

Interviewer: No ma. I should thank you for giving your time.

Respondent: So, if the mother eats the snail, it will affect the baby. That’s why we don’t give the snail to the parent to take home after circumcision because some of them are not self-disciplined. When the circumcision has been done, I will break the snail from the bottom and let the water from the snail drop on the circumcised part. When everything is done neatly and carefully, then there won’t be any complication. When you break the snail from the bottom, only the white portion of water from the snail should drop on the circumcised part. Don’t let the black water from the snail drop on circumcised part. If the black water drops on it, it will not be fine. I am teaching you all these if you want to do it for your children by yourself. I don’t buy drugs; I believe in herbs, and it is working.

Interviewer: Thank you Grandma.

Respondent: People come from different places to circumcise their children here. There is a particular woman, she will gather children together and bring them to me for circumcision. Both boys and girls. Even grown-ups too. They brought them here. If they are five children, they will buy five snails. They already know me. I won’t charge exorbitant amount. Just like one woman said, she said she will not allow any other person to circumcise her children except me. I don’t wash the wound until it has healed. When people start washing the wounds that’s how different problems start showing up. Once you do the circumcision, don’t wash it, put everything needed and then wrap it with a bandage, after five days it will heal no need of washing. After that, there is that of the herb that you can do also. I hope I am not taking you too far. We also use leaves together with the snail water.

Interviewer: are you aware that the government has stopped the female circumcision?

Respondent: yes I am aware. Let me ask you also, has it stopped? Because if it has stopped, you will not be here.

Interviewer: You are right ma. It has not been stopped ma.

Interviewer: having said all what you have said ma, what are the steps you think the government can take to stop this practise?

Respondent: You know that female circumcision is our cultural heritage and identity. According to tradition, some people are saddled with such responsibility in the community. If the government want to have anything to do with female circumcision, these people must be reached. You in particular came to us when you know where to go to.

Respondent: Yes. I am talking of the herbs not modern medicine.

Interviewer: Thank you grandma for your time. If I have any course to come back to you, I hope you will oblige me.

Respondent: Why not? You are always welcome.

TBA 2 b INTERVIEW P7 A73 SM

TBA 2 Muslim Male Secondary 73 years 55 years

Interviewer: good evening, sir. My name is fafowora omolara. I am student carrying out research on the elimination of women-to-women intergenerational FGM/C of girls in osun state. I want to interview you as a community leader.

Respondent: you are well come. I have been informed of your visit.

Interviewer: thank you sir. Before I move to the main questions that I have for you sir, I wish to know your age.

Respondent: I am about 73 years old

Interviewer: Thank you sir. what is your level of education sir?

Respondent: modern school. Equivalent of the present secondary school.

Interviewer: Do you live in this community and for how long have you been living here?

Respondent: it has been a very long time.

Interviewer: Can you please tell me how you arrived at being a TBA?

Respondent: it was inherited from my father.

Interviewer: sir, tell me, do you have any other source of income

Respondent: Yes. I am a farmer.

Interviewer: In your opinion as a TBA and community leader, what do you have to say about FGMC?

Respondent: Girl circumcision has been in existence from time immemorial. Therefore, it can be described as our cultural identity that was transmitted from generation to generation.

Interviewer: Thank you sir. I want to find out from you why it is not permissible to circumcise a girl child.

Respondent: It is whatever the government commands us to do that we will follow.

Interviewer: Hmm

Respondent: The government said that FGMC is not good, and we have agreed with them that it is not good.

Interviewer: Despite the fact that it is our culture, we agree with the government that it is not good?

Respondent: It is mandatory, since it is the government that commands that we should stop circumcising the girl child.

Interviewer: Was it because the government forced you to stop, or you are afraid of law? Since you inherited the practice, do we stop circumcision for a girl child in your opinion.

Respondent: It is what the government commands us to do that what we did

Interviewer: I understand

Respondent: We’ve agreed to it like that

Interviewer: You have agreed to it like that?

Respondent: They said we should stop doing it. And we have obeyed the government.

Interviewer: I understand but what I am saying is that as it is part of our ancient culture. Truthfully, is it good not to circumcised girl child.

Respondent: There is nothing harmful in FGMC. It is good. It is the government that knows what they saw that made them to stop it.

Interviewer: Hmm

Respondent: It is the government that knows the side effect. We cannot say in precise

Interviewer: Okay. What is the side effect?

Respondent: We cannot say precisely what the side effect is. It is the government that knows the side effect. Who knows what the side effect may be that made them to stop girl circumcision?

Interviewer: I understand sir. So that is why you stopped it because there is no benefit in it

Respondent: There is no benefit in it

Interviewer: What if we put that of government aside. You know there are many forms of government. Like we know that in those days parents do breastfeed their babies

Respondent: Yes

Interviewer: But the whites don’t have time. They feed their babies with food. And at a point in time, they stylishly introduced us to start feeding our babies too with artificial baby foods.

Respondent: Yes

Interviewer: We now get to know that their kids are not strong, they are only big

Respondent: Yes

Interviewer: But our kids here, though they are not big, but they are healthy, and they don’t get sick.

Respondent: Yes

Interviewer: Later on, they now said that people should be buying the food. Then people started buying the food too. And our kids too now began to be like the whites’ kids

Respondent: Yes

Interviewer: it turned out that they will become big and not strong. They began to faint. Later they realised that there’s something in the breast milk, so they turn us back to it

Respondent: Back to breast feeding

Interviewer: Back to breast feeding. That it is the one with more nutrient than the baby food

Respondent: Than food

Interviewer: So, I now see to it that. Although the government may bring in some new culture. Is it true that we discovered that there is benefit in circumcising a girl child?

Respondent: I personally don’t see any benefit in it.

Interviewer: Okay. What is the reason why they are doing it in those days?

Respondent: It is what our forefathers were doing that we inherit from them.

Interviewer: Okay. It is simply because they were doing it, that is why we are doing it too. There is no benefit in it at all.

Respondent: There isn’t

Interviewer: Okay. What are the programmes you think that the government has put in place to stop FGMC?

Respondent: to stop

Interviewer: Yes, to Stop girl circumcision.

Respondent: The government should keep enlightening people on the radio which covers a wider population. I also heard it on the radio.

Interviewer: Hmm. You also heard it in the radio.

Respondent: It was on the radio.

Interviewer: And people agreed with it

Respondent: Yes

Interviewer: What is now the opinion of people to it.

Respondent: Since they are told to stop it. It is compulsory for them to stop it.

Interviewer: Hmm

Respondent: Although majority of us have stopped. There are still some that are still doing it. But we have stopped doing it.

Interviewer: for those that are still doing it. What do you think is still making them to continue in it?

Respondent: It is their wish. They wish to do it.

Interviewer: They wish to do it. I want to get something. I know they must have a reason that they see in it. whatever one is doing, it will have advantages and disadvantages. So what is the benefit that they are deriving from it that made them think that

Respondent: They should stop doing it.

Interviewer: That some people are still doing it. What did they see in it?

Respondent: Hmm. What happened is that our people have believed that female circumcision here is part of our culture. But the government has announced that we should not do it again.

Interviewer: Hmm

Respondent: The Igbo tribe for instance. They don’t do it for their children from the beginning

Interviewer: The Ijebu people don’t do it too.

Respondent: The Ijebu people too don’t do circumcision. Even Urobo people only do it when the female child is ready for marriage

Interviewer: Hmm

Respondent: But the Urobo people are doing circumcision.

Interviewer: So, the Urobo people always do circumcision

Respondent: Yes, they do it. But we, we have concluded that we should cancel it.

Interviewer: The Urobo people are still doing it?

Respondent: They are still doing it till this present time

Interviewer: So, what makes them to keep doing it then? Is it because it is part of culture?

Respondent: Yes. Culture

Interviewer: But I think before something becomes a culture, it must have a benefit that it has before considering it to be culture. I was looking at it that could there be no benefit they see in it at all apart from being cultural.

Respondent: I don’t see any benefit in it

Interviewer: Hmm

Respondent: If you do it, it doesn’t kill the child. And if you did not it, it doesn’t kill the child as well

Interviewer: Some people said that when circumcised girls grow up they do not enjoy sexual intimacy with their husbands. Is it true?

Respondent: It is possible, it is like that. And it is possible, it is not like that but me, I don’t do it again

Interviewer: You don’t do it again

Respondent: It’s been long ago that I have stop doing it.

Interviewer: Thank you sir. Concerning those that are still doing it. What do you think the government can do to stop it.

Respondent: They will keep saying it on the radio

Interviewer: Radio?

Respondent: Yes. They will keep announcing it on the radio till they stop.

Interviewer: Thank you daddy. You said FGMC is cultural.

Respondent: Yes. It our traditional way of sanitising the community.

Interviewer: please can you tell me more

Respondent: You know if the girl circumcision is done, it will prevent the waywardness of our girls from the home to the community at large thereby promoting decency.

Interviewer: Thank you sir. It is up to how many years that you been doing circumcision

Respondent: Up to 35 years

Interviewer: Thank you sir. What advice will you give the government concerning those that are still doing it? You know you have been doing this job for about 35 years, what do you think the government can do to stop female circumcision?

Respondent: Thy will keep saying it in the radio that they should stop doing it.

Interviewer: Okay

Respondent: Majority of those that are doing it do not attend the meeting. Assuming they attend the meeting, we will always tell them.

Interviewer: There is a meeting that they do?

Respondent: Seriously

Interviewer: Okay. Can I have the opportunity of getting another person that I can interview

Respondent: there is nobody around here among those that are we do attend the meeting together. There is none around here because where we do meet is far

Interviewer: Hmm

Respondent: They are not around here

Interviewer: I understand. In fact, even if it is Ede or Osogbo. We don’t mind carrying out our investigation

Respondent: No problem

Interviewer: Because you know in our culture, we are the only one that understand the reason why we are doing it

Respondent: Yes

Interviewer: Before the government suddenly pronounced that it should be stopped. And we see that even in the first place when the government said we should stop it. I don’t think the people will agree with it immediately.

Respondent: Hmm. It is gradually. Some will do it, while some will not do it. Everybody was saying all kinds of things. But later it will become general, such that everyone will stop doing it.

Interviewer: Okay. That is why they stopped it because there is no benefit in it. But then what was it that was making them to do it. Is there no anything that they see?

Respondent: There is nothing

Interviewer: More than they are just doing it. But is there any benefit for that of male

Respondent: Haa. There is. We cannot do without doing that of male

Interviewer: Thank you so much sir.

INTERVIEW TBA 8 P8 A75 PE M

Int: My name is Mrs Fafowora, a PhD student from university of kwa-zulu natal in South Africa. Being one of the TBAs and a Community leader, we hope you will be able to help us shed some light on the issue of FGMC in osun state. Can you tell us your age sir?

Respondent: I am above 75years old

Int: what was the highest level of education you attain in academics?

Respondent: I didn’t exceed primary six (6)

Int: do you mean standard 6 or.....

Respondent: I was admitted in primary school in 1953. Then, my parents were poor, they could hardly provide the necessary fees I needed, so I didn’t go beyond primary six. However, I thank God, today I have achieved a lot and my worth is far more than some university graduate.

Int: we thank God. But do you reside within this community

Respondent: yes, I reside here.

Int: Okay sir. For how long have you stayed in this community?

Respondent: I was born in this area. Although, I am an indigene of Ido-Osun, but we reside in this new site.

Int: Sir, we like to know, how you became one of the TBAs

Respondent: I became a practicing TBA by inheritance therefore, it is important that whatever skills we acquire, one should be diligent in practising it and as well ensure our children also become partaker, else such skill will go into extinction.

Int: Okay sir, since you inherited traditional birthing skills, for how long have you been practicing

Respondent: i have been practicing for a very long time, for over 40 years. However, I start practicing traditional birthing as part time. But when I retired from civil service, i became a fulltime TBA.

Int: For how long you have been into FGM/C business for girls, or did you start the moment you became TBA

Respondent: I started the female circumcision a very long time. It’s now that the government said that we should not do it again. Although, during the time of our fathers, there was a man in Osogbo called “Olola” FGM was his specialization. He inherited the skills to conduct FGM. He applied the water extracted from snail on the wound after cutting any girl. But now the health workers are doing it.

Int: Even at that, you would have worked with those that conduct female circumcision or direct some parents to take their girl child to them for Circumcision.

Respondent: you mean health workers?

Int: Yes

Respondent: very well, severally.

Int: like how many do you refer to them in a month

Respondent: most women today put to bed in hospitals, so they conduct the female circumcision in a safe environment -hospital. If you don’t circumcise a girl it may lead to infertility. The clitoris of a female who is not circumcise is like a male’s penis.

Int: Have you ever heard of any government effort towards eradicating female circumcision

Respondent: yes, there has been various campaign towards eradication female circumcision.

Int: what is your take about the government’s campaign to stop female circumcision?

Respondent: I am not in support of it. I don’t believe it is right to stop female circumcision. What is the exact reason why government want to eradicate the practice? Whatever we inherited should not be abandoned, we inherited the female circumcision from our fathers. We should not rate another people’s culture over ours.

Int: thank you sir, what you are telling me is that you are never involved in any of the Government efforts towards eradicating female circumcision

Respondent: I am not involved in any effort towards eradicating female circumcision

Int: It is well documented that there are different types of female circumcision worldwide, based on your experience, can you tell me the type of female circumcision that you do

Respondent: the one I am familiar with, when they open the vagina, then they take out a small part around the clitoris

Int: do they scrape or cut

Respondent: they cut a small part of the clitoris

Int: Can you please tell me about the cultural importance of female circumcision?

Respondent: Culturally, it is believed that any girl that is not circumcised will be promiscuous.

Int: Please shed more light on that sir

Respondent: Most often she would experience some sexual sensation in the clitoris. Any girl with such sensation is easily abused sexually. Some people even make jokes such as, asking why someone behave like a girl that has not been circumcised. The clitoris often become aroused like the penis and the uncircumcised girl want to be with men most time. Let me explain it to you. The clitoris, when left uncut, grows like the penis. It is very sensitive to any touch. That is why you hear of some girls masturbating. It is because they are easily sexually excited which eventually leads to promiscuity.

Int: is that the only reason for female circumcision

Respondent: yes, that is the only reason

Int: Do female circumcision benefit the community in any way

Respondent: when we circumcise our girl child, firstly parent’s mind will be at rest if such girl leaves the home. They will be at peace in the future.

Int: does religion has anything to do with female circumcision?

Respondent: I am a Muslim. It is important to circumcise our girl child once she is born. We believe that the little aspect of the clitoris that is cut is dirt to the female genital. So, it must be removed.

Int: Based your experience as a TBA, what can you tell me about stopping female circumcision

Respondent: Based on my experience, our people will never concede to them on eradication of female circumcision. That is why it is been practice secretly. Since no one can know or decide to see our children’s vagina. I am in the best position to take care of my children, No one should decide for me.

Int: lets assume we are privilege to stop FGM since there are some that are …

Respondent: you remember i said we should expose our children to whatever skills, we inherited and practicing. Unfortunately, education and looking for white collar job has caused most of our descendants to abandon the practice leaving people at the mercy of the quacks. The family of Oloola inherited female circumcision from their fathers. There is a saying that, “no child dies while being circumcised by Oloola”. The fact that so many quack Circumciser are into the practice recently, is the reason so many complications are being experienced. If we allow the Oloola to be the only group doing it, we will not be experiencing those complications

Int: the government in recent time organised so many programmes that was designed to eradicate FGM, what do you see to such programmes?

Respondent: I heard that the government said we should stop female circumcision, but we will never consent to that. We should be allowed to promote our tradition and culture not the westerners’. Let me tell you that nobody will come out and announce to the government that I have circumcised my girl child.

Int: Since you are a community leader, what are your people saying about FGMC

Respondent: The people in the community are of the opinion that female circumcision should continue so that our girl children will not be promiscuous. There was a time that Ghanaian flooded Nigeria because of the crisis in their country. It was observed that the females among them were very promiscuous because they were not circumcised. In addition, a husband whose wife is not circumcised will not enjoy the wife sexually.

Int: How will you advise the government towards successful elimination of FGM

Respondent: you mean other thing we can continue to do. A saying goes thus, “as bird flies, we throw the stone”: “experience gives birth to knowledge”. One must ensure we make sustainable plan, the future matters most.

Int: what do you mean by that sir?

Respondent: the right people should be involved in the programmes designed to eliminate female circumcision. The government has been wasting resources on the wrong set of people who are claiming to be TBAs. Has it stopped people from performing female circumcision? The experience of the government from such programmes should push them to a better approach. Involvement of the real TBA is important if the government want to succeed.

Int: You mentioned that the TBA who inherited female circumcision should be the only one to do it. Is that your advice to the government so we don’t experience complication anymore?

Respondent: I will only say, only those who inherited the practice should do it some are still alive, some are in Osogbo, some are in Ede. They should allow them to do it. Whenever they circumcise, they should not be careless. After doing it, they should expose the girl child to some heat. It will pain her but the mother should back her and apply Vaseline, it will heal up in time.

TBA 3 Muslim Male Secondary 71 years 60 years

abdulahi

TBA 3a

Respondent: I had to find a way turning people back because of the incessant embarrassment from the government agencies.

Interviewer: Sir, you mean you stopped doing it because of the fear of the government?

Respondent: Yes, because I must save myself

Interviewer: but you told me earlier that circumcision is inherited in your family and must continue

Respondent: my child, you are very correct, but I will not allow those that does not have insight into our work to smear me.

Interviewer: Thank you for the response. Sir, it means that you still have a way of conducting female circumcision.

Respondent: if you want a child to survive in the mother’s womb, you cannot take away the placenta. Is that not it?

Interviewer: you mean that female circumcision cannot be taking away if I get you right sir?

Respondent: of course! The government is only trying but it cannot be taken away because no one is advertising it. Thank God it is gradually ending especially for the fake ones who are perpetrating the evil act calling themselves circumcisers.

Interviewer: thank you sir. Did you say the “fake ones”?

Respondent: Yes, they are the ones that the government is using to attack the real circumcisers because they do not know what we cut when we do circumcision.

Interviewer: if that is the case sir, I want to ask that what are the factors that you think can make it difficult to eliminate female circumcision?

Respondent: there are many things that are affecting the abandonment of the female circumcision. It is beyond what the government can just decide that it must be stopped.

Interviewer: thank you sir but I want you to tell me more

Respondent: you see, female circumcision is a cultural identity. You will discover that it is not done in some other areas.

Interviewer: yes sir

Respondent: it is the identification of who we are as a people. It is our culture! The people in the government are products of this same cultural belief. So, whatever they are doing is just to please their colonial master. You and I know that our culture still belongs to us.

But the children of nowadays have disregarded the practice because of civilization.

Interviewer: thank you sir for your response.

Respondent: another issue that could prevent the abandonment is unavailability of job opportunities. I hope you are taking note.

Interviewer: Yes sir.

Respondent: like I told you earlier on, the original TBAs have their various jobs. we do not rely on whatever proceeds that come out of the procedure. In fact, it cannot even feed us. But those that are carrying out the procedure because they do not have job want to remain in the business to feed themselves. They must make the circumcision looks as if they are doing a big thing cutting what they are not supposed to cut to collect money from their victims. Many cases that need repair are brought to me after mistakes have been made.

Interviewer: that is serious!

Respondent: Parents do not want to raise female children that will soil the name of the family by sleeping with men around. So, most parent will prefer to cut just the tip of the clitoris to prevent future regrets.

Interviewer: Sir, I was informed that cutting the clitoris does not prevent girls from sleeping around with men.

Respondent: you may be right but don’t forget that the female clitoris if the equivalent of male penis. This clitoris is the most sensitive part of the female sexual organ. If left uncut, the clitoris grows and mere touching it even by the girl can cause sexual excitement.

Interviewer: okay

Respondent: you want to know more?

Interviewer: Yes sir

Respondent: if the clitoris is left uncut as I have mentioned earlier, it looks like folded skin and if not properly taken care of, it can habour microorganisms that can cause infections.

Interviewer: thank you so very much sir for your time. Please sir, I have some other questions to ask.

Respondent: Please go on

Interviewer: we have talked about those factors that can make the abandonment of female circumcision difficult. Please sir, can you share with me those factors that can help in ensuring that the female circumcision is stopped.

Respondent: ha! Form me?

Interviewer: Yes sir.

Respondent: first, the government needs to be decisive and clamp down on those circumcisers who are using female circumcision to defraud people. All I see the government doing is a kangaroo way of dealing with the issues. The right people must be contacted first, just like you are doing now. We have an association which will help the government to get more insight into the female circumcision. And will be able to identify where the problem comes from.

Interviewer: okay sir

Respondent: all these circumcisers that are causing harm to our girls in the name of female circumcision because of making ends meet can be provided with jobs or better still empowered.

Interviewer: thank you sir

Respondent: But the government should intensify the publicity on radio, although they were doing it before but stopped. They should continue to announce it in the marketplace and if possible, print small papers like the believers’ tract so that they can distribute it to the people. Anyone who can take it home and his/her child will read it to him/her at home.

Interviewer: Thank you sir.

Respondent: the difference between what we do and what the other group does should be made known to the public and not generalize at all.

Interviewer: Thank you so very much sir. Do you know anyone who does female circumcision? You may not mention the name just the address so that I can go there for this kind of research I am doing here?

Respondent: The person that does the circumcision also has a signboard. When you get to Gbelenkan area. He has a clinic. The place is not far from the New Market area. He has a signboard there. What I am saying is what you will hear from him also.

Interviewer: You know when we are doing research like this, we like to speak to different people. What you will say may be different from what he will say. He may say some things that may be different from what you are saying now.

Respondent: Yes, he was working in Lagos before but when he retired, he came back home to start the work.

Interviewer: When we are doing research like this, we like to speak to different people, sometime, we speak to about eight people. For instance, now, what you will say may be different from what he will say. There are things you will say that he will not say, there are also things he will say but you may not say and when both are saying the same thing then we know that. This will make the research full.

Respondent: it is good. Your parent would have done it for you.

Interviewer: Yes, they said that. My friend that I am about four years older than her said it was done for her. There is one that we are about the same age, she said it was done for her too.

Respondent: They did it for you because as at that time the law prohibiting it has not been made.

Interviewer: Are you saying that female circumcision is not done again because of the law?

Respondent: People are afraid of the government agencies that are enforcing the law. It is done in secrecy and out of fear. If the government can be consistent in enforcing the law in the right direction, sanity will return in terms of female circumcision.

Interviewer: Thank you sir. I hope you will oblige me anytime I come for further clarification.

Respondent: You are always welcome.

TBA interview

P10 A67 SE Amoo Akinyemi David Christian

Interviewer: What is your thought about circumcising a female child? Is good to circumcise a female child?

Respondent: To my own understanding it is good to circumcise a female child because the people in the government that are saying it should be stopped are circumcising their female children, but they will not come out and tell you! I just see it as personal decision. Because of the sensitivity of the clitoris, female circumcision prevents early sexual debut thereby preventing diseases like cancer of the cervix. Am I right?

Interviewer: You are right sir

Respondent: So, for female child to be preserved the best thing is to circumcise them. Female circumcision in this part is also done for esthetic reason. A clitoris left uncut grows like a penis which may make the private part to be irritating to the future husband. But if the tip is cut, the clitoris will not be too long, and unnecessary sexual excitement will be controlled.

Interviewer: thank you sir. Someone told me also that female circumcision is very good, but it should not be cut too deep. What do you have to say to that?

Respondent: The person is right. Cutting too deep will not let the woman enjoy sex because it is the clitoris that makes woman to enjoy sex. That is what makes the difference between the true circumcisers and those that are just cashing in on it.

Interviewer: Government is announcing that they should stop circumcising female child where did you hear it

Respondent: I heard it from radio

Interviewer: What do they say on radio?

Respondent: They said circumcising female child is not good it does not let them to enjoy sex and if they cut the thing and there is bleeding it may cause the child’s death and then it can cause infection because they won’t sterilize the instrument they will use they will just use any blade which is not new from local place all this may cause infection that is why government said we should stop doing it

Interviewer: Is it good to circumcise a female child and not to stop it at all

Respondent: In my own view there is a woman here she has a female child which is 10years and her clitoris is long and is becoming big in her body and I told her that I cannot help her to cut it she is afraid now that if she wants to give birth and the head of the child touch the clitoris.

Interviewer: But can we use hot cloth to be massaging the clitoris when a female child is born so as not to let that clitoris grow.

Respondent: I don’t know about that one, but maybe because the skin is still soft before it gets strong, it will not be able to grow again.

Respondent: Men will not like it when the penis becomes two if the man arouses the clitoris too will be aroused. Such female child will be ashamed of herself when she is with her friends because her private part will be different.

Interviewer: You have said the reason why government said we should stop circumcising

Respondent: Because of infection and for the husband to enjoy sex with them

Interviewer: Can you advice someone to do circumcision for a female child?

Respondent: Though it is good to circumcise a female child, but it is the parent’s choice. I have told you this earlier on. Although people have moved on, only those who appreciate the advantages of female circumcision give it a thought. It’s just like the extended family system that we were practicing in those day but because people are more enlightened or let me say more educated, it has been abandoned. The same thing is happening to female circumcision.

Interviewer: You mentioned that you do not support the government putting an end to female circumcision

Respondent: what is the essence of government stopping it? The government and its agencies enforced it that female circumcision should be stopped but people are still doing it!

Interviewer: Thank you sir. Let us assume that you support the abandonment of female circumcision, what are the factors that you feel can hinder the government effort to stop female circumcision?

Respondent: It is important to let you know the reason why female circumcision is still being done. There is a strong belief in the culture of female circumcision in Yorubaland. So, people still find their way to the traditional circumcisers and not those who diversified. You will notice that noise about female circumcision has reduced, it is because those performing the dastard acts have greatly been curbed. They do not know what we cut and how we cut it. That is why there are complications here and there. A traditional circumciser will just cut a tip, very small tip of the clitoris and nothing more.

Interviewer: Thank you sir for the explanation. Are you saying that there are different people that perform the female circumcision?

Respondent: Yes, I have explained this to you earlier. It even includes the health workers.

Interviewer: alright sir

Respondent: As we were discussing, my lineage belongs to the traditional circumcisers and the female circumcision is part of what I inherited therefore since it was handed over to me by my fathers, I cannot allow it to just perish like that while I’m still alive, never!

Interviewer: what you are telling me is that you inherited the practice from your forefathers, and you are not backing out.

Respondent: You are right

Interviewer: can you please share other factors that you think can negatively affect the elimination of female circumcision?

Respondent: Yes, people now realized that we are not the ones perpetrating the act of causing complications in female circumcision. That there is a difference between what we do as traditional circumcisers and what other impostors do.

Interviewer: if I get you right sir, you mean people understand your work more than how it is described.

Respondent: Yes. Because we understand what is to be cut and what not to cut. There is limitation to what we cut when performing female circumcision that will not be dangerous for the baby.

Interviewer: Thank you sir.

Respondent: Religion also play a role in ensuring that female children are circumcised.

Interviewer: Can you be specific sir?

Respondent: In Islamic religion, it is believed that the small tissue that is cut from the clitoris is like dirt that must be removed from the girl child private part.

Interviewer: Thank you sir.

Respondent: It is also scary the way our female children are showcasing their body all over thereby promoting promiscuity. No parent would want his or her female child to bring disgrace to the family through these unwholesome behaviours.

Interviewer: Thank you sir. Please, help me to explain further.

Respondent: Don’t you see what is happening all over? Don’t you see the open disgrace where illicit sexual acts are performed openly among our children on daily basis? No self-control again! There is gross sexual misconduct because the government says we should not circumcise the female children again. So, parents who cherish their names and do not want regrets in the future will circumcise their female children.

Interviewer: Thank you sir

Respondent: That is why strange diseases are all over the place. What should be enjoyed naturally becomes a menace to the society. Look, it saddens my heart, because little do the government and its agencies know that they are doing a great disservice to our children. Imagine the insanity in the white people’s society that our government is collaborating with? May God have mercy on us.

Interviewer: Amen sir. Thank you so much sir for the opportunity you have given me. I hope you will still give me the chance if I need to consult you again sir.

Respondent: my doors are always open just let me know ahead.
