# Supplementary material for: ‘It is what was handed over to us as our heritage and must not be taken away just like that’: Traditional birth attendants’ attitudes towards the elimination of intergenerational female genital mutilation/cutting in Osun State, Nigeria
Source: PLOS Glob Public Health. 2024 May 20;4(5):e0001585. doi: 10.1371/journal.pgph.0001585 (PMC11104658; doi:10.1371/journal.pgph.0001585)
Supplement: Attachment — Submitted filename: comments.pdf [file pgph.0001585.s005.pdf]

PLOS Global Public Health

'It Is What Was Handed Over to Us as Our Heritage and Must Not Be Taken Away Just Like That': Traditional Birth Attendants' Attitudes Towards the Elimination of Intergenerational Female Genital Mutilation/Cutting in Osun State

--Manuscript Draft--

**Manuscript Number:**	PGPH-D-22-01175R3
**Article Type:**	Research Article
**Full Title:**	'It Is What Was Handed Over to Us as Our Heritage and Must Not Be Taken Away Just Like That': Traditional Birth Attendants' Attitudes Towards the Elimination of Intergenerational Female Genital Mutilation/Cutting in Osun State
**Short Title:**	Eliminating Intergenerational Female Genital Mutilation/Cutting in Osun State, Nigeria
**Corresponding Author:**	Rosemary Omolara Fafowora, MSc. in Nursing<br>University of KwaZulu-Natal - Howard College Campus<br>Durban, SOUTH AFRICA
**Order of Authors:**	Rosemary Omolara Fafowora, MSc. in Nursing
	Sinegugu Evidence Duma, RN., Ph.D
**Keywords:**	Key Words: Replace Community Readiness Tool, Intergenerational Female Genital Mutilation/Cutting, Traditional Birth Attendant
**Abstract:**	Abstract<br>Background<br>Female genital mutilation/cutting is a harmful practice that violates the sexual and reproductive health rights of women and girls. The practice is often perpetrated directly or indirectly from one generation to another as a way of preserving culture, thus making it difficult to tackle using ordinary prevention interventions. The purpose of the study was to assess the attitude of the traditional birth attendants as community leaders towards the elimination of intergenerational female genital mutilation/cutting and to determine their level of readiness and preparedness towards achieving it in Osun State, Nigeria.<br>Methodology/Results<br>A qualitative research design, using the adapted REPLACE community readiness tool to end female genital mutilation/cutting interview guide, was used to individually interview eight traditional birth attendants who were identified through purposive sampling method as community leaders and key informants. Thematic Analysis was used to analyze the data which yielded female genital mutilation/cutting as traditional heritage, defiance against government efforts and debunking all “western” information about dangers of female genital mutilation to women as lies as findings. These findings are worrisome because they are an indication of the community leaders’ level of no readiness for interventions for elimination of intergenerational female genital mutilation/cutting in Osun state, Nigeria.<br>Conclusion<br>We recommend that the government, policy makers and health professionals elicit and carefully consider the views of the community leaders such as traditional birth attendants who are custodians of the female genital mutilation/cutting before introducing measures to eliminate it in the traditional communities. This may include having non-confrontational dialogue between all stakeholders, for understanding of the dangers and benefits of female genital mutilation/cutting to the nation which can be shared from everyone for best solutions to be found.<br>Key Words: Replace Community Readiness Tool, Intergenerational Female Genital Mutilation/Cutting, Traditional Birth Attendant
**Additional Information:**	
**Question**	**Response**
**Financial Disclosure**	The author(s) received no specific funding for this work.

Powered by Editorial Manager® and ProduXion Manager® from Aries Systems Corporation

Enter a financial disclosure statement that describes the sources of funding for the work included in this submission and the role the funder(s) played. This includes grants and any commercial funding of the work or authors.<br><br>This statement will be typeset if the manuscript is accepted for publication.<br><br>*Please review the submission guidelines and the instructions link below for detailed requirements and guidance.*	
**Competing Interests**<br><br>On behalf of all authors, disclose any competing interests that could be perceived to bias this work.<br><br>This statement will be typeset if the manuscript is accepted for publication.<br><br>*Please review the instructions link below and PLOS Global Public Health's competing interests policy to determine what information must be disclosed at submission.*	The authors have declared that no competing interests exist.
**Data Availability**<br><br>Before publication, Authors are required to make fully available and without restriction all data underlying their findings. Please see our PLOS Data Policy page for detailed information on this policy.<br><br>A **Data Availability Statement**, detailing where the data can be accessed, is required at first submission. Insert your Data Availability Statement in the box below.	All relevant data are within the paper and its supporting information file.

Please see the data reporting section of our submission guidelines for instructions on what you need to include in your Data Availability Statement.<br><br>This statement will be typeset if the manuscript is accepted for publication.<br><br>*PLOS allows rare exemptions to address legal and ethical concerns. If you have legal or ethical restrictions, please use the box below to detail these in full sentences for the Journal team to consider.*	

Clean copy
Click here to access/download;Manuscript;Clean copy.docx

Full Title:

‘It Is What Was Handed Over to Us as Our Heritage and Must Not Be Taken Away Just Like That’: Traditional Birth Attendants’ Attitudes Towards the Elimination of Intergenerational Female Genital Mutilation/Cutting in Osun State, Nigeria.

Short Title:

Eliminating Intergenerational Female Genital Mutilation/Cutting in Osun State, Nigeria.

Authors:

ROSEMARY OMOLARA FAFOWORA[1¶*ID] orcid.org/0000-0003-2968-3784

SINEGUGU EVIDENCE DUMA[2¶ID] orcid.org/0000-0002-2489-8770

[1]Department of Nursing, School of Nursing and Public Health, College of Health Sciences, Howard Campus, University of KwaZulu-Natal, Durban, South Africa.

[2]Department of Nursing, School of Nursing and Public Health, College of Health Sciences, Howard Campus, University of KwaZulu-Natal, Durban, South Africa.

*Corresponding author

E-mail: rosemary.olagunju@gmail.com (RO)

¶These authors equally contributed to this work.

Abstract

Background

Female genital mutilation/cutting is a harmful practice that violates the sexual and reproductive health rights of women and girls. The practice is often perpetrated directly or indirectly from one generation to another as a way of preserving culture, thus making it difficult to tackle using ordinary prevention interventions. The purpose of the study was to assess the attitude of the traditional birth attendants as community leaders towards the elimination of intergenerational female genital mutilation/cutting and to determine their level of readiness and preparedness towards achieving it in Osun State, Nigeria.

Methodology/Results

A qualitative research design, using the adapted REPLACE community readiness tool to end female genital mutilation/cutting interview guide, was used to individually interview eight traditional birth attendants who were identified through purposive sampling method as community leaders and key informants. Thematic Analysis was used to analyze the data which yielded female genital mutilation/cutting as traditional heritage, defiance against government efforts and debunking all “western” information about dangers of female genital mutilation to women as lies as findings. These findings are worrisome because they are an indication of the community leaders’ level of no readiness for interventions for elimination of intergenerational female genital mutilation/cutting in Osun state, Nigeria.

Conclusion

We recommend that the government, policy makers and health professionals elicit and carefully consider the views of the community leaders such as traditional birth attendants who are custodians of the female genital mutilation/cutting before introducing measures to

eliminate it in the traditional communities. This may include having non-confrontational dialogue between all stakeholders, for understanding of the dangers and benefits of female genital mutilation/cutting to the nation which can be shared from everyone for best solutions to be found.

Key Words: Replace Community Readiness Tool, Intergenerational Female Genital Mutilation/Cutting, Traditional Birth Attendant

Introduction

Female genital mutilation/cutting (FGM/C) is a public health problem globally. It comprises all procedures that involve partial or total removal of the external female genitalia, or other injury to the female genital organs for non-medical reasons. It is also referred to as multiple procedures on the female genital organs for non-medical reasons [1]. FGM/C has been described as a harmful practice that violates the sexual and reproductive health rights of women and girls [2,3]. It is estimated that more than 200 million girls and women alive today have undergone FGM/C across 30 countries including the Middle East, Asia, and Africa [4]. Of this, about 44 million are girls under the age of 15 years [5]. It is further projected that more than 3 million girls and women are currently at risk of FGM/C worldwide [4]. The current estimates of 3.9 million girls mutilated annually will also rise to 4.6 million by 2030 because there is an increase in the population growth in the communities that still practice FGM/C [6]. It is reported that 20 million girls and women are estimated to have undergone FGM/C in Nigeria, with Osun state, where the study was conducted, having reached the highest prevalence of 76.6% of FGM/C [7].

In Nigeria, FGM/C is a deeply entrenched custom that is commonly performed by the traditional birth attendants (TBAs) who claim it as a heritage which confers honor on a woman and her family, a rite of passage to womanhood, preservation of chastity, enhancement of fertility, and prevention of death of the child during delivery among its benefits [8].

Despite its cultural importance, FGM/C has received considerable criticism worldwide because of its potential risk for various forms of health complications [9]. The short term and physical health complication of FGM/C includes but are not limited to pain, risk of hemorrhage, shock, tetanus/sepsis, inability to urinate, damage to adjacent organs such as the rectum and urinary tract, dislocation and fracture of the pelvic bones and the head of the femur due to struggle while being restrained, open sore in the genital region, risk of HIV/AIDS or Hepatitis B. While the long term and reproductive health consequences of FGM/C include difficulty in passing menstrual blood, vaginal fistula, painful sexual intercourse, and difficulty in passing urine which can lead to recurrent urinary tract infection, clitorial cyst, infertility, an increased risk of childbirth complication and newborn death, need for future surgeries to allow for sexual intercourse and childbirth, psychological trauma, and risk of death from hemorrhage or infection [10].

Furthermore, sexuality is an equal right that both males and females must benefit from. While men are being circumcised for health reasons, women and girls are cut deliberately to remove their sexual feelings and subjugate them to the dictates of their men counterpart and it is done without any consent from them.

In 2005, the Violence Against Persons (Prohibition) Act 2015 was passed to eliminate the FGM/C practice in Nigeria. Since then, several legal, community based, and health-based strategies had been instituted to eliminate the FGM/C practice [11]. These strategies were met

with strong opposition from the TBAs, who remain the self-proclaimed custodians of the traditional responsibility of FGM/C from time immemorial, especially in Osun State, Nigeria. The TBAs believe in the supremacy of FGM/C as the traditional heritage, which is concerned about the wellbeing of the people, that it predates modern medicine and therefore should not be stopped. This cultural belief has facilitated the intergenerational preservation of FGM/C and explains their attitude towards the elimination of its practice. The intergenerational preservation of FGM/C is reflected on most women's belief that the elimination of FGM/C is tantamount to defaulting from their religion or traditions which could breed calamity for them and their offspring. These beliefs have been passed down from one generation to the other and contribute to the high prevalence of FGM/C in Osun State, Nigeria, where it has become major public health issue that requires public health approach-based prevention interventions. Because of their influence on the intergenerational preservation of FGM/C, we identified the TBAs as community leaders who are key towards the development and implementation of public health prevention intervention for FGM/C in Osun State, Nigeria. The purpose of the study was to assess the attitude of the TBAs as community leaders towards the elimination of intergenerational FGM/C of girls in Osun State, Nigeria, in order to determine their level of readiness and preparedness towards achieving its elimination.

Materials and Methods

Study setting

Osun State is an inland state in South-West Nigeria with a population of 3,423,535 [12]. The present Osun State was created in 1991 from the old Oyo State. The state shares boundary with Kwara State in the North, Oyo in the West, Ogun and Ondo in the South and Ekiti State on East. The state capital is Osogbo, which is a local government area on its own. In all, a total of thirty local government council areas and Ife east area council constitute the state. Among the major towns in the state are Ilesha, Ife, Ede, Ikirun, Ikire, Ejigbo and Osogbo

[13]. Before this time, the state was part of the old Oyo State. Osun State experiences a tropical climate while the local vegetation is the lowland rainforest type.

Indigenes of Osun State belong to the Yoruba race and comprise the Oyo, Ife, Ijesa, Igbomina and Osun. Many non-indigenes and foreigners also reside in the state and live together in harmony. Yoruba and English are the languages used for official and business transactions. The people have a rich cultural heritage that is demonstrated in all spheres of their life including the performance of FGM/C. Their culture finds expression in their arts, literature, music, and other social activities.

This study was conducted among the Yoruba TBAs residing across the three senatorial districts in Osun State, Nigeria. The Yoruba people have a rich cultural heritage that is demonstrated in all spheres of their life including the performance of FGM/C in the Osun State, Nigeria.

Research design

A qualitative research design, using the adapted REPLACE community readiness to end FGM/C interview guide [14] was used to assess the attitudes of the TBAs as community leaders towards elimination of intergenerational FGM/C to determine their level of readiness and preparedness towards achieving its elimination in Osun State, Nigeria. This was the most suitable approach because it focuses on social and cultural contexts to understand how a problem is perceived and whether taking some type of action is seen as needed by community leaders to change the problem [15, 16]. The adapted REPLACE community readiness to end FGM/C interview guide [14] was translated to the local (Yoruba) language by a bilingual expert in Yoruba and English who is also a qualitative researcher for comparison of the language translation to avoid the loss of meaning in the translated interview guides. The same person was used for translating transcripts to English.

Sampling and recruitment of participants

Purposive sampling method was used select eight TBAs whom we were regarded as community leaders and key informants with rich information regarding intergenerational FGM/C in Osun State, Nigeria. The inclusion criteria for recruitment and participation in the study included only TBAs who were formally recognized as leaders in performing FGM/C, who had been previously involved in any of the Government's organized seminars or workshops on FGM/C elimination and or prevention intervention in Osun State, irrespective of the duration of the practice. The selection and recruitment of the participants commenced once the ethical approval from the Humanities and Social Sciences Research Ethics Committee [HSSREC/00000924/2019] and Osun state Ministry of Health [OSHREC/PRS/569T/157] was obtained. Meetings were held with the nurses in the maternity units across the three senatorial districts as gatekeepers to inform them about the purpose of the study and to seek their permission to meet the TBAs working in their respective Units. A meeting with TBAs was held where the purpose of the study, the research eligibility criteria and request for voluntary participation from those who meet the inclusion criteria were discussed. Those who showed interest were recruited and were given the information sheet and the informed consent form to complete and sign. Arrangements for the interview session were then made by allowing the potential participants to choose suitable date and time within the first 7 days after the participants have given their consent. Before the commencement of the interview, the purpose of the study, and permission to audio record the interview session were explained again to the participants in the local language (Yoruba). A total sample size of eight traditional birth attendants as community leaders and key informants were recruited in the study. This small sample size is in line with the REPLACE community readiness tool, which suggests 6-12 key informants as sufficient to address issues in the community [14].

Pilot study

The first two TBAs out of the sample size were used for pilot study to ensure that the interview guide questions were easy and yielded the necessary data. The data generated from the pilot study were included in the main study data analysis. Contamination of data was not considered to be a concern because in qualitative studies data collection is progressive and the data from pilot studies can be used for the main study [17,18].

Data collection

Data collection and data analysis were conducted concurrently between October 2020 and March 2021. All COVID-19 precaution protocols like using the face mask, maintaining social distancing and hand sanitizing were observed during the interviews. In-depth interviews were conducted with the individual TBAs in their homes by the first author in Yoruba, the local language, using the interview guide adapted from the REPLACE Community Readiness to End FGM/C Assessment tool [14]. The interviews were audio recorded and transcribed within 24 hours of recording when information was still fresh in the researcher's memory for data analysis purposes. Each interview lasted about 60 minutes to complete thus allowing each TBA to adequately share their views as attitudes towards elimination of intergenerational FGM/C in Osun state. Nigeria. The transcripts were stored in word document formats on a computer with encrypted password and was backed up to an external hard drive to prevent data loss in line with the University's principles for data storage.

Data analysis

The six steps, namely: familiarization, coding, searching for themes, reviewing themes, defining, and naming themes, and writing up as described by [19] were used to analyze the transcribed data.

The first author went through the transcripts reading and re-reading them to familiarise herself and make sense of the data. Notes were taken and preliminary ideas for codes were marked. In order to start organizing the data into meaningful groups, codes were assigned to briefly describe something interesting that was said by the participants. The first author grouped the newly generated codes with same meaning together to form themes. The second author, who also doubled as the inter coder at this stage, also did the same thing. The generated themes by the first and second authors were scrutinized by both of them to ensure that they were useful and accurate representation of the data by discarding some themes and creating new ones from the transcribed data. The first author then formulated the meaning of each theme so that the data can be understood. At this stage, appropriate naming of themes was made to succinctly interpret the data collected.

Ethical Permission

All ethical principles were adhered to including, consent form for voluntary participation, ethical approval from the Humanities and Social Sciences Research Ethics Committee [HSSREC/00000924/2019] and Osun state Ministry of Health [OSHREC/PRS/569T/157] were obtained.

The names assigned to each of the participants are not their actual names as we are aware of the ethical implication. However, using pseudonyms instead of participants' actual names ensured confidentiality and anonymity.

Measure to ensure trustworthiness

Measures to ensure trustworthiness and rigor of the findings were applied through the criteria of credibility, confirmability, dependability, and transferability [20].

To ensure credibility, comprehensive field notes, noting gesture and other non-verbal ques observed during the interviews were recorded and analyzed. Audit trail of all research processes were recorded. Analysis of the data gathered reflected a neutral interpretation of the data rather than the researcher's view as recommended by [21,22]. This was achieved by conducting a member checking on data whereby the analyzed data was summarized and provided to the participants for them to validate and determine the accuracy of the data as their views. For dependability and transferability, an audit trail was kept and the comprehensive and rich details of the study setting, and the socio-demographic characteristics of the participants are presented. To further establish trust and confidence in the findings, the authors compared their individually generated themes from the raw data to ensure that they were useful and accurate representation of the data by discarding some themes and creating new ones from the transcribed data. The senior author and research supervisor reviewed the themes against raw data and helped in refining these to reflect participants' views to enhance confirmability. Common issues recur and main themes were identified to summarize all the views that have been captured in the data. One theme and three subthemes emerged that revealed the defensive attitude of the TBAs towards the elimination of intergenerational FGM/C of girls in Osun state, Nigeria.

Results

Eight (8) TBAs, comprising of three females and five males participated in the study on TBA's attitude towards the elimination of intergenerational FGM/C of girls in Osun State, Nigeria.

Table 1: Participants' Demographic Characteristics

Participant	Age	Gender	Religion	Educational level
Lukman	45	Male	Islam	Tertiary education
Abike	70	Female	Islam	Primary education
Adeyemi	53	Male	Islam	Tertiary education
Olalekan	86	Male	Islam	Primary education
Mojirola	63	Female	Christian	Secondary education
Amope	80	Female	Islam	Primary education
Fadipe	73	Male	Islam	Secondary education
Adebayo	75	Male	Islam	Primary education

These demographics reflects the TBAs in Osun State where the person who becomes a TBA is culturally determined through the lineage of the traditional circumcisers. It is a taboo for a woman who is married to perform FGM/C as TBA, but she can still retain her knives for performing FGM/C on her own children only (personal conversation with Mr Adeyemi, a male participant to the study, December 2020). This explains why most participants were male TBAs in the study as reflected in Table 1. Their ages ranged between 45 and 86 years. Seven of them practiced Islam and only one was Christian as a religion.

Defensive attitude towards elimination of intergenerational FGM/C

Analyzed data yielded “Defensive attitude towards elimination of FGM/C” as the main theme in the TBAs’ attitude towards the elimination of intergenerational FGM/C of girls in Osun State, Nigeria. Three subthemes, including (i) FGM/C as a traditional heritage, (ii) Defiance against government’s efforts to eliminate intergenerational FGM/C and Debunking all “western” information about dangers of FGM/C as lies emerged under the main theme.

FGM/C as traditional heritage

The FGM/C as a traditional heritage sub theme emerged from data related TBAs’ defensive attitudes towards elimination of intergenerational FGM/C as traditional and cultural beliefs that the practice is the identity and the only link between the current and past among generations as described in the following extracts:

I am talking about our traditional heritage here. It can’t be stopped; it can’t stop now. The law, civilization coupled with ignorance is stopping people, but it cannot be stopped.

[Lukman, Male, Age: 45, Tertiary Education]

I think all they are saying about stopping of our tradition is done out of civilization and hatred, hatred of us and our traditional heritage. What has been established by our forefathers cannot perish. It is just like asking us to abandon our language because of civilization. There is no where we meet that we can do without speaking our language. Girl child circumcision cannot perish. [Abike, Female, Age:70, Primary Education]

Further data under this subtheme showed that TBAs consider it as their primary responsibility to protect intergenerational FGM/C practice from extinction as demonstrated in the following extracts:

Girl circumcision has been in existence from time immemorial. Therefore, it can be described as our cultural identity that was transmitted from generation to generation that no one is ready to allow it to die down in his or her own time, even if your government says it is bad for us. [Fadipe, Male, Age:73, Secondary Education]

But you know, it is our heritage which must be promoted. It is not that it really makes more money for us. Do you understand me? It is not what the TBAs are eating from. But it is what was handed over to us as our heritage and must not be taken away just like that! [Abike, Female, Age: 70, Primary Education].

Defiance against government's efforts to eliminate FGM/C

This subtheme emerged from data related to the defiant attitudes of the TBAs towards seminars and workshops that had been previously arranged by government and other organizations in the country with the aim of highlighting the disadvantages of intergenerational FGM/C proposals towards eliminating FGM/C as described in the following extracts:

I attended the workshops about this, but I was and am still convinced that the disadvantages of government's effort to stopping FGM/C will be enormous for our women. See what is happening nowadays in our communities. No one will be able to control these girls. We must fight anyone including the government to protect our girls from promiscuity. [Lukman, Male, Age: 45, Tertiary Education]

No, I don't agree with the government's decision. The result will be very bad. As for me, I did it for all my children both boys and girls. None of my children are promiscuous because I circumcised them and took care of them all. I buried what was cut and properly watered the soil where I buried it so that chicken could not be able to unearth it. Otherwise, if unearthed, the girl child will be promiscuous. We are very careful about these things so that no one can come back and say FGM/C doesn't work. [Amope, Female, Age: 80, Primary Education].

Ah! I am not in support of the government stopping the girl child circumcision. That is our campaign, and I am not hiding it. At least, it will not happen in my time. [Abike, Female, Age 70, Primary Education]

Personally, I don't agree with government's reasons to stop girl child circumcision. I have said it earlier that, based on my experience and the level of my education, and coming from a family that is traditionally practicing FGM/C, I do not see it being stopped by anyone, even the government or anyone else, not now. I did it (FGM/C) for my girls and there is no problem associated with it, I don't think it should be stopped. It will be a great disadvantage if anyone could agree with government. [Lukman, Male, Age: 45, Tertiary Education].

Debunking all "Western" information about dangers of FGM/C to women as lies

This subtheme emerged from data related to TBAs efforts in debunking most of the information and claims about the benefits of eliminating intergenerational FGM/C as just government's efforts to brainwash people, against the practice of FGM/C as depicted in the quotes below.

The girls that I circumcised did not have any problem. Even when grown up, these girls do not develop any of the problems we were told resulted from FGM/C, which we were now told can be associated with FGM/C. The girls are now big women and are all alive and doing well in their families. They got married to nice men and have lively children. [Amope, Female, Age 80, Primary Education]

Every girl that we have circumcised is still alive even today. No child can die in the hand of circumcision surgeon and all our girls were circumcised. Anyone who say we put our girls in danger is a liar. My wife is circumcised, all my girls are circumcised and alive. They must stop spreading lies about FGM/C. [Olalekan, Male, Age 86, Primary Education]

The likelihood of lack of enjoyment during sex was further debunked but rather held that circumcised women enjoy sex more as demonstrated in the extract below.

That debunked FGM/C leads to women not enjoying sex is one of the lies the government is peddling about girl circumcision. But they have forgotten that it's only a small part of the clitoris that is cut and not the whole clitoris. it is not true at all. [Adeyemi, Male, Age 53, Tertiary Education]

It is only a false assumption that circumcised women do not enjoy sex. When a circumcised woman has sexual intercourse with men, she will glue to him as if they tie penis and vagina

together, in that way, they both enjoy each other nicely [Olalekan, Male Age: 86, Primary Education]

Furthermore, the views that FGM/C spreads sexually transmitted infections has been refuted by the TBAs as just rumors as captured in the following extracts:

There is nothing like transmission of sexual diseases by FGMC, bleeding and all so many other things that are being said about female circumcision. It is safe and cultural to do it (circumcision). [Mojirola, Female, Age 63, Secondary Education]

We were also told that female circumcision causes sexual transmitted diseases / infections which is not true at all! Honestly, I don't know what the government want to gain from spreading such lies for stopping it. Female circumcision does not have any negative side effects as it is being peddled around. [Amope, Female, Age 80, Primary Education].

Discussion

The purpose of the study was to assess TBAs' attitudes towards the elimination of intergenerational FGM/C of girls in Osun state, Nigeria in order to determine their level of readiness and preparedness towards achieving its elimination.

Our findings show a deep defensive attitude towards elimination of FGM/C among the TBAs as community leaders and custodians of the intergenerational FGM/C which is reflected in the participants views that FGM/C is their traditional heritage and its elimination can be equated to eliminating their cultural identity. Dunn concluded that the cultural identity of the Africans is being eliminated by current civilization ~~[23]~~ and Obi, Adesoye, Labiran also reported that, culture/tradition is the greatest human inheritance, since it contributes to the preservation of history and identity of any race, thus confirming the TBAs' beliefs about their

responsibility to preserve intergenerational FGM/C as their traditional heritage despite all its disadvantages to women, their families, and communities [24].

Findings from this study also depicted a strong defiance against the government's prevention interventions as indicated in the defiance shown by those TBAs who had attended seminars and workshops organised by the government on the elimination of intergenerational FGM/C, thus contrasting previous findings by [25] who concluded that unsuccessful elimination of intergenerational FGM/C was due the government's inability to involve the major community stakeholders such as the TBAs.

Therefore, due to the TBAs' defiance against governments' efforts to eliminate FGM/C, there is a continuous non-alignment between government agencies and the views and beliefs of those responsible for promotion of historic traditions (such as FGM/C) always ending up in disagreement, if not properly handled. Giving the TBAs' voices and understanding their attitudes towards abandoning their age long custom, and not only inviting them to attend seminars or workshops on elimination of FGM/C is important in determining the community's readiness for development and implementation of interventions for eliminating intergenerational FGM/C of girls.

The findings about debunking most of the information and claims about the benefits of eliminating FGM/C as just government's efforts to brainwash people is disputed by previous report which supports the government's assertion on the benefits of eliminating intergenerational FGM/C. Ibrahim, Oyeyemi and Ekine reported that more patients with complications of FGM/C such as difficult delivery, perineal laceration, hemorrhage, scar/keloid etc. were seen by nurses/midwives than doctors in Yenegoa, Bayelsa, Nigeria [26].

The TBAs are of the opinion that the involvement of people who are not traditionally entitled to circumcision and are not knowledgeable enough are the ones causing the complications as stated by the government. The TBAs' debunking of all "Western" information about dangers of FGM/C to women as lies may be due to their lack of knowledge of the dangers and complications of FGM/C. Similar findings were reported by [26] who concluded limited knowledge about complications may be reflected as negative attitudes towards prevention or elimination of intergenerational FGM/C in their lifetime.

Our findings show that FGM/C is going to continue in Osun state, Nigeria despite government's efforts to eliminate intergenerational FGM/C. This is confirming the report by the UNFPA which estimated that because of population growth, additional 4.6 million girls are projected to be at risk of undergoing female genital mutilation by 2030 [27]. It was also reported that the outbreak of COVID-19 between 2020 and 2022 aggravated the vulnerability of girls and women who are at risk of FGM/C which indicated that 2 million cases of FGM/C could be recorded over the next decade due to COVID-19 alone [27].

Conclusion

The current defensive attitudes of the TBAs as community leaders are worrisome because they are an indication of the community leaders' level of no readiness for intervention programmes for the elimination of intergenerational FGM/C in Osun state, Nigeria. We recommend that the government, policy makers and health professionals elicit and carefully consider the views of the community leaders such as TBAs who are custodians of the FGM/C before introducing measures to eliminate FGM/C in traditional communities. This may include having non-confrontational dialogue between all stakeholders for understanding of

the dangers and benefits of FGM/C to the nation which can be shared from everyone for best solutions to be found.

Acknowledgement

Our sincere appreciation goes to the management of the Ministry of Health in Osun state Nigeria and University of KwaZulu-Natal for granting us the ethical approval to carry out the study. We also thank the Nurses and the Traditional Birth Attendants for creating time to grant the interviews.

Data Availability Statement: This is a qualitative study that involves 8 traditional birth attendants who agreed to participate in this study based on anonymity that all information provided will be strictly confidential. All transcripts were anonymized including the extracts that support the study's findings. Transcripts are available for research purposes only in line with the approved research protocol. This is in fulfilment of the participants confidentiality as indicated in the approved research protocol from the Humanities and Social Sciences Research Ethics Committee [HSSREC/00000924/2019] and Osun state Ministry of Health [OSHREC/PRS/569T/157] dated 20th April 2020 and 19th August 2019 respectively.

Funding: The corresponding author received student scholarship from the University of KwaZulu-Natal, South Africa through the College of Health Sciences student Scholarship.

Competing interests: The authors have no competing interests of any form as regards this study.

References

1. World Health Organization. Female Genital Mutilation. Fact sheet. 2020 Retrieved February 20, 2022, from http://www.who.int/mediacentre/factsheets/fs241/en/
2. Waigwa S, Doos L, Bradbury-Jones, Taylor J. "Effectiveness of health education as an intervention designed to prevent female genital mutilation/cutting (FGM/C): a systematic review." Reproductive health 2018; **15**(1): 62.
3. United Nations, 2015. 'The Millennium Development Goals Report 2015', pg. 38. http://www.un.org/millenniumgoals/2015_MDG_Report/pdf/MDG%202015
4. UNICEF. Female Genital Mutilation/Cutting: A Global Concern UNICEF, New York, 2016.
5. World Health Organization. Female genital mutilation: Key facts. 2018 [cited 1 May 2022]. https://www.who.int/news-room/fact-sheets/detail/female-genital-mutilation
6. UNFPA-UNICEF Joint Programme on the Elimination of Female Genital Mutilation (2019).
7. Nigeria Demographic and Health Survey (2013). National Population Commission. Federal Republic of Nigeria.
8. Berg RC, Denison E. Health Care for Women International A Tradition in Transition: Factors Perpetuating and Hindering the Continuance of Female Genital Mutilation / Cutting (FGM / C) Summarized in a Systematic Review A Tradition in Transition: Factors Perpetuating Genital. 2013;9332(October 2017).
9. Yaya, S. and B. Ghose (2018). "Female Genital Mutilation in Nigeria: A Persisting Challenge for Women's Rights." Social Sciences 7(12): 244.
10. Reisel, D. and S. M. Creighton (2015). "Long term health consequences of Female Genital Mutilation (FGM)." Maturitas 80(1): 48-51.

11. Violence against Persons (Prohibition) Act,2015, Laws of the Federation of Nigeria
12. National Population Commission of Nigeria. Nigeria Population and Housing Census 2006. Abuja, Nigeria: National Population Commission of Nigeria.
13. Omole, F.K., Lukman, Y. and Baki, A.I. (2014), "Analysis of market typology and functions in the development of Osun state, Nigeria", International Journal of Development and Sustainability, Vol. 3 No. 1, pp. 55-69
14. Barret H. Alhassan Y. The REPLACE Community Readiness to End FGM Assessment. Practical Handbook (based on REPLACE Approach www.replacefgm2.eu) 2016, Coventry university, UK.
15. McCoy H.V, Malow R., Edwards R.W., Thurland A., Rosenberg R. A Strategy for Improving Community Effectiveness of HIV/AIDS Intervention Design: The Community Readiness Model in the Caribbean, Substance Use & Misuse, 2007; 42:10, 1579-1592, DOI: 10.1080/10826080701212535
16. Borrayo, E. A. (2007). "Using a community readiness model to help overcome breast health disparities among US Latinas." Substance use & misuse **42**(4): 603-619.
17. Duma, S. Women's journey of recovery from sexual assault trauma: a grounded theory.2006.
18. Van Teijlingen, E R, Hundley V. "The importance of pilot studies." (2001).
19. Braun V, Clarke V. Thematic analysis. in H. Cooper, P. M. Camic, D. L. Long, A. T. Panter, D. Rindskopf, & K. J. Sher (Eds.), *APA handbook of research methods in psychology, 2012;Vol. 2. Research designs: Quantitative, qualitative, neuropsychological, and biological* (pp. 57–71). American Psychological Association. https://doi.org/10.1037/13620-004
20. Louis Cohen L, Manion L, Morrison K. Action research. Research methods in education, Routledge 2013: 368-385.

21. Polit, D. and C. Beck. "Essentials of nursing research." Ethics 2012; **23**(2).

22. Fawcett J, Garity J. "Chapter 1: Research and evidence-based nursing practice." Evaluating research for evidence-based nursing: (2009); 3-20.

23. Dunn F. Is It Possible to End Female Circumcision in Africa? Clin J Obstet Gynecol. 2021;1: 007-013. https://doi.org/10.29328/journal.cjog.1001002

24. Obi A.I., Adesoye O., Labiran A. Female Genital Mutilation: Attitude and Practices among Women in Okada Community, Edo State JMBR: A Peer-review Journal of Biomedical Sciences December 2015, Vol. 14 No. 2 pp 138-150

25. Andarge, Masresha Yazew. *The difficulties of ending Female Genital Mutilation (FGM): Case of Afar pastoralist communities in Ethiopia*. *Social Policy for Development (SPD) (2014, December 12).* Retrieved from http://hdl.handle.net/2105/17487

26. Ibrahim I.A, Oyeyemi A.S, Ekine A.A. Knowledge, Attitude and Practice of Female Genital Mutilation among Doctors and Nurses in Bayelsa state, Niger-Delta of Nigeria. International Journal of Medicine and Biomedical Research Volume 2 Issue 1 January – April 2013

27. UNFPA. 2020. 'Impact of the COVID-19 Pandemic on Family Planning and Ending Gender-Based Violence, Female Genital Mutilation and Child Marriage.' Interim Technical Note.

Manuscript

Click here to access/download;Manuscript;Clean manuscript copy.docx

Full Title:

‘It Is What Was Handed Over to Us as Our Heritage and Must Not Be Taken Away Just Like That’: Traditional Birth Attendants’ Attitudes Towards the Elimination of Intergenerational Female Genital Mutilation/Cutting in Osun State, Nigeria.

Short Title:

Eliminating Intergenerational Female Genital Mutilation/Cutting in Osun State, Nigeria.

Authors:

ROSEMARY OMOLARA FAFOWORA[1¶*ID] orcid.org/0000-0003-2968-3784

SINEGUGU EVIDENCE DUMA[2¶ID] orcid.org/0000-0002-2489-8770

[1]Department of Nursing, School of Nursing and Public Health, College of Health Sciences, Howard Campus, University of KwaZulu-Natal, Durban, South Africa.

[2]Department of Nursing, School of Nursing and Public Health, College of Health Sciences, Howard Campus, University of KwaZulu-Natal, Durban, South Africa.

*Corresponding author

E-mail:rosemary.olagunju@gmail.com (RO)

¶These authors equally contributed to this work.

Abstract

Background

Female genital mutilation/cutting is a harmful practice that violates the sexual and reproductive health rights of women and girls. The practice is often perpetrated directly or indirectly from one generation to another as a way of preserving culture, thus making it difficult to tackle using ordinary prevention interventions. The purpose of the study was to assess the attitude of the traditional birth attendants as community leaders towards the elimination of intergenerational female genital mutilation/cutting and to determine their level of readiness and preparedness towards achieving it in Osun State, Nigeria.

Methodology/Results

A qualitative research design, using the adapted REPLACE community readiness tool to end female genital mutilation/cutting interview guide, was used to individually interview eight traditional birth attendants who were identified through purposive sampling method as community leaders and key informants. Thematic Analysis was used to analyze the data which yielded female genital mutilation/cutting as traditional heritage, defiance against government efforts and debunking all "western" information about dangers of female genital mutilation to women as lies as findings. These findings are worrisome because they are an indication of the community leaders' level of no readiness for interventions for elimination of intergenerational female genital mutilation/cutting in Osun state, Nigeria.

Conclusion

We recommend that the government, policy makers and health professionals elicit and carefully consider the views of the community leaders such as traditional birth attendants who are custodians of the female genital mutilation/cutting before introducing measures to

eliminate it in the traditional communities. This may include having non-confrontational dialogue between all stakeholders, for understanding of the dangers and benefits of female genital mutilation/cutting to the nation which can be shared from everyone for best solutions to be found.

Key Words: Replace Community Readiness Tool, Intergenerational Female Genital Mutilation/Cutting, Traditional Birth Attendant

Introduction

Female genital mutilation/cutting (FGM/C) is a public health problem globally. It comprises all procedures that involve partial or total removal of the external female genitalia, or other injury to the female genital organs for non-medical reasons. It is also referred to as multiple procedures on the female genital organs for non-medical reasons [1]. FGM/C has been described as a harmful practice that violates the sexual and reproductive health rights of women and girls [2,3]. It is estimated that more than 200 million girls and women alive today have undergone FGM/C across 30 countries including the Middle East, Asia, and Africa [4]. Of this, about 44 million are girls under the age of 15 years [5]. It is further projected that more than 3 million girls and women are currently at risk of FGM/C worldwide [4]. The current estimates of 3.9 million girls mutilated annually will also rise to 4.6 million by 2030 because there is an increase in the population growth in the communities that still practice FGM/C [6]. It is reported that 20 million girls and women are estimated to have undergone FGM/C in Nigeria, with Osun state, where the study was conducted, having reached the highest prevalence of 76.6% of FGM/C [7].

In Nigeria, FGM/C is a deeply entrenched custom that is commonly performed by the traditional birth attendants (TBAs) who claim it as a heritage which confers honor on a woman and her family, a rite of passage to womanhood, preservation of chastity, enhancement of fertility, and prevention of death of the child during delivery among its benefits [8].

Despite its cultural importance, FGM/C has received considerable criticism worldwide because of its potential risk for various forms of health complications [9]. The short term and physical health complication of FGM/C includes but are not limited to pain, risk of hemorrhage, shock, tetanus/sepsis, inability to urinate, damage to adjacent organs such as the rectum and urinary tract, dislocation and fracture of the pelvic bones and the head of the femur due to struggle while being restrained, open sore in the genital region, risk of HIV/AIDS or Hepatitis B. While the long term and reproductive health consequences of FGM/C include difficulty in passing menstrual blood, vaginal fistula, painful sexual intercourse, and difficulty in passing urine which can lead to recurrent urinary tract infection, clitoral cyst, infertility, an increased risk of childbirth complication and newborn death, need for future surgeries to allow for sexual intercourse and childbirth, psychological trauma, and risk of death from hemorrhage or infection [10].

Furthermore, sexuality is an equal right that both males and females must benefit from. While men are being circumcised for health and religious reasons, women and girls are cut deliberately to remove their sexual feelings and subjugate them to the dictates of their men counterpart, and it is done without any consent from them.

In 2005, the Violence Against Persons (Prohibition) Act 2015 was passed to eliminate the FGM/C practice in Nigeria. Since then, several legal, community based, and health-based strategies had been instituted to eliminate the FGM/C practice [11]. These strategies were met

with strong opposition from the TBAs, who remain the self-proclaimed custodians of the traditional responsibility of FGM/C from time immemorial, especially in Osun State, Nigeria. The TBAs believe in the supremacy of FGM/C as the traditional heritage, which is concerned about the wellbeing of the people, that it predates modern medicine and therefore should not be stopped. This cultural belief has facilitated the intergenerational preservation of FGM/C and explains their attitude towards the elimination of its practice. The intergenerational preservation of FGM/C is reflected on most women's belief that the elimination of FGM/C is tantamount to defaulting from their religion or traditions which could breed calamity for them and their offspring. These beliefs have been passed down from one generation to the other and contribute to the high prevalence of FGM/C in Osun State, Nigeria, where it has become major public health issue that requires public health approach-based prevention interventions. Because of their influence on the intergenerational preservation of FGM/C, we identified the TBAs as community leaders who are key towards the development and implementation of public health prevention intervention for FGM/C in Osun State, Nigeria. The purpose of the study was to assess the attitude of the TBAs as community leaders towards the elimination of intergenerational FGM/C of girls in Osun State, Nigeria, in order to determine their level of readiness and preparedness towards achieving its elimination.

Materials and Methods

Study setting

Osun State is an inland state in South-West Nigeria with a population of 3,423,535 [12]. The present Osun State was created in 1991 from the old Oyo State. The state shares boundary with Kwara State in the North, Oyo in the West, Ogun and Ondo in the South and Ekiti State on East. The state capital is Osogbo, which is a local government area on its own. In all, a total of thirty local government council areas and Ife east area council constitute the state.

Among the major towns in the state are Ilesha, Ife, Ede, Ikirun, Ikire, Ejigbo and Osogbo [13]. Before this time, the state was part of the old Oyo State. Osun State experiences a tropical climate while the local vegetation is the lowland rainforest type.

Indigenes of Osun State belong to the Yoruba race and comprise the Oyo, Ife, Ijesa, Igbomina and Osun. Many non-indigenes and foreigners also reside in the state and live together in harmony. Yoruba and English are the languages used for official and business transactions. The people have a rich cultural heritage that is demonstrated in all spheres of their life including the performance of FGM/C. Their culture finds expression in their arts, literature, music, and other social activities.

This study was conducted among the Yoruba TBAs residing across the three senatorial districts in Osun State, Nigeria. The Yoruba people have a rich cultural heritage that is demonstrated in all spheres of their life including the performance of FGM/C in the Osun State, Nigeria.

Research design

A qualitative research design, using the adapted REPLACE community readiness to end FGM/C interview guide [14] was used to assess the attitudes of the TBAs as community leaders towards elimination of intergenerational FGM/C to determine their level of readiness and preparedness towards achieving its elimination in Osun State, Nigeria. This was the most suitable approach because it focuses on social and cultural contexts to understand how a problem is perceived and whether taking some type of action is seen as needed by community leaders to change the problem [15, 16]. The adapted REPLACE community readiness to end FGM/C interview guide [14] was translated to the local (Yoruba) language by a bilingual expert in Yoruba and English who is also a qualitative researcher for comparison of the

language translation to avoid the loss of meaning in the translated interview guides. The same person was used for translating transcripts to English.

Sampling and recruitment of participants

Purposive sampling method was used select eight TBAs whom we were regarded as community leaders and key informants with rich information regarding intergenerational FGM/C in Osun State, Nigeria. The inclusion criteria for recruitment and participation in the study included only TBAs who were formally recognized as leaders in performing FGM/C, who had been previously involved in any of the Government's organized seminars or workshops on FGM/C elimination and or prevention intervention in Osun State, irrespective of the duration of the practice. The selection and recruitment of the participants commenced once the ethical approval from the Humanities and Social Sciences Research Ethics Committee [HSSREC/00000924/2019] and Osun state Ministry of Health [OSHREC/PRS/569T/157] was obtained. Meetings were held with the nurses in the maternity units across the three senatorial districts as gatekeepers to inform them about the purpose of the study and to seek their permission to meet the TBAs working in their respective Units. A meeting with TBAs was held where the purpose of the study, the research eligibility criteria and request for voluntary participation from those who meet the inclusion criteria were discussed. Those who showed interest were recruited and were given the information sheet and the informed consent form to complete and sign. Arrangements for the interview session were then made by allowing the potential participants to choose suitable date and time within the first 7 days after the participants have given their consent. Before the commencement of the interview, the purpose of the study, and permission to audio record the interview session were explained again to the participants in the local language (Yoruba). A total sample size of eight traditional birth attendants as community leaders and key informants were recruited in

the study. This small sample size is in line with the REPLACE community readiness tool, which suggests 6-12 key informants as sufficient to address issues in the community [14].

Pilot study

The first two TBAs out of the sample size were used for pilot study to ensure that the interview guide questions were easy and yielded the necessary data. The data generated from the pilot study were included in the main study data analysis. Contamination of data was not considered to be a concern because in qualitative studies data collection is progressive and the data from pilot studies can be used for the main study [17,18].

Data collection

Data collection and data analysis were conducted concurrently between October 2020 and March 2021. All COVID-19 precaution protocols like using the face mask, maintaining social distancing and hand sanitizing were observed during the interviews. In-depth interviews were conducted with the individual TBAs in their homes by the first author in Yoruba, the local language, using the interview guide adapted from the REPLACE Community Readiness to End FGM/C Assessment tool [14]. The interviews were audio recorded and transcribed within 24 hours of recording when information was still fresh in the researcher's memory for data analysis purposes. Each interview lasted between 45-60 minutes to complete thus allowing each TBA to adequately share their views as attitudes towards elimination of intergenerational FGM/C in Osun state. Nigeria. The transcripts were stored in word document formats on a computer with encrypted password and was backed up to an external hard drive to prevent data loss in line with the University's principles for data storage.

Data analysis

The six steps, namely: familiarization, coding, searching for themes, reviewing themes, defining, and naming themes, and writing up as described by [19] were used to analyze the transcribed data.

The first author went through the transcripts reading and re-reading them to familiarise herself and make sense of the data. Notes were taken and preliminary ideas for codes were marked. In order to start organizing the data into meaningful groups, codes were assigned to briefly describe something interesting that was said by the participants. The first author grouped the newly generated codes with same meaning together to form themes. The second author, who also doubled as the inter coder at this stage, also did the same thing. The generated themes by the first and second authors were scrutinized by both of them to ensure that they were useful and accurate representation of the data by discarding some themes and creating new ones from the transcribed data. The first author then formulated the meaning of each theme so that the data can be understood. At this stage, appropriate naming of themes was made to succinctly interpret the data collected.

Ethical Permission

All ethical principles were adhered to including, consent form for voluntary participation, ethical approval from the Humanities and Social Sciences Research Ethics Committee [HSSREC/00000924/2019] and Osun state Ministry of Health [OSHREC/PRS/569T/157] were obtained.

The names assigned to each of the participants are not their actual names as we are aware of the ethical implication. However, using pseudonyms instead of participants' actual names ensured confidentiality and anonymity.

Measure to ensure trustworthiness

Measures to ensure trustworthiness and rigor of the findings were applied through the criteria of credibility, confirmability, dependability, and transferability [20].

To ensure credibility, comprehensive field notes, noting gesture and other non-verbal ques observed during the interviews were recorded and analyzed. Audit trail of all research processes were recorded. Analysis of the data gathered reflected a neutral interpretation of the data rather than the researcher's view as recommended by [21,22]. This was achieved by conducting a member checking on data whereby the analyzed data was summarized and provided to the participants for them to validate and determine the accuracy of the data as their views. For dependability and transferability, an audit trail was kept and the comprehensive and rich details of the study setting, and the socio-demographic characteristics of the participants are presented. To further establish trust and confidence in the findings, the authors compared their individually generated themes from the raw data to ensure that they were useful and accurate representation of the data by discarding some themes and creating new ones from the transcribed data. The senior author and research supervisor reviewed the themes against raw data and helped in refining these to reflect participants' views to enhance confirmability. Common issues recur and main themes were identified to summarize all the views that have been captured in the data. One theme and three subthemes emerged that revealed the defensive attitude of the TBAs towards the elimination of intergenerational FGM/C of girls in Osun state, Nigeria.

Results

Eight (8) TBAs, comprising of three females and five males participated in the study on TBA's attitude towards the elimination of intergenerational FGM/C of girls in Osun State, Nigeria.

Table 1: Participants' Demographic Characteristics

Participant	Age	Gender	Religion	Educational level
Lukman	45	Male	Islam	Tertiary education
Abike	70	Female	Islam	Primary education
Adeyemi	53	Male	Islam	Tertiary education
Olalekan	86	Male	Islam	Primary education
Mojirola	63	Female	Christian	Secondary education
Amope	80	Female	Islam	Primary education
Fadipe	73	Male	Islam	Secondary education
Adebayo	75	Male	Islam	Primary education

These demographics reflects the TBAs in Osun State where the person who becomes a TBA is culturally determined through the lineage of the traditional circumcisers. It is a taboo for a woman who is married to perform FGM/C as TBA, but she can still retain her knives for performing FGM/C on her own children only (personal conversation with Mr Adeyemi, a male participant to the study, December 2020). This explains why most participants were male TBAs in the study as reflected in Table 1. Their ages ranged between 45 and 86 years. Seven of them practiced Islam and only one was Christian as a religion.

Defensive attitude towards elimination of intergenerational FGM/C

Analyzed data yielded "Defensive attitude towards elimination of FGM/C" as the main theme in the TBAs' attitude towards the elimination of intergenerational FGM/C of girls in Osun State, Nigeria. Three subthemes, including (i) FGM/C as a traditional heritage, (ii) Defiance against government's efforts to eliminate intergenerational FGM/C and Debunking all "western" information about dangers of FGM/C as lies emerged under the main theme.

FGM/C as traditional heritage

The FGM/C as a traditional heritage sub theme emerged from data related TBAs' defensive attitudes towards elimination of intergenerational FGM/C as traditional and cultural beliefs that the practice is the identity and the only link between the current and past among generations as described in the following extracts.

The response from the respondent below upheld the fact that FGMC is their traditional heritage that cannot be stopped in their lifetime.

I am talking about our traditional heritage here. It can't be stopped; it can't stop now. The law, civilization coupled with ignorance is stopping people, but it cannot be stopped. [Lukman, Male, Age: 45, Tertiary Education]

Another respondent also mentioned that FGM/C is a traditional heritage that was handed down by their forefathers.

I think all they are saying about stopping of our tradition is done out of civilization and hatred, hatred of us and our traditional heritage. What has been established by our forefathers cannot perish. It is just like asking us to abandon our language because of civilization. There is no where we meet that we can do without speaking our language. Girl child circumcision cannot perish. [Abike, Female, Age:70, Primary Education]

Further, data under this subtheme showed that TBAsconsiderate as their primary responsibility to prevent intergenerational practice of FGM/C from extinction as depicted in the following extract.

Girl circumcision has been in existence from time immemorial. Therefore, it can be described as our cultural identity that was transmitted from generation to generation that no one is ready to allow it to die down in his or her own time, even if your government says it is bad for us. [Fadipe, Male, Age:73, Secondary Education]

It was also reported as shown in the extract below that the TBAs have a duty to protect FGM/C being their inheritance.

But you know, it is our heritage which must be promoted. It is not that it really makes more money for us. Do you understand me? It is not what the TBAs are eating from. But it is what was handed over to us as our heritage and must not be taken away just like that! [Abike, Female, Age: 70, Primary Education].

Defiance against government's efforts toeliminate FGM/C

This subtheme emerged from data related to the defiant attitudes of the TBAs towards seminars and workshops that had been previously arranged by government and other organizations in the country with the aim of highlighting the disadvantages of intergenerational FGM/C proposals towards eliminating FGM/C as described in the following extracts.

The extract below shows that despite the attendance at the seminar organized for the TBA by the government, stopping FGMC is going to be a disadvantage to the women because their sexual urge may not be easily controlled as we are seeing around us.

I attended the workshops about this, but I was and am still convinced that the disadvantages of government's effort to stopping FGM/C will be enormous for our women. See what is happening nowadays in our communities. No one will be able to control these girls. We must fight anyone including the government to protect our girls from promiscuity. [Lukman, Male, Age: 45, Tertiary Education]

The following extract upholds the submission by the TBAs that FGM/C prevents promiscuity which explains why the tradition has been successfully continued.

No, I don't agree with the government's decision. The result will be very bad. As for me, I did it for all my children both boys and girls. None of my children are promiscuous because I circumcised them and took care of them all. I buried what was cut and properly watered the soil where I buried it so that chicken could not be able to unearth it. Otherwise, if unearthed, the girl child will be promiscuous. We are very careful about these things so that no one can come back and say FGM/C doesn't work. [Amope, Female, Age: 80, Primary Education].

A respondent bluntly declared that she will never support the government in stopping FGM/C as depicted in the following extract.

Ah! I am not in support of the government stopping the girl child circumcision. That is our campaign, and I am not hiding it. At least, it will not happen in my time. [Abike, Female, Age 70, Primary Education]

Elimination of FGM/C is described as an advantage to the girl child that no one would want to support as shown in the quote below.

Personally, I don't agree with government's reasons to stop girl child circumcision. I have said it earlier that, based on my experience and the level of my education, and coming from a family that is traditionally practicing FGM/C, I do not see it being stopped by anyone, even the government or anyone else, not now. I did it (FGM/C) for my girls and there is no problem associated with it, I don't think it should be stopped. It will be a great disadvantage if anyone could agree with government. [Lukman, Male, Age: 45, Tertiary Education].

Debunking all "Western" information about dangers of FGM/C to women as lies

This subtheme emerged from data relatedto TBAs efforts in debunking most of the information and claims about the benefits of eliminating intergenerational FGM/C as just government's efforts to brainwash people, against the practice of FGM/C as depicted in the quotes below.

The information about the girls having reproductive challenges later in life was debunked by the respondent as shown in the quote below.

The girls that I circumcised did not have any problem. Even when grown up, these girls do not develop any of the problems we were told resulted from FGM/C, which we were now told

can be associated with FGM/C. The girls are now big women and are all alive and doing well in their families. They got married to nice men and have lively children. [Amope, Female, Age 80, Primary Education]

The extract below shows that the respondent disagrees with the record of death sequel to FGM/C. He said that the girls he circumcised as infants are still alive today.

Every girl that we have circumcised is still alive even today. No child can die in the hand of circumcision surgeon and all our girls were circumcised. Anyone who say we put our girls in danger is a liar. My wife is circumcised, all my girls are circumcised and alive. They must stop spreading lies about FGM/C. [Olalekan, Male, Age 86, Primary Education]

In addition, the likelihood of lack of enjoyment during sex was further refuted but rather held that circumcised women enjoy sex more as shown in the quote below.

That debunked FGM/C leads to women not enjoying sex is one of the lies the government is peddling about girl circumcision. But they have forgotten that it's only a small part of the clitoris that is cut and not the whole clitoris. it is not true at all. [Adeyemi, Male, Age 53, Tertiary Education]

It is only a false assumption that circumcised women do not enjoy sex. When a circumcised woman has sexual intercourse with men, she will glue to him as if they tie penis and vagina together, in that way, they both enjoy each other nicely [Olalekan, Male Age: 86, Primary Education]

Furthermore, the views that FGM/C have complications was refuted as just rumors as captured by the following extracts.

There is nothing like transmission of sexual diseases by FGMC, bleeding and all so many other things that are being said about female circumcision. It is safe and cultural to do it (circumcision). [Mojirola, Female, Age 63, Secondary Education]

We were also told that female circumcision causes sexual transmitted diseases / infections which is not true at all! Honestly, I don't know what the government want to gain from spreading such lies for stopping it. Female circumcision does not have any negative side effects as it is being peddled around. [Amope, Female, Age 80, Primary Education].

Discussion

The study was conducted to assess TBAs' attitudes towards the elimination of intergenerational FGM/C of girls in Osun state, Nigeria in order to determine their level of readiness and preparedness towards achieving its elimination.

Our findings show a deep defensive attitude towards elimination of FGM/C among the TBAs as community leaders and custodians of the intergenerational FGM/C which is reflected in the participants views that FGM/C is their traditional heritage and its elimination can be equated to eliminating their cultural identity. This finding conforms with the conclusion of Dunn that the cultural identity of the Africans is being eliminated by current civilization [23] and Obi, Adesoye, Labiran that also reported in their study that, culture/tradition is the greatest human inheritance, since it contributes to the preservation of history and identity of any race, thus confirming the TBAs' beliefs about their responsibility to preserve intergenerational FGM/C as their traditional heritage despite all its disadvantages to women, their families, and communities [24].

Findings from this study also depicted a strong defiance against the government's prevention interventions as indicated in the defiance shown by those TBAs who had attended seminars

and workshops organised by the government on the elimination of intergenerational FGM/C, thus inconsistent with the previous findings by [25] who concluded that unsuccessful elimination of intergenerational FGM/C was due the government's inability to involve the major community stakeholders such as the TBAs.

Therefore, due to the TBAs' defiance against governments' efforts to eliminate FGM/C, there is a continuous non-alignment between government agencies and the views and beliefs of those responsible for promotion of historic traditions (such as FGM/C) always ending up in disagreement, if not properly handled. According to the TBAs', their voices and understanding of their attitudes towards abandoning their age long custom, and not only inviting them to attend seminars or workshops on elimination of FGM/C is important in determining the community's readiness for development and implementation of interventions for eliminating intergenerational FGM/C of girls.

Most of the information and claims about the benefits of eliminating FGM/C were debunked by the TBAs as shown from the analysis. They claimed that it is just government's efforts to brainwash people which is disputed by previous report which supports the government's assertion on the benefits of eliminating intergenerational FGM/C. This finding is also in contrast with the conclusion of [26] who reported that more patients with complications of FGM/C such as difficult delivery, perineal laceration, hemorrhage, scar/keloid etc. were seen by nurses/midwives than doctors in Yenegoa, Bayelsa, Nigeria [26].

The TBAs are of the opinion that the involvement of people who are not traditionally entitled to circumcision and are not knowledgeable enough are the ones causing the complications as stated by the government. The TBAs' debunking of all "Western" information about dangers of FGM/C to women as lies may be due to their lack of knowledge of the dangers and complications of FGM/C. Similar findings were reported by [26] who concluded limited

knowledge about complications may be reflected as negative attitudes towards prevention or elimination of intergenerational FGM/C in their lifetime.

Our findings show that FGM/C is going to continue in Osun state, Nigeria despite government’s efforts to eliminate intergenerational FGM/C. This is confirming the report by the UNFPA which estimated that because of population growth, additional 4.6 million girls are projected to be at risk of undergoing female genital mutilation by 2030 [27]. It was also reported that the outbreak of COVID-19 between 2020 and 2022 aggravated the vulnerability of girls and women who are at risk of FGM/C which indicated that 2 million cases of FGM/C could be recorded over the next decade due to COVID-19 alone [27].

Limitation

Using local language to collect data could have distorted the presentation of the questions but back and forth translation of the questions minimized the language bias.

Conclusion

The current defensive attitudes of the TBAs as community leaders are worrisome because they are an indication of the community leaders’ level of no readiness for intervention programmes for the elimination of intergenerational FGM/C in Osun state, Nigeria.We recommend that the government, policy makers and health professionals elicit and carefully consider the views of the community leaders such as TBAs who are custodians of the FGM/C before introducing measures to eliminate FGM/C in traditional communities. This may include having non-confrontational dialogue between all stakeholders for understanding of the dangers and benefits of FGM/C to the nation which can be shared from everyone for best solutions to be found.

Acknowledgement

Our sincere appreciation goes to the management of the Ministry of Health in Osun state Nigeria and University of KwaZulu-Natal for granting us the ethical approval to carry out the study. We also thank the Nurses and the Traditional Birth Attendants for creating time to grant the interviews.

Data Availability Statement: This is a qualitative study that involves 8 traditional birth attendants who agreed to participate in this study based on anonymity that all information provided will be strictly confidential. All transcripts were anonymized including the extracts that support the study's findings. Transcripts are available for research purposes only in line with the approved research protocol. This is in fulfilment of the participants confidentiality as indicated in the approved research protocol from the Humanities and Social Sciences Research Ethics Committee [HSSREC/00000924/2019] and Osun state Ministry of Health [OSHREC/PRS/569T/157] dated 20th April 2020 and 19th August 2019 respectively.

Funding: The corresponding author received student scholarship from the University of KwaZulu-Natal, South Africa through the College of Health Sciences student Scholarship.

Competing interests: The authors have no competing interests of any form as regards this study.

References

1. World Health Organization. Female Genital Mutilation. Fact sheet. 2020 Retrieved February 20, 2022, from http://www.who.int/mediacentre/factsheets/fs241/en/
2. Waigwa S, Doos L, Bradbury-Jones, Taylor J. "Effectiveness of health education as an intervention designed to prevent female genital mutilation/cutting (FGM/C): a systematic review." Reproductive health 2018; **15**(1): 62.
3. United Nations, 2015. 'The Millennium Development Goals Report 2015', pg. 38. http://www.un.org/millenniumgoals/2015_MDG_Report/pdf/MDG%202015
4. UNICEF. Female Genital Mutilation/Cutting: A Global Concern UNICEF, New York, 2016.
5. World Health Organization. Female genital mutilation: Key facts. 2018 [cited 1 May 2022]. https://www.who.int/news-room/fact-sheets/detail/female-genital-mutilation
6. UNFPA-UNICEF Joint Programme on the Elimination of Female Genital Mutilation (2019).
7. Nigeria Demographic and Health Survey (2013). National Population Commission. Federal Republic of Nigeria.
8. Berg RC, Denison E. Health Care for Women International A Tradition in Transition: Factors Perpetuating and Hindering the Continuance of Female Genital Mutilation / Cutting (FGM / C) Summarized in a Systematic Review A Tradition in Transition: Factors Perpetuating Genital. 2013;9332(October 2017).
9. Yaya, S. and B. Ghose (2018). "Female Genital Mutilation in Nigeria: A Persisting Challenge for Women's Rights." Social Sciences 7(12): 244.
10. Reisel, D. and S. M. Creighton (2015). "Long term health consequences of Female Genital Mutilation (FGM)." Maturitas 80(1): 48-51.

11. Violence against Persons (Prohibition) Act,2015, Laws of the Federation of Nigeria

12. National Population Commission of Nigeria. Nigeria Population and Housing Census 2006. Abuja, Nigeria: National Population Commission of Nigeria.

13. Omole, F.K., Lukman, Y. and Baki, A.I. (2014), "Analysis of market typology and functions in the development of Osun state, Nigeria", International Journal of Development and Sustainability, Vol. 3 No. 1, pp. 55-69

14. Barret H. Alhassan Y. The REPLACE Community Readiness to End FGM Assessment. Practical Handbook (based on REPLACE Approach www.replacefgm2.eu)2016, Coventry university, UK.

15. McCoy H.V, Malow R., Edwards R.W., Thurland A., Rosenberg R. A Strategy for Improving Community Effectiveness of HIV/AIDS Intervention Design: The Community Readiness Model in the Caribbean, Substance Use & Misuse,2007; 42:10, 1579-1592, DOI: 10.1080/10826080701212535

16. Borrayo, E. A. (2007). "Using a community readiness model to help overcome breast health disparities among US Latinas." Substance use & misuse**42**(4): 603-619.

17. Duma, S. Women's journey of recovery from sexual assault trauma: a grounded theory.2006.

18. Van Teijlingen, E R, Hundley V. "The importance of pilot studies."(2001).

19. Braun V, Clarke V. Thematic analysis. in H. Cooper, P. M. Camic, D. L. Long, A. T. Panter, D. Rindskopf, & K. J. Sher (Eds.), *APA handbook of research methods in psychology, 2012;Vol. 2. Research designs: Quantitative, qualitative, neuropsychological, and biological* (pp. 57–71). American Psychological Association. https://doi.org/10.1037/13620-004

20. Louis Cohen L, Manion L, Morrison K. Action research. Research methods in education, Routledge 2013:368-385.

21. Polit, D. and C. Beck. "Essentials of nursing research." Ethics2012; **23**(2).

22. Fawcett J, Garity J. "Chapter 1: Research and evidence-based nursing practice." Evaluating research for evidence-based nursing: (2009); 3-20.

23. Dunn F. Is It Possible to End Female Circumcision in Africa? Clin J Obstet Gynecol. 2021;1: 007-013. https://doi.org/10.29328/journal.cjog.1001002

24. Obi A.I., Adesoye O., LabiranA. Female Genital Mutilation: Attitude and Practices among Women in Okada Community, Edo State JMBR: A Peer-review Journal of Biomedical Sciences December 2015, Vol. 14 No. 2 pp 138-150

25. Andarge, MasreshaYazew. *The difficulties of ending Female Genital Mutilation (FGM): Case of Afar pastoralist communities in Ethiopia. Social Policy for Development (SPD) (2014, December 12).* Retrieved from http://hdl.handle.net/2105/17487

26. Ibrahim I.A, Oyeyemi A.S, Ekine A.A. Knowledge, Attitude and Practice of Female Genital Mutilation among Doctors and Nurses in Bayelsa state, Niger-Delta of Nigeria. International Journal of Medicine and Biomedical Research Volume 2 Issue 1 January – April 2013

27. UNFPA. 2020. 'Impact of the COVID-19 Pandemic on Family Planning and Ending Gender-Based Violence, Female Genital Mutilation and Child Marriage.' Interim Technical Note.

1. World Health Organization. Female Genital Mutilation. Fact sheet. 2020 Retrieved February 20, 2022, from http://www.who.int/mediacentre/factsheets/fs241/en/

2. Hassan H., Abd-ELhakam F., Ebrahim R., Mohammed M. Study Females' Attitude toward Female Genital Mutilation. Journal of Gynecology and Women's Health, 2022, 24(3): 1-9 DOI: 10.19080/JGWH.2022.24.556137

3. Mohammed M., Abd-ELhakam F., Hassan H., Ebrahim R. Study Females' Intention to practice Female Genital Mutilation for their Daughters at Beni-Suef. Public Health Open Access, 2022; 6(2):1-8: DOI: 10.23880/phoa-16000224

4. Ebrahim R., Abd-ELhakam F., Hassan H., Mohammed M. Relationship between Females' Knowledge and Attitude and Intention of Female Genital Mutilation. Research in Psychology and Behavioral Sciences, 2023; 11(1): 1-6. DOI: 10.12691/rpbs-11-1-1

5. Abd-ELhakam F., Ebrahim R., Mohammed M., Hassan H. Relationship between Socio Demographic Data and Female Genital Mutilation. Archives of Medical Case Reports and Case Study, 2022; 6(5); 1-7. DOI:10.31579/2692- 9392/160

6. Hassan H., Abd-ELhakam F., Ebrahim R., Mohammed M. Call for Change Enhancement Upper Egyptian Females' Knowledge Regarding Effect of Female Genital Mutilation. American Journal of Public Health Research, 2023; 11(1): 5-12. DOI: 10.12691/ajphr-11-1-2

7. Waigwa S, Doos L, Bradbury-Jones, Taylor J. "Effectiveness of health education as an intervention designed to prevent female genital mutilation/cutting (FGM/C): a systematic review." Reproductive health 2018; 15(1): 62.

8. United Nations, 2015. 'The Millennium Development Goals Report 2015', pg. 38. http://www.un.org/millenniumgoals/2015_MDG_Report/pdf/MDG%202015

9. Hassan H., Abd-ELhakam F., Zakria M., El-sayed R. Female Genital Mutilation in Northern Upper Egypt: Prospects of Reasons for Performing and Refusing. Nursing and Care Open Access Journal, 2023; 9(1):6‒10. DOI: 10.15406/ncoaj.2023.09.00251

10. Abd- Elhakam F., Ibrahim R., Mohamed M., Hassan H. Prevalence of Female Genital Mutilation at Beni-Suef Governorate, Egypt. Egyptian Journal of Health Care, (EJHC), 2023; 14 (1): 564-577. Doi: 10.21608/EJHC.2023.285610

11. Ebrahim R., Mohammed M., Hassan H., Abd-ELhakam F. Relationship between Personal Characteristics and the Person Performing Female Genital Mutilation. American Journal of Public Health Research, 2023; 11(2): 69-74. DOI: 10.12691/ajphr-11-2-5

12. Abd-ELhakam F., Ebrahim R., Mohammed M., Hassan H. Female Genital Mutilation: Females' Related Knowledge. EC Nursing Health, 2023; 5(4): 29-38.

13. Hassan H., Mohammed M., Abd-ELhakam F., Ebrahim R. Female Genital Mutilation: Study Sexual Issues. Journal of Gynecology and Women's Health, 2023, 24(4): 1-8. DOI: 10.19080/JGWH.2023.23.556145

14. UNICEF. Female Genital Mutilation/Cutting: A Global Concern UNICEF, New York, 2016.

15. Hassan H., Ebrahim R., Mohammed M., Abd-ELhakam F. Female Genital Mutilation: Impact on Knowledge, Attitude, Sexual Score Domains and Intention of Females in Northern Upper Egypt. Archives of Medical Case Reports and Case Study, 2022; 6(5): 1-8. DOI: https://doi.org/10.31579/2692-9392/161

16. Abd-ELhakam F., Ebrahim R., Mohammed M., Hassan H. Female Genital Mutilation: Relationship Association between Upper Egyptian Females' Knowledge and Attitude. International Journal of Clinical Studies & Medical Case Reports, 2023; 29(4): 1-5. DOI: 10.46998/IJCMCR.2023.29.000716

17. World Health Organization. Female genital mutilation: Key facts. 2018 [cited 1 May 2022]. https://www.who.int/news-room/fact-sheets/detail/female-genital-mutilation

18. Hassan H., Abd-ELhakam F., Ebrahim R., Mohammed M. Female Genital Mutilation: Females' Intention in Northern Upper Egypt. Journal of Obstetrics Gynecology and Reproductive Sciences, 2023; 7(1): 1-11. DOI: 10.31579/2578-8965/144

19. Saber N., Abdallah A., Hassan H. Validation of School-Based Health Education on Knowledge and Attitude of Female Students towards Female Genital Mutilation. International Egyptian Journal of Nursing Sciences and Research (IEJNSR), 2023; 4 (1): 85-102.

20. UNFPA-UNICEF Joint Programme on the Elimination of Female Genital Mutilation (2019).

21. Hassan H., Ebrahim R., Mohammed M., Abd-ELhakam F. Prevalence and Determinants of Female Genital Mutilation at Beni-Suef. Journal of Gynecology and Women's Health, 2022, 24(3): 1-10. DOI: 10.19080/JGWH.2022.23.556138

22. Abd-ELhakam F., Ebrahim R., Mohammed M., Hassan H. Relationship between Women's Intention for Female Genital Mutilation and their Exposure to Complication and Pressure. Journal of Community Medicine and Public Health Reports 2022; 3(10): 1-9. Doi.Org/10.38207/JCMPHR/2022/DEC031002139

23. Hassan H., Abd-ELhakam F., Ebrahim R., Mohammed M. Prevalence of Female Genital Mutilation in Northern Upper Egypt. American Journal of Nursing Research, 2023, 11(1): 12-20. DOI: 10.12691/ajnr-11-1-2

24. Nigeria Demographic and Health Survey (2013). National Population Commission. Federal Republic of Nigeria.

25. Berg RC, Denison E. Health Care for Women International A Tradition in Transition: Factors Perpetuating and Hindering the Continuance of Female Genital Mutilation /Cutting (FGM / C) Summarized in a Systematic Review A Tradition in Transition: Factors Perpetuating Genital. 2013; 9332(October 2017).

26. Yaya, S. and B. Ghose (2018). "Female Genital Mutilation in Nigeria: A Persisting Challenge for Women's Rights." Social Sciences 7(12): 244.

27. Reisel, D. and S. M. Creighton (2015). "Long term health consequences of Female Genital Mutilation (FGM)." Maturitas 80(1): 48-51.

28. Violence against Persons (Prohibition) Act, 2015, Laws of the Federation of Nigeria

29. National Population Commission of Nigeria. Nigeria Population and Housing Census. 2006. Abuja, Nigeria: National Population Commission of Nigeria.

30. Omole, F.K., Lukman, Y. and Baki, A.I. (2014), "Analysis of market typology and functions in the development of Osun state, Nigeria", International Journal of Development and Sustainability, Vol. 3 No. 1, pp. 55-69

31. Barret H. Alhassan Y. The REPLACE Community Readiness to End FGM Assessment. Practical Handbook (based on REPLACE Approach www.replacefgm2.eu) 2016, Coventry university, UK.

32. McCoy H.V, Malow R., Edwards R.W., Thurland A., Rosenberg R. A Strategy for Improving Community Effectiveness of HIV/AIDS Intervention Design: The Community Readiness Model in the Caribbean, Substance Use & Misuse, 2007; 42:10, 1579-1592, DOI: 10.1080/10826080701212535

33. Borrayo, E. A. (2007). "Using a community readiness model to help overcome breast health disparities among US Latinas." Substance use & misuse 42(4): 603-619.

34. Duma, S. Women's journey of recovery from sexual assault trauma: a grounded theory.2006.

35. Van Teijlingen, E R, Hundley V. "The importance of pilot studies." (2001).

36. Braun V, Clarke V. Thematic analysis. in H. Cooper, P. M. Camic, D. L. Long, A. T. Panter, D. Rindskopf, & K. J. Sher (Eds.), APA handbook of research methods in psychology, 2012;Vol. 2. Research designs: Quantitative, qualitative, neuropsychological, and biological (pp. 57–71). American Psychological Association. https://doi.org/10.1037/13620-004

37. Louis Cohen L, Manion L, Morrison K. Action research. Research methods in education, Routledge 2013: 368-385.

38. Polit, D. and C. Beck. "471 Essentials of nursing research." Ethics 2012; 23(2).

39. Fawcett J, Garity J. "Chapter 1: Research and evidence-based nursing practice." Evaluating research for evidence-based nursing: (2009); 3-20.

40. Dunn F. Is It Possible to End Female Circumcision in Africa? Clin J Obstet Gynecol. 2021; 1: 007-013. https://doi.org/10.29328/journal.cjog.1001002

41. Obi A.I., Adesoye O., Labiran A. Female Genital Mutilation: Attitude and Practices among Women in Okada Community, Edo State JMBR: A Peer-review Journal of Biomedical Sciences December 2015, Vol. 14 No. 2 pp 138-150

42. Andarge, Masresha Yazew. The difficulties of ending Female Genital Mutilation (FGM): Case of Afar pastoralist communities in Ethiopia. Social Policy for Development (SPD) (2014, December 12). Retrieved from http://hdl.handle.net/2105/17487

43. Ibrahim I.A, Oyeyemi A.S, Ekine A.A. Knowledge, Attitude and Practice of Female Genital Mutilation among Doctors and Nurses in Bayelsa state, Niger-Delta of Nigeria. International Journal of Medicine and Biomedical Research Volume 2 Issue January – April 2013

44. UNFPA. 2020. 'Impact of the COVID-19 Pandemic on Family Planning and Ending Gender-Based Violence, Female Genital Mutilation and Child Marriage.' Interim Technical Note.

Map of Osun State, Nigeria showing the various towns

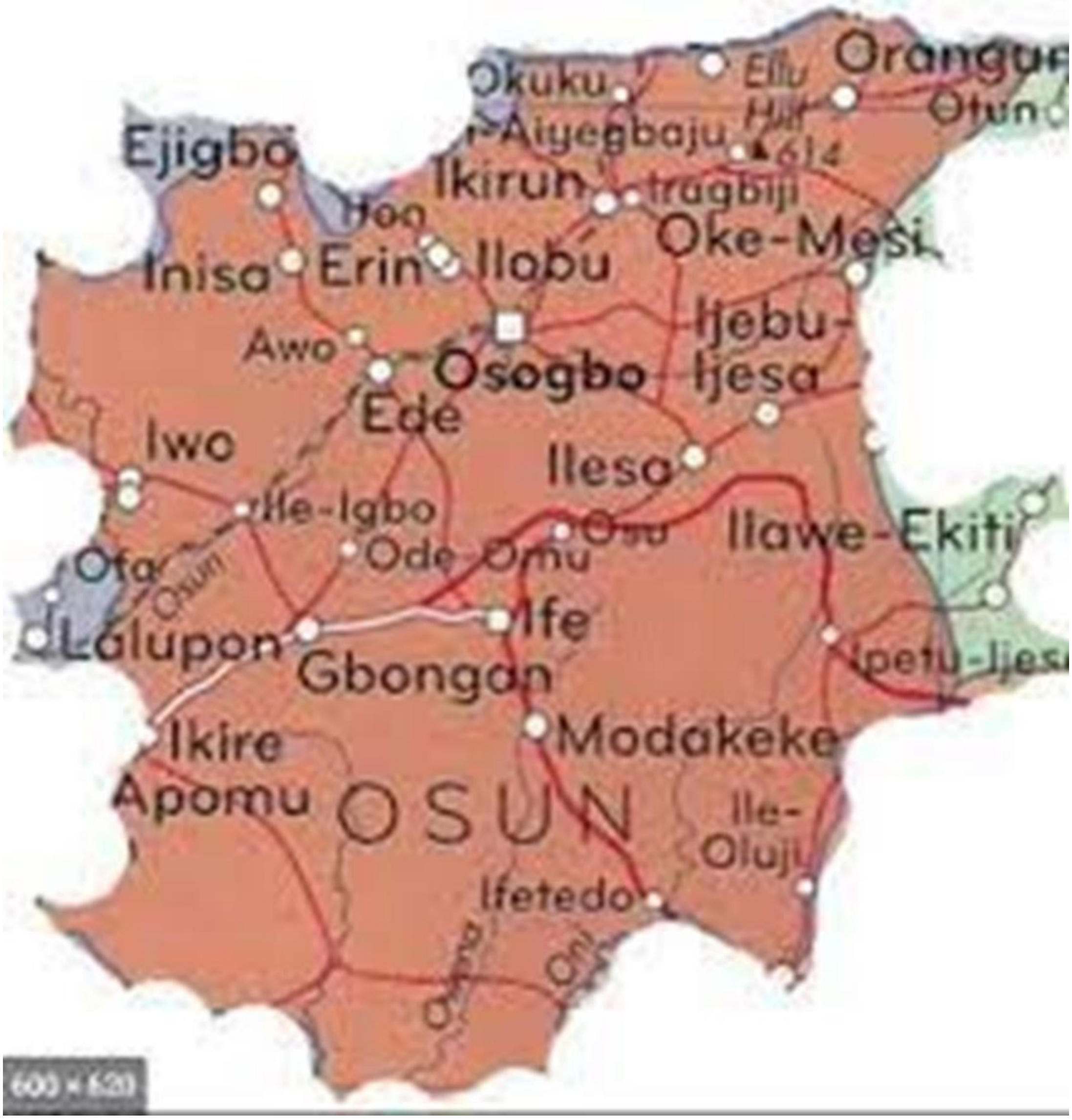

Adapted from https://dawncommission.org

Manuscript Click here to access/download;Manuscript;Clean copy of Corrected Manuscript.docx

Full Title:

'It Is What Was Handed Over to Us as Our Heritage and Must Not Be Taken Away Just Like That': Traditional Birth Attendants' Attitudes Towards the Elimination of Intergenerational Female Genital Mutilation/Cutting in Osun State, Nigeria.

Short Title:

Eliminating Intergenerational Female Genital Mutilation/Cutting in Osun State, Nigeria.

Authors:

ROSEMARY OMOLARA FAFOWORA[1¶*ID] orcid.org/0000-0003-2968-3784

SINEGUGU EVIDENCE DUMA[2¶ID] orcid.org/0000-0002-2489-8770

[1]Department of Nursing, School of Nursing and Public Health, College of Health Sciences, Howard Campus, University of KwaZulu-Natal, Durban, South Africa.

[2]Department of Nursing, School of Nursing and Public Health, College of Health Sciences, Howard Campus, University of KwaZulu-Natal, Durban, South Africa.

*Corresponding author

E-mail:rosemary.olagunju@gmail.com (RO)

Abstract

Background

Female genital mutilation/cutting is a harmful practice that violates the sexual and reproductive health rights of women and girls. The practice is often perpetrated directly or indirectly from one generation to another as a way of preserving culture, thus making it difficult to tackle using ordinary prevention interventions. The purpose of the study was to assess the attitude of the traditional birth attendants as community leaders towards the elimination of intergenerational female genital mutilation/cutting (FGM/C) of girls and to determine their level of readiness and preparedness towards achieving it in Osun State, Nigeria.

Methodology/Results

A qualitative research design, using the adapted REPLACE community readiness tool to end female genital mutilation/cutting interview guide, was used to individually interview eight traditional birth attendants who were identified through purposive sampling method as community leaders and key informants. Thematic Analysis was used to analyze the data which yielded female genital mutilation/cutting as traditional heritage, defiance against government efforts and debunking all “western” information about dangers of female genital mutilation to women as lies as findings. The current defensive attitudes of the TBAs as community leaders and custodians of the FGM/C tradition are an indication of low level of no readiness for any intervention to prevent or eliminate FGM/C in Osun state, Nigeria.

Conclusion

Serious engagement and dialogue between policy makers and health professionals on FGM/C and its effects on women is highly recommended for effective FGM/C elimination strategies

to be co-developed with community leaders. Such engagements should adopt a non-confrontational, respectful, and honest approach so as to not alienate the TBAs.

Key Words: Replace Community Readiness Tool, Intergenerational Female Genital Mutilation/Cutting, Traditional Birth Attendant

Introduction

Female genital mutilation/cutting (FGM/C) is a public health problem globally. It comprises all procedures that involve partial or total removal of the external female genitalia, or other injury to the female genital organs for non-medical reasons. It is also referred to as multiple procedures on the female genital organs for non-medical reasons [1]. FGM/C has been described as a harmful practice that violates the sexual and reproductive health rights of women and girls [2,3]. It is estimated that more than 200 million girls and women alive today have undergone FGM/C across 30 countries including the Middle East, Asia, and Africa [4]. Of this, about 44 million are girls under the age of 15 years [5]. It is further projected that more than 3 million girls and women are currently at risk of FGM/C worldwide [4]. The current estimates of 3.9 million girls mutilated annually will also rise to 4.6 million by 2030 because there is an increase in the population growth in the communities that still practice FGM/C [6]. It is reported that 20 million girls and women are estimated to have undergone FGM/C in Nigeria, with Osun state, where the study was conducted, having reached the highest prevalence of 76.6% of FGM/C [7].

In Nigeria, FGM/C is a deeply entrenched custom that is commonly performed by the traditional birth attendants (TBAs) who claim it as a heritage which confers honor on a woman and her family, a rite of passage to womanhood, preservation of chastity,

enhancement of fertility, and prevention of death of the child during delivery among its benefits [8].

Despite its cultural importance, FGM/C has received considerable criticism worldwide because of its potential risk for various forms of health complications [9]. The short term and physical health complication of FGM/C includes but are not limited to pain, risk of hemorrhage, shock, tetanus/sepsis, inability to urinate, damage to adjacent organs such as the rectum and urinary tract, dislocation and fracture of the pelvic bones and the head of the femur due to struggle while being restrained, open sore in the genital region, risk of HIV/AIDS or Hepatitis B. While the long term and reproductive health consequences of FGM/C include difficulty in passing menstrual blood, vaginal fistula, painful sexual intercourse, and difficulty in passing urine which can lead to recurrent urinary tract infection, clitoral cyst, infertility, an increased risk of childbirth complication and newborn death, need for future surgeries to allow for sexual intercourse and childbirth, psychological trauma, and risk of death from hemorrhage or infection [10].

Furthermore, sexuality is an equal right that both males and females must benefit from. While men are being circumcised for health and religious reasons, women and girls are cut deliberately to remove their sexual feelings and subjugate them to the dictates of their men counterpart, and it is done without any consent from them.

In 2005, the Violence Against Persons (Prohibition) Act 2015 was passed to eliminate the FGM/C practice in Nigeria. Since then, several legal, community based, and health-based strategies had been instituted to eliminate the FGM/C practice [11]. These strategies were met with strong opposition from the TBAs, who remain the self-proclaimed custodians of the traditional responsibility of FGM/C from time immemorial, especially in Osun State, Nigeria. The TBAs believe in the supremacy of FGM/C as the traditional heritage, which is concerned

about the wellbeing of the people, that it predates modern medicine and therefore should not be stopped. This cultural belief has facilitated the intergenerational preservation of FGM/C and explains their attitude towards the elimination of its practice. The intergenerational preservation of FGM/C is reflected on most women's belief that the elimination of FGM/C is tantamount to defaulting from their religion or traditions which could breed calamity for them and their offspring. These beliefs have been passed down from one generation to the other and contribute to the high prevalence of FGM/C in Osun State, Nigeria, where it has become major public health issue that requires public health approach-based prevention interventions. Because of their influence on the intergenerational preservation of FGM/C, we identified the TBAs as community leaders who are key towards the development and implementation of public health prevention intervention for FGM/C in Osun State, Nigeria. The purpose of the study was to assess the attitude of the TBAs as community leaders towards the elimination of intergenerational FGM/C of girls in Osun State, Nigeria, in order to determine their level of readiness and preparedness towards achieving its elimination.

Materials and Methods

Study setting

Osun State is an inland state in South-West Nigeria with a population of 3,423,535 [12]. The present Osun State was created in 1991 from the old Oyo State. The state shares boundary with Kwara State in the North, Oyo in the West, Ogun and Ondo in the South and Ekiti State on East. The state capital is Osogbo, which is a local government area on its own. In all, a total of thirty local government council areas and Ife east area council constitute the state. Among the major towns in the state are Ilesha, Ife, Ede, Ikirun, Ikire, Ejigbo and Osogbo [13]. Before this time, the state was part of the old Oyo State. Osun State experiences a tropical climate while the local vegetation is the lowland rainforest type.

Indigenes of Osun State belong to the Yoruba race and comprise the Oyo, Ife, Ijesa, Igbomina and Osun. Many non-indigenes and foreigners also reside in the state and live together in harmony. Yoruba and English are the languages used for official and business transactions. The people have a rich cultural heritage that is demonstrated in all spheres of their life including the performance of FGM/C. Their culture finds expression in their arts, literature, music, and other social activities.

This study was conducted among the Yoruba TBAs residing across the three senatorial districts in Osun State, Nigeria. The Yoruba people have a rich cultural heritage that is demonstrated in all spheres of their life including the performance of FGM/C in the Osun State, Nigeria.

Research design

A qualitative research design, using the adapted REPLACE community readiness to end FGM/C interview guide [14] was used to assess the attitudes of the TBAs as community leaders towards elimination of intergenerational FGM/C to determine their level of readiness and preparedness towards achieving its elimination in Osun State, Nigeria. This was the most suitable approach because it focuses on social and cultural contexts to understand how a problem is perceived and whether taking some type of action is seen as needed by community leaders to change the problem [15, 16]. The adapted REPLACE community readiness to end FGM/C interview guide [14] was translated to the local (Yoruba) language by a bilingual expert in Yoruba and English who is also a qualitative researcher for comparison of the language translation to avoid the loss of meaning in the translated interview guides. The same person was used for translating transcripts to English.

Sampling and recruitment of participants

Purposive sampling method was used select eight TBAs whom we were regarded as community leaders and key informants with rich information regarding intergenerational FGM/C in Osun State, Nigeria. The inclusion criteria for recruitment and participation in the study included only TBAs who were formally recognized as leaders in performing FGM/C, who had been previously involved in any of the Government's organized seminars or workshops on FGM/C elimination and or prevention intervention in Osun State, irrespective of the duration of the practice. The selection and recruitment of the participants commenced once the ethical approval from the Humanities and Social Sciences Research Ethics Committee [HSSREC/00000924/2019] and Osun state Ministry of Health [OSHREC/PRS/569T/157] was obtained. Meetings were held with the nurses in the maternity units across the three senatorial districts as gatekeepers to inform them about the purpose of the study and to seek their permission to meet the TBAs working in their respective Units. A meeting with TBAs was held where the purpose of the study, the research eligibility criteria and request for voluntary participation from those who meet the inclusion criteria were discussed. Those who showed interest were recruited and were given the information sheet and the informed consent form to complete and sign. Arrangements for the interview session were then made by allowing the potential participants to choose suitable date and time within the first 7 days after the participants have given their consent. Before the commencement of the interview, the purpose of the study, and permission to audio record the interview sessions were explained again to the participants in the local language (Yoruba). A total sample size of eight traditional birth attendants as community leaders and key informants were recruited in the study. This small sample size is in line with the REPLACE community readiness tool, which suggests 6-12 key informants as sufficient to address issues in the community [14].

Pilot study

The first two TBAs out of the sample size were used for pilot study to ensure that the interview guide questions were easy and yielded the necessary data. The data generated from the pilot study were included in the main study data analysis. Contamination of data was not considered to be a concern because in qualitative studies data collection is progressive and the data from pilot studies can be used for the main study [17,18].

Data collection

Data collection and data analysis were conducted concurrently between October 2020 and March 2021. All COVID-19 precaution protocols like using the face mask, maintaining social distancing and hand sanitizing were observed during the interviews. In-depth interviews were conducted with the individual TBAs in their homes by the first author in Yoruba, the local language, using the interview guide adapted from the REPLACE Community Readiness to End FGM/C Assessment tool [14]. The interviews were audio recorded and transcribed within 24 hours of recording when information was still fresh in the researcher’s memory for data analysis purposes. Each interview lasted between 45-60 minutes to complete thus allowing each TBA to adequately share their views as attitudes towards elimination of intergenerational FGM/C in Osun state. Nigeria. The transcripts were stored in word document formats on a computer with encrypted password and was backed up to an external hard drive to prevent data loss in line with the University’s principles for data storage.

Data analysis

The six steps, namely: familiarization, coding, searching for themes, reviewing themes, defining, and naming themes, and writing up as described by [19] were used to analyze the transcribed data.

The first author went through the transcripts reading and re-reading them to familiarise herself and make sense of the data. Notes were taken and preliminary ideas for codes were marked. In order to start organizing the data into meaningful groups, codes were assigned to briefly describe something interesting that was said by the participants. The first author grouped the newly generated codes with same meaning together to form themes. The second author, who also doubled as the inter coder at this stage, also did the same thing. The generated themes by the first and second authors were scrutinized by both of them to ensure that they were useful and accurate representation of the data by discarding some themes and creating new ones from the transcribed data. The first author then formulated the meaning of each theme so that the data can be understood. At this stage, appropriate naming of themes was made to succinctly interpret the data collected.

Ethical Permission

All ethical principles were adhered to including, consent form for voluntary participation, ethical approval from the Humanities and Social Sciences Research Ethics Committee [HSSREC/00000924/2019] and Osun state Ministry of Health [OSHREC/PRS/569T/157] were obtained.

The names assigned to each of the participants are not their actual names as we are aware of the ethical implication. However, using pseudonyms instead of participants' actual names ensured confidentiality and anonymity.

Measure to ensure trustworthiness

Measures to ensure trustworthiness and rigor of the findings were applied through the criteria of credibility, confirmability, dependability, and transferability [20].

To ensure credibility, comprehensive field notes, noting gesture and other non-verbal ques observed during the interviews were recorded and analyzed. Audit trail of all research processes were recorded. Analysis of the data gathered reflected a neutral interpretation of the data rather than the researcher's view as recommended by [21,22]. This was achieved by conducting a member checking on data whereby the analyzed data was summarized and provided to the participants for them to validate and determine the accuracy of the data as their views. For dependability and transferability, an audit trail was kept and the comprehensive and rich details of the study setting, and the socio-demographic characteristics of the participants are presented. To further establish trust and confidence in the findings, the authors compared their individually generated themes from the raw data to ensure that they were useful and accurate representation of the data by discarding some themes and creating new ones from the transcribed data. The senior author and research supervisor reviewed the themes against raw data and helped in refining these to reflect participants' views to enhance confirmability. Common issues recur and main themes were identified to summarize all the views that have been captured in the data. One theme and three subthemes emerged that revealed the defensive attitude of the TBAs towards the elimination of intergenerational FGM/C of girls in Osun state, Nigeria.

Results

Eight (8) TBAs, comprising of three females and five males participated in the study on TBA's attitude towards the elimination of intergenerational FGM/C of girls in Osun State, Nigeria.

Table 1: Participants' Demographic Characteristics

Participant	Age	Gender	Religion	Educational level
Lukman	45	Male	Islam	Tertiary education
Abike	70	Female	Islam	Primary education
Adeyemi	53	Male	Islam	Tertiary education
Olalekan	86	Male	Islam	Primary education
Mojirola	63	Female	Christian	Secondary education
Amope	80	Female	Islam	Primary education
Fadipe	73	Male	Islam	Secondary education
Adebayo	75	Male	Islam	Primary education

These demographics reflects the TBAs in Osun State where the person who becomes a TBA is culturally determined through the lineage of the traditional circumcisers. It is a taboo for a woman who is married to perform FGM/C as TBA, but she can still retain her knives for performing FGM/C on her own children only (personal conversation with Mr Adeyemi, a male participant to the study, December 2020). This explains why most participants were male TBAs in the study as reflected in Table 1. Their ages ranged between 45 and 86 years. Seven of them practiced Islam and only one was Christian as a religion.

Defensive attitude towards elimination of intergenerational FGM/C

Analyzed data yielded "Defensive attitude towards elimination of FGM/C" as the main theme in the TBAs' attitude towards the elimination of intergenerational FGM/C of girls in Osun State, Nigeria. Three subthemes, including (i) FGM/C as a traditional heritage, (ii) Defiance against government's efforts to eliminate intergenerational FGM/C and Debunking all "western" information about dangers of FGM/C as lies emerged under the main theme.

FGM/C as traditional heritage

The FGM/C as a traditional heritage sub theme emerged from data related TBAs' defensive attitudes towards elimination of intergenerational FGM/C as traditional and cultural beliefs that the practice is the identity and the only link between the current and past among generations as described in the following extracts.

The response from the respondent below upheld the fact that FGMC is their traditional heritage that cannot be stopped in their lifetime.

I am talking about our traditional heritage here. It can't be stopped; it can't stop now. The law, civilization coupled with ignorance is stopping people, but it cannot be stopped. [Lukman, Male, Age: 45, Tertiary Education]

Another respondent also mentioned that FGM/C is a traditional heritage that was handed down by their forefathers.

I think all they are saying about stopping of our tradition is done out of civilization and hatred, hatred of us and our traditional heritage. What has been established by our forefathers cannot perish. It is just like asking us to abandon our language because of civilization. There is no where we meet that we can do without speaking our language. Girl child circumcision cannot perish. [Abike, Female, Age:70, Primary Education]

Further, data under this subtheme showed that TBAsconsiderate as their primary responsibility to prevent intergenerational practice of FGM/C from extinction as depicted in the following extract.

Girl circumcision has been in existence from time immemorial. Therefore, it can be described as our cultural identity that was transmitted from generation to generation that no one is ready to allow it to die down in his or her own time, even if your government says it is bad for us. [Fadipe, Male, Age:73, Secondary Education]

It was also reported as shown in the extract below that the TBAs have a duty to protect FGM/C being their inheritance.

But you know, it is our heritage which must be promoted. It is not that it really makes more money for us. Do you understand me? It is not what the TBAs are eating from. But it is what was handed over to us as our heritage and must not be taken away just like that! [Abike, Female, Age: 70, Primary Education].

Defiance against government's efforts to eliminate FGM/C

This subtheme emerged from data related to the defiant attitudes of the TBAs towards seminars and workshops that had been previously arranged by government and other organizations in the country with the aim of highlighting the disadvantages of intergenerational FGM/C proposals towards eliminating FGM/C as described in the following extracts.

The extract below shows that despite the attendance at the seminar organized for the TBA by the government, stopping FGMC is going to be a disadvantage to the women because their sexual urge may not be easily controlled as we are seeing around us.

I attended the workshops about this, but I was and am still convinced that the disadvantages of government's effort to stopping FGM/C will be enormous for our women. See what is happening nowadays in our communities. No one will be able to control these girls. We must fight anyone including the government to protect our girls from promiscuity. [Lukman, Male, Age: 45, Tertiary Education]

The following extract upholds the submission by the TBAs that FGM/C prevents promiscuity which explains why the tradition has been successfully continued.

No, I don't agree with the government's decision. The result will be very bad. As for me, I did it for all my children both boys and girls. None of my children are promiscuous because I circumcised them and took care of them all. I buried what was cut and properly watered the soil where I buried it so that chicken could not be able to unearth it. Otherwise, if unearthed, the girl child will be promiscuous. We are very careful about these things so that no one can come back and say FGM/C doesn't work. [Amope, Female, Age: 80, Primary Education].

A respondent bluntly declared that she will never support the government in stopping FGM/C as depicted in the following extract.

Ah! I am not in support of the government stopping the girl child circumcision. That is our campaign, and I am not hiding it. At least, it will not happen in my time. [Abike, Female, Age 70, Primary Education]

Elimination of FGM/C is described as an advantage to the girl child that no one would want to support as shown in the quote below.

Personally, I don't agree with government's reasons to stop girl child circumcision. I have said it earlier that, based on my experience and the level of my education, and coming from a family that is traditionally practicing FGM/C, I do not see it being stopped by anyone, even the government or anyone else, not now. I did it (FGM/C) for my girls and there is no problem associated with it, I don't think it should be stopped. It will be a great disadvantage if anyone could agree with government. [Lukman, Male, Age: 45, Tertiary Education].

Debunking all "Western" information about dangers of FGM/C to women as lies

This subtheme emerged from data related to TBAs efforts in debunking most of the information and claims about the benefits of eliminating intergenerational FGM/C as just government's efforts to brainwash people, against the practice of FGM/C as depicted in the quotes below.

The information about the girls having reproductive challenges later in life was debunked by the respondent as shown in the quote below.

The girls that I circumcised did not have any problem. Even when grown up, these girls do not develop any of the problems we were told resulted from FGM/C, which we were now told

can be associated with FGM/C. The girls are now big women and are all alive and doing well in their families. They got married to nice men and have lively children. [Amope, Female, Age 80, Primary Education]

The extract below shows that the respondent disagrees with the record of death sequel to FGM/C. He said that the girls he circumcised as infants are still alive today.

Every girl that we have circumcised is still alive even today. No child can die in the hand of circumcision surgeon and all our girls were circumcised. Anyone who say we put our girls in danger is a liar. My wife is circumcised, all my girls are circumcised and alive. They must stop spreading lies about FGM/C. [Olalekan, Male, Age 86, Primary Education]

In addition, the likelihood of lack of enjoyment during sex was further refuted but rather held that circumcised women enjoy sex more as shown in the quote below.

That debunked FGM/C leads to women not enjoying sex is one of the lies the government is peddling about girl circumcision. But they have forgotten that it's only a small part of the clitoris that is cut and not the whole clitoris. it is not true at all. [Adeyemi, Male, Age 53, Tertiary Education]

It is only a false assumption that circumcised women do not enjoy sex. When a circumcised woman has sexual intercourse with men, she will glue to him as if they tie penis and vagina together, in that way, they both enjoy each other nicely [Olalekan, Male Age: 86, Primary Education]

Furthermore, the views that FGM/C have complications was refuted as just rumors as captured by the following extracts.

There is nothing like transmission of sexual diseases by FGMC, bleeding and all so many other things that are being said about female circumcision. It is safe and cultural to do it (circumcision). [Mojirola, Female, Age 63, Secondary Education]

We were also told that female circumcision causes sexual transmitted diseases / infections which is not true at all! Honestly, I don't know what the government want to gain from spreading such lies for stopping it. Female circumcision does not have any negative side effects as it is being peddled around. [Amope, Female, Age 80, Primary Education].

Discussion

The study was conducted to assess TBAs' attitudes towards the elimination of intergenerational FGM/C of girls in Osun state, Nigeria in order to determine their level of readiness and preparedness towards achieving its elimination. It is important to note that TBAs attitudes reflect a broader societal attitudes and cultural norms that is prevalent in the region.

In recent years, there have been significant efforts to promote the elimination of FGM/C in Nigeria. These efforts include education and awareness campaigns, legal measures, and community engagement programs. However, despite these efforts, our data reveals that the TBAs are still holding on to the traditional beliefs which support the practice.

Our findings show a deep defensive attitude towards elimination of FGM/C among the TBAs as community leaders and custodians of the intergenerational FGM/C which is reflected in the participants views that FGM/C is their traditional heritage and its elimination can be equated to erasing their cultural identity. Similar conclusion was made by Dunn (2018), whose research on how possible it is to end female circumcision in Africa reported that the cultural identity of the Africans was eliminated by current civilization [23]. Sakeah et al

(2019) further identified tradition/culture as one of the contributory factors to the persistent practice of FGM/C in Ghana despite it being considered illegal in that country [24]. Our findings revealed the TBAs belief that FGM/C is a cultural tradition that must not be taken away in a similar way that other traditions were eroded by the colonial masters who gradually stripped their subjects off their cultural heritage.

The findings on TBA's strong defiance against the government's prevention interventions such as seminars and workshops organised by the government on the elimination of intergenerational FGM/C is contrary to the findings by Andarge M. Y. (2014) who reported that unsuccessful elimination of intergenerational FGM/C in Ethiopia was due to the government's inability to involve the major community stakeholders such as the TBAs [25].

If not properly handled, the TBAs' defiance against governments' efforts to eliminate FGM/C, can result in a continuous non-alignment and disagreements between government agencies and those responsible for promotion of historic traditions (such as FGM/C) as was reported by Dunn (2018) [23].

According to the TBAs all the government's efforts to eliminate FGMC was perceived as the government's efforts to brainwash people, thus debunking all information and claims about the benefits of eliminating FGM/C as reported previously by Ibrahim I.A et al. 2013 where it was reported that more patients with complications of FGM/C such as difficult delivery, perineal laceration, hemorrhage, scar/keloid etc. were seen by nurses/midwives than doctors, thus indicating that eliminating FGMC will be beneficial in preventing these complications [26]. The TBA's lack of knowledge about complications of FMG/C may be reflected as negative attitudes towards elimination of intergenerational FGM/C. On the other hand, holding the viewpoint that the complications from FGM/C are due to the participation of

informal “fake” TBAs is a dangerous viewpoint that needs to be corrected if FGM/C is to be eliminated. individuals who are not typically burdened with such responsibilities.

The social isolation of women and young girls within their families during Covid-19 pandemic also put more girls at risk for FGM/C and protecting girls and women at risk. Both formal and informal TBAs could continue with the practice without anyone stopping them. This therefore confirmed the report by the UNFPA (2020) which estimated that additional 4.6 million girls could be at risk of undergoing female genital mutilation by 2030 [27].

Strength and Limitation

This study used a qualitative approach thus encouraging the TBAs as custodians of FGM/C traditions to voice their views on FGM/C freely with the researchers. Such openness helped in identifying deeply held beliefs that need to be addressed for effective elimination of FGM/C at community level. However, the fact that the gender and profession of the researcher (a woman and a nurse) could be perceived as a limitation because most TBAs were men. Some of their responses could be seen as men exerting their dominance on women.

Conclusion

The current defensive attitudes of the TBAs as community leaders and custodians of the FGMC tradition are an indication of low level of no readiness for any intervention to prevent or eliminate FGMC in Osun sate, Nigeria. Serious engagement and dialogue between policy makers and health professionals on FGMC and its effects on women is highly recommended for effective FGMC elimination strategies to be co-developed with community leaders. Such engagements should adopt a non-confrontational, respectful, and honest approach so as to not alienate the TBAs.

Acknowledgement

Our sincere appreciation goes to the management of the Ministry of Health in Osun state Nigeria and University of KwaZulu-Natal for granting us the ethical approval to carry out the study. We also thank the Nurses and the Traditional Birth Attendants for creating time to grant the interviews.

Data Availability Statement: All relevant data are within the paper and its supporting information file.

Funding: The author(s) received no specific funding for this work.

Competing interests: The authors have no competing interests of any form as regards this study.

Author Contributions

Conceptualization: Rosemary Omolara Fafowora, Sinegugu Evidence Duma.

Formal analysis: Rosemary Omolara Fafowora, Sinegugu Evidence Duma

Investigation: Rosemary Omolara Fafowora.

Methodology: Rosemary Omolara Fafowora.

Supervision: Sinegugu Evidence Duma.

Validation: Sinegugu Evidence Duma.

Writing - original draft: Rosemary Omolara Fafowora

Writing -reviewing & editing: Sinegugu Evidence Duma

References

1. World Health Organization. Female Genital Mutilation. Fact sheet. 2020 Retrieved February 20, 2022, from http://www.who.int/mediacentre/factsheets/fs241/en/
2. Waigwa S, Doos L, Bradbury-Jones, Taylor J. "Effectiveness of health education as an intervention designed to prevent female genital mutilation/cutting (FGM/C): a systematic review." Reproductive health 2018; **15**(1): 62.
3. United Nations, 2015. 'The Millennium Development Goals Report 2015', pg. 38. http://www.un.org/millenniumgoals/2015_MDG_Report/pdf/MDG%202015
4. UNICEF. Female Genital Mutilation/Cutting: A Global Concern UNICEF, New York, 2016.
5. World Health Organization. Female genital mutilation: Key facts. 2018 [cited 1 May 2022]. https://www.who.int/news-room/fact-sheets/detail/female-genital-mutilation
6. UNFPA-UNICEF Joint Programme on the Elimination of Female Genital Mutilation (2019).
7. Nigeria Demographic and Health Survey (2013). National Population Commission. Federal Republic of Nigeria.
8. Berg RC, Denison E. Health Care for Women International A Tradition in Transition: Factors Perpetuating and Hindering the Continuance of Female Genital Mutilation / Cutting (FGM / C) Summarized in a Systematic Review A Tradition in Transition: Factors Perpetuating Genital. 2013;9332(October 2017).

9. Yaya, S. and B. Ghose (2018). "Female Genital Mutilation in Nigeria: A Persisting Challenge for Women's Rights." Social Sciences 7(12): 244.
10. Reisel, D. and S. M. Creighton (2015). "Long term health consequences of Female Genital Mutilation (FGM)." Maturitas 80(1): 48-51.
11. Violence against Persons (Prohibition) Act,2015, Laws of the Federation of Nigeria
12. National Population Commission of Nigeria. Nigeria Population and Housing Census 2006. Abuja, Nigeria: National Population Commission of Nigeria.
13. Omole, F.K., Lukman, Y. and Baki, A.I. (2014), "Analysis of market typology and functions in the development of Osun state, Nigeria", International Journal of Development and Sustainability, Vol. 3 No. 1, pp. 55-69
14. Barret H. Alhassan Y. The REPLACE Community Readiness to End FGM Assessment. Practical Handbook (based on REPLACE Approach www.replacefgm2.eu)2016, Coventry university, UK.
15. McCoy H.V, Malow R., Edwards R.W., Thurland A., Rosenberg R. A Strategy for Improving Community Effectiveness of HIV/AIDS Intervention Design: The Community Readiness Model in the Caribbean, Substance Use & Misuse,2007; 42:10, 1579-1592, DOI: 10.1080/10826080701212535
16. Borrayo, E. A. (2007). "Using a community readiness model to help overcome breast health disparities among US Latinas." Substance use & misuse **42**(4): 603-619.

17. Duma, S. Women's journey of recovery from sexual assault trauma: a grounded theory.2006.
18. Van Teijlingen, E R, Hundley V. "The importance of pilot studies."(2001).
19. Braun V, Clarke V. Thematic analysis. in H. Cooper, P. M. Camic, D. L. Long, A. T. Panter, D. Rindskopf, & K. J. Sher (Eds.), *APA handbook of research methods in psychology, 2012;Vol. 2. Research designs: Quantitative, qualitative, neuropsychological, and biological* (pp. 57–71). American Psychological Association. https://doi.org/10.1037/13620-004
20. Louis Cohen L, Manion L, Morrison K. Action research. Research methods in education, Routledge 2013:368-385.
21. Polit, D. and C. Beck. "Essentials of nursing research." Ethics2012; **23**(2).
22. Fawcett J, Garity J. "Chapter 1: Research and evidence-based nursing practice." Evaluating research for evidence-based nursing: (2009); 3-20.
23. Dunn F. Is It Possible to End Female Circumcision in Africa? Clin J Obstet Gynecol. 2021;1: 007-013. https://doi.org/10.29328/journal.cjog.1001002
24. Sakeah E, Debpuur C, Aborigo RA, Oduro AR, Sakeah JK, Moyer CA (2019) Persistent female genital mutilation despite its illegality: Narratives from women and women in northern Ghana. PLoS ONE 14(14):e0214923. https://doi.org/10.1371 journal pone 0214923.
25. Andarge, MasreshaYazew. *The difficulties of ending Female Genital Mutilation (FGM): Case of Afar pastoralist communities in Ethiopia*. *Social Policy for Development (SPD) (2014, December 12).* Retrieved from http://hdl.handle.net/2105/17487
26. Ibrahim I.A, Oyeyemi A.S, Ekine A.A. Knowledge, Attitude and Practice of Female Genital Mutilation among Doctors and Nurses in Bayelsa state,

Niger-Delta of Nigeria. International Journal of Medicine and Biomedical Research Volume 2 Issue 1 January – April 2013

27. UNFPA. 2020. ‘Impact of the COVID-19 Pandemic on Family Planning and Ending Gender-Based Violence, Female Genital Mutilation and Child Marriage.’ Interim Technical Note.

The political Map of Osun State, Nigeria

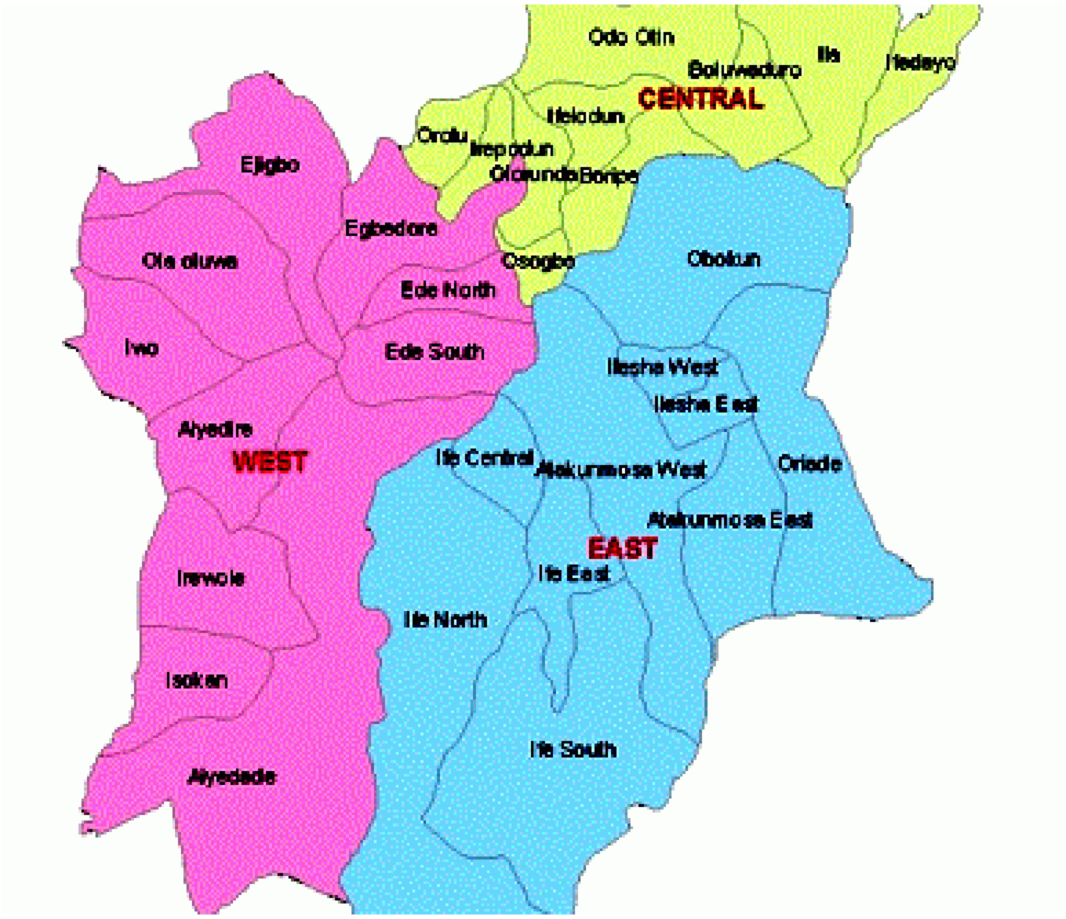

Adapted from https://dawncommission.org

Click here to access/download
Supporting Information
TRANSCRIPTS OF THE INTERVIEWS OF THE PARTICIPANTS.docx

Formatted: Numbering: Continuous

Full Title:

‘It Is What Was Handed Over to Us as Our Heritage and Must Not Be Taken Away Just Like That’: Traditional Birth Attendants’ Attitudes Towards the Elimination of Intergenerational Female Genital Mutilation/Cutting in Osun State, Nigeria.

Short Title:

Eliminating Intergenerational Female Genital Mutilation/Cutting in Osun State, Nigeria.

Authors:

ROSEMARY OMOLARA FAFOWORA[1]¶*[ID] orcid.org/0000-0003-2968-3784

SINEGUGU EVIDENCE DUMA[2]¶[ID] orcid.org/0000-0002-2489-8770

[1]Department of Nursing, School of Nursing and Public Health, College of Health Sciences, Howard Campus, University of KwaZulu-Natal, Durban, South Africa.

[2]Department of Nursing, School of Nursing and Public Health, College of Health Sciences, Howard Campus, University of KwaZulu-Natal, Durban, South Africa.

*Corresponding author

E-mail: rosemary.olagunju@gmail.com (RO)

¶These authors equally contributed to this work.

Abstract

Background

Formatted: Font: 12 pt

Female ~~G~~genital ~~M~~mutilation/~~C~~cutting ~~(FGM/C)~~ is a harmful practice that violates the sexual and reproductive health rights of women and girls. ~~It entails all procedures that involve partial or total removal of the external female genitalia or injury to the female reproductive organs, whether for cultural or any other non-therapeutic reasons.~~ Thee practice ~~of FGM/C~~ is often perpetrated directly or indirectly from one generation to ~~the~~ another as a way of preserving culture, thus making it difficult to tackle using ordinary prevention interventions. The purpose of the study was to assess ~~determine and describe t~~the attitudes of the ~~T~~traditional ~~B~~birth aAttendants ~~(TBAs)~~ as community leaders towards the elimination of intergenerational ~~FGM/C~~female genital mutilation/cutting and to determine their level of readiness and preparedness towards achieving it~~s elimination~~ in Osun State, Nigeria.

Methodology/Results

A qualitative research design, using the adapted REPLACE community readiness tool to end ~~FGM/C~~female genital mutilation/cutting interview guide, was used to individually interview eight ~~TBA~~traditional birth attendants~~s~~ who were identified through purposive sampling method as community leaders and key informants. Thematic Analysis was used to analyze the data which yielded ~~FGM/C~~female genital mutilation/cutting as traditional heritage, defiance against government efforts and debunking all “western” information about dangers of ~~FGM/C~~ female genital mutilation to women as lies as findings. These findings are worrisome because they are an indication of the community leaders’ level of no readiness for interventions for elimination of intergenerational ~~FGM/C~~ female genital mutilation/cutting in Osun state, Nigeria.

Conclusion

We recommend that the government, policy makers and health professionals elicit and carefully consider the views of the community leaders such as ~~TBAs~~ traditional birth attendants who are custodians of the ~~FGM/C~~ female genital mutilation/cutting before introducing measures to eliminate ~~FGM/C~~ it in the traditional communities. This may include having non-confrontational dialogue between all stakeholders, for understanding of the dangers and benefits of ~~FGM/C~~ female genital mutilation/cutting to the nation which can be shared from everyone for best solutions to be found.

Key Words: Replace Community Readiness Tool, ~~Female Genital Mutilation/Cutting,~~ Intergenerational ~~FGM/C~~ Female Genital Mutilation/Cutting, Traditional Birth Attendant

~~**Data Availability Statement:** This is a qualitative study that involves 8 traditional birth attendants who agreed to participate in this study based on anonymity that all information provided will be strictly confidential. All transcripts were anonymized including the extracts that support the study's findings. Transcripts are available for research purposes only in line with the approved research protocol. This is in fulfilment of the participants confidentiality as indicated in the approved research protocol from the Humanities and Social Sciences Research Ethics Committee [HSSREC/00000924/2019] and Osun state Ministry of Health [OSHREC/PRS/569T/157] dated 20th April 2020 and 19th August 2019 respectively.~~

~~**Funding:** The corresponding author received student scholarship from the University of KwaZulu-Natal, South Africa through the College of Health Sciences student Scholarship.~~

~~**Competing interests:** The authors have no competing interests of any form as regards this study.~~

Introduction

Female genital mutilation/cutting (FGM/C) is a public health problem globally. It comprises all procedures that involve partial or total removal of the external female genitalia, or other injury to the female genital organs for non-medical reasons. It is also referred to as multiple procedures on the female genital organs for non-medical reasons [1]. FGM/C has been described as a harmful practice that violates the sexual and reproductive health rights of women and girls [2,3]. It is estimated that more than 200 million girls and women alive today have undergone FGM/C across 30 countries including the Middle East, Asia, and Africa [4]. Of this, about 44 million are girls under the age of 15 years [5]. It is further projected that more than 3 million girls and women are currently at risk of FGM/C worldwide [4]. The current estimates of 3.9 million girls mutilated annually will also rise to 4.6 million by 2030 because there is an increase in the population growth in the communities that still practice FGM/C [6]. It is reported that 20 million girls and women are estimated to have undergone FGM/C in Nigeria, with Osun state, where the study was conducted, having reached the highest prevalence of 76.6% of FGM/C [7].

In Nigeria, ~~female genital mutilation/cutting (~~FGM/C~~)~~ is a deeply entrenched custom that is commonly performed by the traditional birth attendants (TBAs) who claim it as a heritage which confers honor on a woman and her family, a rite of passage to womanhood, preservation of chastity, enhancement of fertility, and prevention of death of the child during delivery among its benefits [8].

Despite its cultural importance, FGM/C has received considerable criticism worldwide because of its potential risk for various forms of health complications [9]. The short term and physical health complication of FGM/C includes but are not limited to pain, risk of

hemorrhage, shock, tetanus/sepsis, inability to urinate, damage to adjacent organs such as the rectum and urinary tract, dislocation and fracture of the pelvic bones and the head of the femur due to struggle while being restrained, open sore in the genital region, risk of HIV/AIDS or Hepatitis B. While the long term and reproductive health consequences of FGM/C include difficulty in passing menstrual blood, vaginal fistula, painful sexual intercourse, and difficulty in passing urine which can lead to recurrent urinary tract infection, clitorial cyst, infertility, an increased risk of childbirth complication and newborn death, need for future surgeries to allow for sexual intercourse and childbirth, psychological trauma, and risk of death from hemorrhage or infection [10].

Furthermore, sexuality is an equal right that both males and females must benefit from. While men are being circumcised for health reasons, women and girls are cut deliberately to remove their sexual feelings and subjugate them to the dictates of their men counterpart and it is done without any consent from them.

In 2005, the Violence Against Persons (Prohibition) Act 2015 was passed to eliminate the FGM/C practice in Nigeria. Since then, several legal, community based, and health-based strategies had been instituted to eliminate the FGM/C practice [~~9~~11]. These strategies were met with strong opposition from the TBAs, who remain the self-proclaimed custodians of the traditional responsibility of FGM/C from time immemorial, especially in Osun State, Nigeria. The TBAs believe in the supremacy of FGM/C as the traditional heritage, which is concerned about the wellbeing of the people, that it predates modern medicine and therefore should not be stopped. This cultural belief has facilitated the intergenerational preservation of FGM/C and explains their attitude towards the elimination of its practice. The intergenerational preservation of FGM/C is reflected on most women's belief that the elimination of FGM/C is tantamount to defaulting from their religion or traditions which could breed calamity for them and their offspring. These beliefs have been passed down from one generation to the other

and contribute to the high prevalence of FGM/C in Osun State, Nigeria, where it has become major public health issue that requires public health approach-based prevention interventions. Because of their influence on the intergenerational preservation of FGM/C, we identified the TBAs as community leaders who are key towards the development and implementation of public health prevention intervention for FGM/C in Osun State, Nigeria. The purpose of the study was to ~~determine and describe~~assess the attitude~~s~~ of the TBAs as community leaders towards the elimination of intergenerational FGM/C of girls in Osun State, Nigeria, in order to determine their level of readiness and preparedness towards achieving its elimination.

Materials and Methods

Study setting

Osun State is an inland state in South-West Nigeria with a population of 3,423,535 [12]. The present Osun State was created in 1991 from the old Oyo State. The state shares boundary with Kwara State in the North, Oyo in the West, Ogun and Ondo in the South and Ekiti State on East. The state capital is Osogbo, which is a local government area on its own. In all, a total of thirty local government council areas and Ife east area council constitute the state. Among the major towns in the state are Ilesha, Ife, Ede, Ikirun, Ikire, Ejigbo and Osogbo [13]. Before this time, the state was part of the old Oyo State. Osun State experiences a tropical climate while the local vegetation is the lowland rainforest type.

Indigenes of Osun State belong to the Yoruba race and comprise the Oyo, Ife, Ijesa, Igbomina and Osun. Many non-indigenes and foreigners also reside in the state and live together in harmony. Yoruba and English are the languages used for official and business transactions. The people have a rich cultural heritage that is demonstrated in all spheres of their life including the performance of FGM/C. Their culture finds expression in their arts, literature, music, and other social activities.

This study was conducted among the Yoruba TBAs residing across the three senatorial districts in Osun State, Nigeria. The Yoruba people have a rich cultural heritage that is

demonstrated in all spheres of their life including the performance of FGM/C in the Osun State, Nigeria.

Research design

A qualitative research design, using the adapted REPLACE community readiness to end FGM/C interview guide [104] was used to ~~determine and describe~~assess the attitudes of the TBAs as community leaders towards elimination of intergenerational FGM/C to determine their level of readiness and preparedness towards achieving its elimination in Osun State, Nigeria. This was the most suitable approach because it focuses on social and cultural contexts to understand how a problem is perceived and whether taking some type of action is seen as needed by community leaders to change the problem [1511, 12]16]. The adapted REPLACE community readiness to end FGM/C interview guide [104] was translated to the local (Yoruba) language by a bilingual expert in Yoruba and English who is also a qualitative researcher for comparison of the language translation to avoid the loss of meaning in the translated interview guides. The same person was used for translating transcripts to English.

Sampling and recruitment of participants

Purposive sampling method was used select eight TBAs whom we were regarded as community leaders and key informants with rich information regarding intergenerational FGM/C in Osun State, Nigeria. The inclusion criteria for recruitment and participation in the study included only TBAs who were formally recognized as leaders in performing FGM/C, who had been previously involved in any of the Government’s organized seminars or workshops on FGM/C elimination and or prevention intervention in Osun State, irrespective of the duration of the practice. The selection and recruitment of the participants commenced

once the ethical approval from the Humanities and Social Sciences Research Ethics Committee [HSSREC/00000924/2019] and Osun state Ministry of Health [OSHREC/PRS/569T/157] was obtained. Meetings were held with the nurses in the maternity units across the three senatorial districts as gatekeepers to inform them about the purpose of the study and to seek their permission to meet the TBAs working in their respective Units. A meeting with TBAs was held where the purpose of the study, the research eligibility criteria and request for voluntary participation from those who meet the inclusion criteria were discussed. Those who showed interest were recruited and were given the information sheet and the informed consent form to complete and sign. Arrangements for the interview session were then made by allowing the potential participants to choose suitable date and time within the first 7 days after the participants have given their consent. Before the commencement of the interview, the purpose of the study, and permission to audio record the interview session were explained again to the participants in the local language (Yoruba). A total sample size of eight traditional birth attendants as community leaders and key informants were recruited in the study. This small sample size is in line with the REPLACE community readiness tool, which suggests 6-12 key informants as sufficient to address issues in the community [1~~0~~4].

Pilot study

The first two TBAs out of the sample size were used for pilot study to ensure that the interview guide questions were easy and yielded the necessary data. The data generated from the pilot study were included in the main study data analysis. Contamination of data was not considered to be a concern because in qualitative studies data collection is progressive and the data from pilot studies can be used for the main study [1~~3~~7,1~~4~~8].

Data collection

Data collection and data analysis were conducted concurrently between October 2020 and March 2021. All COVID-19 precaution protocols like using the face mask, maintaining social distancing and hand sanitizing were observed during the interviews. In-depth interviews were conducted with the individual TBAs in their homes by the first author in Yoruba, the local language, using the interview guide adapted from the REPLACE Community Readiness to End FGM/C Assessment tool [1~~0~~4]. The interviews were audio recorded and transcribed within 24 hours of recording when information was still fresh in the researcher's memory for data analysis purposes. Each interview lasted about 60 minutes to complete thus allowing each TBA to adequately share their views as attitudes towards elimination of intergenerational FGM/C in Osun state. Nigeria. The transcripts were stored in word document formats on a computer with encrypted password and was backed up to an external hard drive to prevent data loss in line with the University's principles for data storage.

Data analysis

The six steps, namely: familiarization, coding, searching for themes, reviewing themes, defining, and naming themes, and writing up as described by [1~~5~~9] were used to analyze the transcribed data.

The first author went through the transcripts reading and re-reading them to familiarise herself and make sense of the data. Notes were taken and preliminary ideas for codes were marked. In order to start organizing the data into meaningful groups, codes were assigned to briefly describe something interesting that was said by the participants. The first author

grouped the newly generated codes with same meaning together to form themes. The second author, who also doubled as the inter coder at this stage, also did the same thing. The generated themes by the first and second authors were scrutinized by both of them to ensure that they were useful and accurate representation of the data by discarding some themes and creating new ones from the transcribed data. The first author then formulated the meaning of each theme so that the data can be understood. At this stage, appropriate naming of themes was made to succinctly interpret the data collected.

Ethical Permission

All ethical principles were adhered to including, consent form for voluntary participation, ethical approval from the Humanities and Social Sciences Research Ethics Committee [HSSREC/00000924/2019] and Osun state Ministry of Health [OSHREC/PRS/569T/157] were obtained.

The names assigned to each of the participants are not their actual names as we are aware of the ethical implication. However, using pseudonyms instead of participants' actual names ensured confidentiality and anonymity.

Measure to ensure trustworthiness

Measures to ensure trustworthiness and rigor of the findings were applied through the criteria of credibility, confirmability, dependability, and transferability [~~17~~20].

To ensure credibility, comprehensive field notes, noting gesture and other non-verbal ques observed during the interviews were recorded and analyzed. Audit trail of all research

processes were recorded. Analysis of the data gathered reflected a neutral interpretation of the data rather than the researcher's view as recommended by [~~18~~21,~~19~~22]. This was achieved by conducting a member checking on data whereby the analyzed data was summarized and provided to the participants for them to validate and determine the accuracy of the data as their views. For dependability and transferability, an audit trail was kept and the comprehensive and rich details of the study setting, and the socio-demographic characteristics of the participants are presented. To further establish trust and confidence in the findings, the authors compared their individually generated themes from the raw data to ensure that they were useful and accurate representation of the data by discarding some themes and creating new ones from the transcribed data. The senior author and research supervisor reviewed the themes against raw data and helped in refining these to reflect participants' views to enhance confirmability. Common issues recur and main themes were identified to summarize all the views that have been captured in the data. One theme and three subthemes emerged that revealed the defensive attitude of the TBAs towards the elimination of intergenerational FGM/C of girls in Osun state, Nigeria.

Results

Eight (8) TBAs, comprising of three females and five males participated in the study on TBA’s attitude towards the elimination of intergenerational FGM/C of girls in Osun State, Nigeria.

Table 1: Participants’ Demographic Characteristics

Participant	Age	Gender	Religion	Educational level
Lukman	45	Male	Islam	Tertiary education
Abike	70	Female	Islam	Primary education
Adeyemi	53	Male	Islam	Tertiary education
Olalekan	86	Male	Islam	Primary education
Mojirola	63	Female	Christian	Secondary education
Amope	80	Female	Islam	Primary education
Fadipe	73	Male	Islam	Secondary education
Adebayo	75	Male	Islam	Primary education

These demographics reflects the TBAs in Osun State where the person who becomes a TBA is culturally determined through the lineage of the traditional circumcisers. It is a taboo for a woman who is married to perform FGM/C as TBA, but she can still retain her knives for performing FGM/C on her own children only (personal conversation with Mr Adeyemi, a male participant to the study, December 2020). This explains why ~~the majority of~~most

participants were male TBAs in the study as reflected in Table 1. Their ages ranged between 45 and 86 years. Seven of them practiced Islam and only one was Christian as a religion.

Defensive attitude towards elimination of intergenerational FGM/C

Analyzed data yielded "Defensive attitude towards elimination of FGM/C" as the main theme in the TBAs' attitude towards the elimination of intergenerational FGM/C of girls in Osun State, Nigeria. Three subthemes, including (i) FGM/C as a traditional heritage, (ii) Defiance against government's efforts to eliminate intergenerational FGM/C and Debunking all "western" information about dangers of FGM/C as lies emerged under the main theme.

FGM/C as traditional heritage

The FGM/C as a traditional heritage sub theme emerged from data related TBAs' defensive attitudes towards elimination of intergenerational FGM/C as traditional and cultural beliefs that the practice is the identity and the only link between the current and past among generations as described in the following extracts:

I am talking about our traditional heritage here. It can't be stopped; it can't stop now. The law, civilization coupled with ignorance is stopping people, but it cannot be stopped. [Lukman, Male, Age: 45, Tertiary Education]

I think all they are saying about stopping of our tradition is done out of civilization and hatred, hatred of us and our traditional heritage. What has been established by our forefathers cannot perish. It is just like asking us to abandon our language because of

civilization. There is no where we meet that we can do without speaking our language. Girl child circumcision cannot perish. [Abike, Female, Age:70, Primary Education]

Further data under this subtheme showed that TBAs consider it as their primary responsibility to protect intergenerational FGM/C practice from extinction as demonstrated in the following extracts:

Girl circumcision has been in existence from time immemorial. Therefore, it can be described as our cultural identity that was transmitted from generation to generation that no one is ready to allow it to die down in his or her own time, even if your government says it is bad for us. [Fadipe, Male, Age:73, Secondary Education]

But you know, it is our heritage which must be promoted. It is not that it really makes more money for us. Do you understand me? It is not what the TBAs are eating from. But it is what was handed over to us as our heritage and must not be taken away just like that! [Abike, Female, Age: 70, Primary Education].

Defiance against government's efforts to eliminate FGM/C

This subtheme emerged from data related to the defiant attitudes of the TBAs towards seminars and workshops that had been previously arranged by government and other organizations in the country with the aim of highlighting the disadvantages of intergenerational FGM/C proposals towards eliminating FGM/C as described in the following extracts:

I attended the workshops about this, but I was and am still convinced that the disadvantages of government's effort to stopping FGM/C will be enormous for our women. See what is happening nowadays in our communities. No one will be able to control these girls. We must

fight anyone including the government to protect our girls from promiscuity. [Lukman, Male, Age: 45, Tertiary Education]

No, I don't agree with the government's decision. The result will be very bad. As for me, I did it for all my children both boys and girls. None of my children are promiscuous because I circumcised them and took care of them all. I buried what was cut and properly watered the soil where I buried it so that chicken could not be able to unearth it. Otherwise, if unearthed, the girl child will be promiscuous. We are very careful about these things so that no one can come back and say FGM/C doesn't work. [Amope, Female, Age: 80, Primary Education].

Ah! I am not in support of the government stopping the girl child circumcision. That is our campaign, and I am not hiding it. At least, it will not happen in my time. [Abike, Female, Age 70, Primary Education]

Personally, I don't agree with government's reasons to stop girl child circumcision. I have said it earlier that, based on my experience and the level of my education, and coming from a family that is traditionally practicing FGM/C, I do not see it being stopped by anyone, even the government or anyone else, not now. I did it (FGM/C) for my girls and there is no problem associated with it, I don't think it should be stopped. It will be a great disadvantage if anyone could agree with government. [Lukman, Male, Age: 45, Tertiary Education].

Debunking all "Western" information about dangers of FGM/C to women as lies

This subtheme emerged from data related to TBAs efforts in debunking most of the information and claims about the benefits of eliminating intergenerational FGM/C as just

government's efforts to brainwash people, against the practice of FGM/C as depicted in the quotes below.

The girls that I circumcised did not have any problem. Even when grown up, these girls do not develop any of the problems we were told resulted from FGM/C, which we were now told can be associated with FGM/C. The girls are now big women and are all alive and doing well in their families. They got married to nice men and have lively children. [Amope, Female, Age 80, Primary Education]

Every girl that we have circumcised is still alive even today. No child can die in the hand of circumcision surgeon and all our girls were circumcised. Anyone who say we put our girls in danger is a liar. My wife is circumcised, all my girls are circumcised and alive. They must stop spreading lies about FGM/C. [Olalekan, Male, Age 86, Primary Education]

The likelihood of lack of enjoyment during sex was further debunked but rather held that circumcised women enjoy sex more as demonstrated in the extract below.

That debunked FGM/C leads to women not enjoying sex is one of the lies the government is peddling about girl circumcision. But they have forgotten that it's only a small part of the clitoris that is cut and not the whole clitoris. it is not true at all. [Adeyemi, Male, Age 53, Tertiary Education]

It is only a false assumption that circumcised women do not enjoy sex. When a circumcised woman has sexual intercourse with men, she will glue to him as if they tie penis and vagina together, in that way, they both enjoy each other nicely [Olalekan, Male Age: 86, Primary Education]

Furthermore, the views that FGM/C spreads sexually transmitted infections has been refuted by the TBAs as just rumors as captured in the following extracts:

There is nothing like transmission of sexual diseases by FGMC, bleeding and all so many other things that are being said about female circumcision. It is safe and cultural to do it (circumcision). [Mojirola, Female, Age 63, Secondary Education]

We were also told that female circumcision causes sexual transmitted diseases / infections which is not true at all! Honestly, I don't know what the government want to gain from spreading such lies for stopping it. Female circumcision does not have any negative side effects as it is being peddled around. [Amope, Female, Age 80, Primary Education].

Discussion

The purpose of the study was to ~~explore and describe~~assess TBAs' attitudes towards the elimination of intergenerational FGM/C of girls in Osun state, Nigeria in order to determine their level of readiness and preparedness towards achieving its elimination.

~~Several efforts have been put in place by the government to facilitate the elimination of intergenerational FGM/C in Osun State, Nigeria. These include the promulgation of laws to sanction the erring perpetrators, organized sensitization programs in form of workshops to enlighten the TBAs on the consequences of the practice and the need for it to be stopped. However, our findings reveal that all these are in vain as TBAs are adamant in their defiance against government's efforts to eliminate intergenerational FGM/C in Osun State, Nigeria.~~

Our findings show a deep defensive attitude towards elimination of FGM/C ~~as~~ among the TBAs as community leaders and custodians of the intergenerational FGM/C which is reflected in the participants .~~-~~views that FGM/C is their traditional heritage and its elimination can be equated to eliminating their cultural identity. Dunn concluded that the cultural identity of the Africans is being eliminated by current civilization [23] and Obi, Adesoye, Labiran also reported that, culture/tradition is the greatest human inheritance, since

it contributes to the preservation of history and identity of any race, thus confirming the TBAs' beliefs about their responsibility to preserve intergenerational FGM/C as their traditional heritage despite all its disadvantages to women, their families, and communities [24].

Findings from this study also depicted This was coupled by a strong defiance against the government's prevention interventions as indicated in the defiance shown by those TBAs who had attended the seminars and workshops organised by the Ggovernment on the elimination of intergenerational FGM/C, thus contrasting previous findings by [205] who concluded that that unsuccessful elimination of intergenerational FGM/C was due the government's inability to involve the major community stakeholders such as the TBAs [20].

Therefore, due to the TBAs' defiance against governments' efforts to eliminate FGM/C, there is a highlights that continuous non-alignment between government agencies and the views and beliefs of those responsible for promotion of historic traditions (such as FGM/C) always ending up in disagreement, if not properly handled. Giving the TBAs' voices and understanding their attitudes towards abandoning their age long custom, and not only inviting them to attend seminars or workshops on elimination of FGM/C is important in determining the community's readiness for development and implementation of interventions for eliminating intergenerational FGM/C of girls.

The participants' views that FGM/C is their traditional heritage and equating its elimination to the government's strategies to eliminate their cultural identity has been reported before. Dunn concluded that the cultural identity of the Africans is being eliminated by current civilization [21] and Obi, Adesoye, Labiran also reported that, culture/tradition is the greatest human inheritance, since it contributes to the preservation of history and identity of any race,

~~thus confirming the TBAs' beliefs about their responsibility to preserve intergenerational FGM/C as their traditional heritage despite all its disadvantages to women, their families, and communities [22].~~

The findings about debunking most of the information and claims about the benefits of eliminating FGM/C as just government's efforts to brainwash people is disputed by previous report which supports the government's assertion on the benefits of eliminating intergenerational FGM/C. Ibrahim, Oyeyemi and Ekine ~~[23]~~ reported that more patients with complications of FGM/C such as difficult delivery, perineal laceration, hemorrhage, scar/keloid etc. were seen by nurses/midwives than doctors in Yenegoa, Bayelsa, Nigeria [26].

The TBAs are of the opinion that the involvement of people who are not traditionally entitled to circumcision and are not knowledgeable enough are the ones causing the complications as stated by the government. The TBAs' debunking of all "Western" information about dangers of FGM/C to women as lies may be due to their lack of knowledge of the dangers and complications of FGM/C. Similar findings were reported by [26] who concluded limited knowledge about complications may be reflected as negative attitudes towards prevention or elimination of intergenerational FGM/C in their lifetime.

Our findings show that FGM/C is going to continue in Osun state, Nigeria despite government's efforts to eliminate intergenerational FGM/C. This is confirming the report by the UNFPA which estimated that because of population growth, additional 4.6 million girls are projected to be at risk of undergoing female genital mutilation by 2030 [2~~4~~7]. It was also reported that the outbreak of COVID-19 between 2020 and 2022 aggravated the vulnerability

of girls and women who are at risk of FGM/C which indicated that 2 million cases of FGM/C could be recorded over the next decade due to COVID-19 alone [2~~4~~7].

~~The TBAs' debunking of all "Western" information about dangers of FGM/C to women as lies may be due to their lack of knowledge of the dangers and complications of FGM/C. Similar findings were reported by [22] who concluded limited knowledge about complications may be reflected as negative attitudes towards prevention or elimination of intergenerational FGM/C in their lifetime.~~

Conclusion

The current defensive attitudes of the TBAs as community leaders are worrisome because they are an indication of the community leaders' level of no readiness for intervention~~s~~ programmes for the elimination of intergenerational FGM/C in Osun state, Nigeria. ~~It is an indication of the level of no readiness for intervention programmes for the elimination of intergenerational FGM/C in the state.~~ We recommend that the government, policy makers and health professionals elicit and carefully consider the views of the community leaders such as TBAs who are custodians of the FGM/C before introducing measures to eliminate FGM/C in traditional communities. This may include having non-confrontational dialogue between all stakeholders~~,~~ for understanding of the dangers and benefits of FGM/C to the nation which can be shared from everyone for best solutions to be found.

Acknowledgement

Our sincere appreciation goes to the management of the Ministry of Health in Osun state Nigeria and University of KwaZulu-Natal for granting us the ethical approval to carry out the study. We also thank the Nurses and the Traditional Birth Attendants for creating time to grant the interviews.

Data Availability Statement: This is a qualitative study that involves 8 traditional birth attendants who agreed to participate in this study based on anonymity that all information provided will be strictly confidential. All transcripts were anonymized including the extracts that support the study's findings. Transcripts are available for research purposes only in line with the approved research protocol. This is in fulfilment of the participants confidentiality as indicated in the approved research protocol from the Humanities and Social Sciences Research Ethics Committee [HSSREC/00000924/2019] and Osun state Ministry of Health [OSHREC/PRS/569T/157] dated 20th April 2020 and 19th August 2019 respectively.

Funding: The corresponding author received student scholarship from the University of KwaZulu-Natal, South Africa through the College of Health Sciences student Scholarship.

Competing interests: The authors have no competing interests of any form as regards this study.

References

1. World Health Organization. Female Genital Mutilation. Fact sheet. 2020 Retrieved February 20, 2022, from http://www.who.int/mediacentre/factsheets/fs241/en/
2. Waigwa S, Doos L, Bradbury-Jones, Taylor J. "Effectiveness of health education as an intervention designed to prevent female genital mutilation/cutting (FGM/C): a systematic review." Reproductive health 2018; **15**(1): 62.
3. United Nations, 2015. ‘The Millennium Development Goals Report 2015’, pg. 38. http://www.un.org/millenniumgoals/2015_MDG_Report/pdf/MDG%202015
4. UNICEF. Female Genital Mutilation/Cutting: A Global Concern UNICEF, New York, 2016.
5. World Health Organization. Female genital mutilation: Key facts. 2018 [cited 1 May 2022]. https://www.who.int/news-room/fact-sheets/detail/female-genital-mutilation
6. UNFPA-UNICEF Joint Programme on the Elimination of Female Genital Mutilation (2019).
7. Nigeria Demographic and Health Survey (2013). National Population Commission. Federal Republic of Nigeria.
8. Berg RC, Denison E. Health Care for Women International A Tradition in Transition: Factors Perpetuating and Hindering the Continuance of Female Genital Mutilation / Cutting (FGM / C) Summarized in a Systematic Review A Tradition in Transition: Factors Perpetuating Genital. 2013;9332(October 2017).
9. Yaya, S. and B. Ghose (2018). "Female Genital Mutilation in Nigeria: A Persisting Challenge for Women’s Rights." Social Sciences 7(12): 244.
8.10. Reisel, D. and S. M. Creighton (2015). "Long term health consequences of Female Genital Mutilation (FGM)." Maturitas 80(1): 48-51.
11. Violence against Persons (Prohibition) Act,2015, Laws of the Federation of Nigeria

Formatted: English (United States)

12. National Population Commission of Nigeria. Nigeria Population and Housing Census 2006. Abuja, Nigeria: National Population Commission of Nigeria.

~~9.~~13. Omole, F.K., Lukman, Y. and Baki, A.I. (2014), "Analysis of market typology and functions in the development of Osun state, Nigeria", International Journal of Development and Sustainability, Vol. 3 No. 1, pp. 55-69

14. Barret H. Alhassan Y. The REPLACE Community Readiness to End FGM Assessment. Practical Handbook (based on REPLACE Approach www.replacefgm2.eu) 2016, Coventry university, UK.

~~10.~~15. McCoy H.V, Malow R., Edwards R.W., Thurland A., Rosenberg R. A Strategy for Improving Community Effectiveness of HIV/AIDS Intervention Design: The Community Readiness Model in the Caribbean, Substance Use & Misuse, 2007; 42:10, 1579-1592, DOI: 10.1080/10826080701212535

~~11.~~16. Borrayo, E. A. (2007). "Using a community readiness model to help overcome breast health disparities among US Latinas." Substance use & misuse **42**(4): 603-619.

~~12.~~17. Duma, S. Women's journey of recovery from sexual assault trauma: a grounded theory.2006.

~~13.~~18. Van Teijlingen, E R, Hundley V. "The importance of pilot studies." (2001).

~~14.~~19. Braun V, Clarke V. Thematic analysis. in H. Cooper, P. M. Camic, D. L. Long, A. T. Panter, D. Rindskopf, & K. J. Sher (Eds.), *APA handbook of research methods in psychology*, *2012;Vol. 2. Research designs: Quantitative, qualitative, neuropsychological, and biological* (pp. 57–71). American Psychological Association. https://doi.org/10.1037/13620-004

~~15. Dickert N, Emanuel E, Grady Christine. "Paying research subjects: an analysis of current policies." Annals of Internal Medicine 2002; **136**(5): 368-373.~~

Formatted: Font: (Default) Times New Roman, 12 pt

Formatted: Font: Font color: Auto, English (United States)

~~16.~~20. Louis Cohen L, Manion L, Morrison K. Action research. Research methods in education, Routledge 2013: 368-385.

~~17.~~21. Polit, D. and C. Beck. "Essentials of nursing research." Ethics 2012; **23**(2).

~~18.~~22. Fawcett J, Garity J. "Chapter 1: Research and evidence-based nursing practice." Evaluating research for evidence-based nursing: (2009); 3-20.

~~19.~~1. ~~Andarge, Masresha Yazew. *The difficulties of ending Female Genital Mutilation (FGM): Case of Afar pastoralist communities in Ethiopia*. Social Policy for Development (SPD) (2014, December 12). Retrieved from http://hdl.handle.net/2105/17487~~

~~20.~~23. Dunn F. Is It Possible to End Female Circumcision in Africa? Clin J Obstet Gynecol. 2021;1: 007-013. https://doi.org/10.29328/journal.cjog.1001002

24. Obi A.I., Adesoye O., Labiran A. Female Genital Mutilation: Attitude and Practices among Women in Okada Community, Edo State JMBR: A Peer-review Journal of Biomedical Sciences December 2015, Vol. 14 No. 2 pp 138-150

25. Andarge, Masresha Yazew. *The difficulties of ending Female Genital Mutilation (FGM): Case of Afar pastoralist communities in Ethiopia*. Social Policy for Development (SPD) (2014, December 12). Retrieved from http://hdl.handle.net/2105/17487

~~21.~~

~~22.~~26. Ibrahim I.A, Oyeyemi A.S, Ekine A.A. Knowledge, Attitude and Practice of Female Genital Mutilation among Doctors and Nurses in Bayelsa state, Niger-Delta of Nigeria. International Journal of Medicine and Biomedical Research Volume 2 Issue 1 January – April 2013

Formatted: English (United States)

Formatted: Indent: Left: 0.5", No bullets or

27. UNFPA. 2020. ‘Impact of the COVID-19 Pandemic on Family Planning and Ending Gender-Based Violence, Female Genital Mutilation and Child Marriage.’ Interim Technical Note.

Formatted: English (United States)

Formatted: Font: (Default) Times New Roman, 12 pt

Full Title:

'It Is What Was Handed Over to Us as Our Heritage and Must Not Be Taken Away Just Like That': Traditional Birth Attendants' Attitudes Towards the Elimination of Intergenerational Female Genital Mutilation/Cutting in Osun State, Nigeria.

Short Title:

Eliminating Intergenerational Female Genital Mutilation/Cutting in Osun State, Nigeria.

Authors:

ROSEMARY OMOLARA FAFOWORA[1]¶*ID orcid.org/0000-0003-2968-3784

SINEGUGU EVIDENCE DUMA[2]¶ID orcid.org/0000-0002-2489-8770

[1]Department of Nursing, School of Nursing and Public Health, College of Health Sciences, Howard Campus, University of KwaZulu-Natal, Durban, South Africa.

[2]Department of Nursing, School of Nursing and Public Health, College of Health Sciences, Howard Campus, University of KwaZulu-Natal, Durban, South Africa.

*Corresponding author

E-mail: rosemary.olagunju@gmail.com (RO)

¶These authors equally contributed to this work.

Abstract

Background

Female genital mutilation/cutting is a harmful practice that violates the sexual and reproductive health rights of women and girls. The practice is often perpetrated directly or indirectly from one generation to another as a way of preserving culture, thus making it difficult to tackle using ordinary prevention interventions. The purpose of the study was to assess the attitude of the traditional birth attendants as community leaders towards the elimination of intergenerational female genital mutilation/cutting and to determine their level of readiness and preparedness towards achieving it in Osun State, Nigeria.

Methodology/Results

A qualitative research design, using the adapted REPLACE community readiness tool to end female genital mutilation/cutting interview guide, was used to individually interview eight traditional birth attendants who were identified through purposive sampling method as community leaders and key informants. Thematic Analysis was used to analyze the data which yielded female genital mutilation/cutting as traditional heritage, defiance against government efforts and debunking all “western” information about dangers of female genital mutilation to women as lies as findings. These findings are worrisome because they are an indication of the community leaders’ level of no readiness for interventions for elimination of intergenerational female genital mutilation/cutting in Osun state, Nigeria.

Conclusion

We recommend that the government, policy makers and health professionals elicit and carefully consider the views of the community leaders such as traditional birth attendants who are custodians of the female genital mutilation/cutting before introducing measures to

eliminate it in the traditional communities. This may include having non-confrontational dialogue between all stakeholders, for understanding of the dangers and benefits of female genital mutilation/cutting to the nation which can be shared from everyone for best solutions to be found.

Key Words: Replace Community Readiness Tool, Intergenerational Female Genital Mutilation/Cutting, Traditional Birth Attendant

Introduction

Female genital mutilation/cutting (FGM/C) is a public health problem globally. It comprises all procedures that involve partial or total removal of the external female genitalia, or other injury to the female genital organs for non-medical reasons. It is also referred to as multiple procedures on the female genital organs for non-medical reasons [1]. FGM/C has been described as a harmful practice that violates the sexual and reproductive health rights of women and girls [2,3]. It is estimated that more than 200 million girls and women alive today have undergone FGM/C across 30 countries including the Middle East, Asia, and Africa [4]. Of this, about 44 million are girls under the age of 15 years [5]. It is further projected that more than 3 million girls and women are currently at risk of FGM/C worldwide [4]. The current estimates of 3.9 million girls mutilated annually will also rise to 4.6 million by 2030 because there is an increase in the population growth in the communities that still practice FGM/C [6]. It is reported that 20 million girls and women are estimated to have undergone FGM/C in Nigeria, with Osun state, where the study was conducted, having reached the highest prevalence of 76.6% of FGM/C [7].

In Nigeria, FGM/C is a deeply entrenched custom that is commonly performed by the traditional birth attendants (TBAs) who claim it as a heritage which confers honor on a woman and her family, a rite of passage to womanhood, preservation of chastity, enhancement of fertility, and prevention of death of the child during delivery among its benefits [8].

Despite its cultural importance, FGM/C has received considerable criticism worldwide because of its potential risk for various forms of health complications [9]. The short term and physical health complication of FGM/C includes but are not limited to pain, risk of hemorrhage, shock, tetanus/sepsis, inability to urinate, damage to adjacent organs such as the rectum and urinary tract, dislocation and fracture of the pelvic bones and the head of the femur due to struggle while being restrained, open sore in the genital region, risk of HIV/AIDS or Hepatitis B. While the long term and reproductive health consequences of FGM/C include difficulty in passing menstrual blood, vaginal fistula, painful sexual intercourse, and difficulty in passing urine which can lead to recurrent urinary tract infection, clitoral cyst, infertility, an increased risk of childbirth complication and newborn death, need for future surgeries to allow for sexual intercourse and childbirth, psychological trauma, and risk of death from hemorrhage or infection [10].

Furthermore, sexuality is an equal right that both males and females must benefit from. While men are being circumcised for health and religious ~~,~~reasons, women and girls are cut deliberately to remove their sexual feelings and subjugate them to the dictates of their men counterpart, and it is done without any consent from them.

In 2005, the Violence Against Persons (Prohibition) Act 2015 was passed to eliminate the FGM/C practice in Nigeria. Since then, several legal, community based, and health-based strategies had been instituted to eliminate the FGM/C practice [11]. These strategies were met

with strong opposition from the TBAs, who remain the self-proclaimed custodians of the traditional responsibility of FGM/C from time immemorial, especially in Osun State, Nigeria. The TBAs believe in the supremacy of FGM/C as the traditional heritage, which is concerned about the wellbeing of the people, that it predates modern medicine and therefore should not be stopped. This cultural belief has facilitated the intergenerational preservation of FGM/C and explains their attitude towards the elimination of its practice. The intergenerational preservation of FGM/C is reflected on most women's belief that the elimination of FGM/C is tantamount to defaulting from their religion or traditions which could breed calamity for them and their offspring. These beliefs have been passed down from one generation to the other and contribute to the high prevalence of FGM/C in Osun State, Nigeria, where it has become major public health issue that requires public health approach-based prevention interventions. Because of their influence on the intergenerational preservation of FGM/C, we identified the TBAs as community leaders who are key towards the development and implementation of public health prevention intervention for FGM/C in Osun State, Nigeria. The purpose of the study was to assess the attitude of the TBAs as community leaders towards the elimination of intergenerational FGM/C of girls in Osun State, Nigeria, in order to determine their level of readiness and preparedness towards achieving its elimination.

Materials and Methods

Study setting

Osun State is an inland state in South-West Nigeria with a population of 3,423,535 [12]. The present Osun State was created in 1991 from the old Oyo State. The state shares boundary with Kwara State in the North, Oyo in the West, Ogun and Ondo in the South and Ekiti State on East. The state capital is Osogbo, which is a local government area on its own. In all, a total of thirty local government council areas and Ife east area council constitute the state. Among the major towns in the state are Ilesha, Ife, Ede, Ikirun, Ikire, Ejigbo and Osogbo

[13]. Before this time, the state was part of the old Oyo State. Osun State experiences a tropical climate while the local vegetation is the lowland rainforest type.

Indigenes of Osun State belong to the Yoruba race and comprise the Oyo, Ife, Ijesa, Igbomina and Osun. Many non-indigenes and foreigners also reside in the state and live together in harmony. Yoruba and English are the languages used for official and business transactions. The people have a rich cultural heritage that is demonstrated in all spheres of their life including the performance of FGM/C. Their culture finds expression in their arts, literature, music, and other social activities.

This study was conducted among the Yoruba TBAs residing across the three senatorial districts in Osun State, Nigeria. The Yoruba people have a rich cultural heritage that is demonstrated in all spheres of their life including the performance of FGM/C in the Osun State, Nigeria.

Research design

A qualitative research design, using the adapted REPLACE community readiness to end FGM/C interview guide [14] was used to assess the attitudes of the TBAs as community leaders towards elimination of intergenerational FGM/C to determine their level of readiness and preparedness towards achieving its elimination in Osun State, Nigeria. This was the most suitable approach because it focuses on social and cultural contexts to understand how a problem is perceived and whether taking some type of action is seen as needed by community leaders to change the problem [15, 16]. The adapted REPLACE community readiness to end FGM/C interview guide [14] was translated to the local (Yoruba) language by a bilingual expert in Yoruba and English who is also a qualitative researcher for comparison of the language translation to avoid the loss of meaning in the translated interview guides. The same person was used for translating transcripts to English.

Formatted: Font color: Auto

Sampling and recruitment of participants

Purposive sampling method was used select eight TBAs whom we were regarded as community leaders and key informants with rich information regarding intergenerational FGM/C in Osun State, Nigeria. The inclusion criteria for recruitment and participation in the study included only TBAs who were formally recognized as leaders in performing FGM/C, who had been previously involved in any of the Government's organized seminars or workshops on FGM/C elimination and or prevention intervention in Osun State, irrespective of the duration of the practice. The selection and recruitment of the participants commenced once the ethical approval from the Humanities and Social Sciences Research Ethics Committee [HSSREC/00000924/2019] and Osun state Ministry of Health [OSHREC/PRS/569T/157] was obtained. Meetings were held with the nurses in the maternity units across the three senatorial districts as gatekeepers to inform them about the purpose of the study and to seek their permission to meet the TBAs working in their respective Units. A meeting with TBAs was held where the purpose of the study, the research eligibility criteria and request for voluntary participation from those who meet the inclusion criteria were discussed. Those who showed interest were recruited and were given the information sheet and the informed consent form to complete and sign. Arrangements for the interview session were then made by allowing the potential participants to choose suitable date and time within the first 7 days after the participants have given their consent. Before the commencement of the interview, the purpose of the study, and permission to audio record the interview session were explained again to the participants in the local language (Yoruba). A total sample size of eight traditional birth attendants as community leaders and key informants were recruited in the study. This small sample size is in line with the REPLACE community readiness tool, which suggests 6-12 key informants as sufficient to address issues in the community [14].

Pilot study

The first two TBAs out of the sample size were used for pilot study to ensure that the interview guide questions were easy and yielded the necessary data. The data generated from the pilot study were included in the main study data analysis. Contamination of data was not considered to be a concern because in qualitative studies data collection is progressive and the data from pilot studies can be used for the main study [17,18].

Data collection

Data collection and data analysis were conducted concurrently between October 2020 and March 2021. All COVID-19 precaution protocols like using the face mask, maintaining social distancing and hand sanitizing were observed during the interviews. In-depth interviews were conducted with the individual TBAs in their homes by the first author in Yoruba, the local language, using the interview guide adapted from the REPLACE Community Readiness to End FGM/C Assessment tool [14]. The interviews were audio recorded and transcribed within 24 hours of recording when information was still fresh in the researcher’s memory for data analysis purposes. Each interview lasted between 45-60 minutes to complete thus allowing each TBA to adequately share their views as attitudes towards elimination of intergenerational FGM/C in Osun state. Nigeria. The transcripts were stored in word document formats on a computer with encrypted password and was backed up to an external hard drive to prevent data loss in line with the University’s principles for data storage.

Formatted: Font color: Auto

Formatted: Font color: Auto

Data analysis

The six steps, namely: familiarization, coding, searching for themes, reviewing themes, defining, and naming themes, and writing up as described by [19] were used to analyze the transcribed data.

The first author went through the transcripts reading and re-reading them to familiarise herself and make sense of the data. Notes were taken and preliminary ideas for codes were marked. In order to start organizing the data into meaningful groups, codes were assigned to briefly describe something interesting that was said by the participants. The first author grouped the newly generated codes with same meaning together to form themes. The second author, who also doubled as the inter coder at this stage, also did the same thing. The generated themes by the first and second authors were scrutinized by both of them to ensure that they were useful and accurate representation of the data by discarding some themes and creating new ones from the transcribed data. The first author then formulated the meaning of each theme so that the data can be understood. At this stage, appropriate naming of themes was made to succinctly interpret the data collected.

Formatted: Font color: Auto

Ethical Permission

All ethical principles were adhered to including, consent form for voluntary participation, ethical approval from the Humanities and Social Sciences Research Ethics Committee [HSSREC/00000924/2019] and Osun state Ministry of Health [OSHREC/PRS/569T/157] were obtained.

The names assigned to each of the participants are not their actual names as we are aware of the ethical implication. However, using pseudonyms instead of participants' actual names ensured confidentiality and anonymity.

Measure to ensure trustworthiness

Measures to ensure trustworthiness and rigor of the findings were applied through the criteria of credibility, confirmability, dependability, and transferability [20].

To ensure credibility, comprehensive field notes, noting gesture and other non-verbal ques observed during the interviews were recorded and analyzed. Audit trail of all research processes were recorded. Analysis of the data gathered reflected a neutral interpretation of the data rather than the researcher's view as recommended by [21,22]. This was achieved by conducting a member checking on data whereby the analyzed data was summarized and provided to the participants for them to validate and determine the accuracy of the data as their views. For dependability and transferability, an audit trail was kept and the comprehensive and rich details of the study setting, and the socio-demographic characteristics of the participants are presented. To further establish trust and confidence in the findings, the authors compared their individually generated themes from the raw data to ensure that they were useful and accurate representation of the data by discarding some themes and creating new ones from the transcribed data. The senior author and research supervisor reviewed the themes against raw data and helped in refining these to reflect participants' views to enhance confirmability. Common issues recur and main themes were identified to summarize all the views that have been captured in the data. One theme and three subthemes emerged that revealed the defensive attitude of the TBAs towards the elimination of intergenerational FGM/C of girls in Osun state, Nigeria.

Results

Eight (8) TBAs, comprising of three females and five males participated in the study on TBA's attitude towards the elimination of intergenerational FGM/C of girls in Osun State, Nigeria.

Table 1: Participants' Demographic Characteristics

Participant	Age	Gender	Religion	Educational level
Lukman	45	Male	Islam	Tertiary education
Abike	70	Female	Islam	Primary education
Adeyemi	53	Male	Islam	Tertiary education
Olalekan	86	Male	Islam	Primary education
Mojirola	63	Female	Christian	Secondary education
Amope	80	Female	Islam	Primary education
Fadipe	73	Male	Islam	Secondary education
Adebayo	75	Male	Islam	Primary education

These demographics reflects the TBAs in Osun State where the person who becomes a TBA is culturally determined through the lineage of the traditional circumcisers. It is a taboo for a woman who is married to perform FGM/C as TBA, but she can still retain her knives for performing FGM/C on her own children only (personal conversation with Mr Adeyemi, a male participant to the study, December 2020). This explains why most participants were male TBAs in the study as reflected in Table 1. Their ages ranged between 45 and 86 years. Seven of them practiced Islam and only one was Christian as a religion.

Defensive attitude towards elimination of intergenerational FGM/C

Analyzed data yielded "Defensive attitude towards elimination of FGM/C" as the main theme in the TBAs' attitude towards the elimination of intergenerational FGM/C of girls in Osun State, Nigeria. Three subthemes, including (i) FGM/C as a traditional heritage, (ii) Defiance against government's efforts to eliminate intergenerational FGM/C and Debunking all "western" information about dangers of FGM/C as lies emerged under the main theme.

FGM/C as traditional heritage

The FGM/C as a traditional heritage sub theme emerged from data related TBAs' defensive attitudes towards elimination of intergenerational FGM/C as traditional and cultural beliefs that the practice is the identity and the only link between the current and past among generations as described in the following extracts.

The response from the participant below upheld the fact that FGM/C is their traditional heritage that cannot be stopped in their lifetime. ~~The response from the respondent below upheld the fact that FGMC is their traditional heritage that cannot be stopped in their lifetime.~~

Formatted: Font color: Auto

I am talking about our traditional heritage here. It can't be stopped; it can't stop now. The law, civilization coupled with ignorance is stopping people, but it cannot be stopped. [Lukman, Male, Age: 45, Tertiary Education]

Another respondent also mentioned that FGM/C is a traditional heritage that was handed down by their forefathers. ~~Another respondent also mentioned that FGMC practice is a traditional heritage that was handed down by their forefathers.~~

Formatted: Font color: Auto

I think all they are saying about stopping of our tradition is done out of civilization and hatred, hatred of us and our traditional heritage. What has been established by our forefathers cannot perish. It is just like asking us to abandon our language because of civilization. There is no where we meet that we can do without speaking our language. Girl child circumcision cannot perish. [Abike, Female, Age:70, Primary Education]

Further, data under this subtheme showed that TBAs considerate as their primary responsibility to prevent intergenerational practice of FGM/C from extinction as depicted in the following extract. ~~Further data under this subtheme showed that TBAs consider it as their primary responsibility to protect intergenerational FGM/C practice from extinction as demonstrated in the following extracts.~~

Girl circumcision has been in existence from time immemorial. Therefore, it can be described as our cultural identity that was transmitted from generation to generation that no one is ready to allow it to die down in his or her own time, even if your government says it is bad for us. [Fadipe, Male, Age:73, Secondary Education]

It was also reported as shown in the extract below that the TBAs have a duty to protect FGM/C being their inheritance. ~~It was also reported as shown in the extract below that the TBA has a duty to protect the practice as it was their inheritance that must be protected.~~

But you know, it is our heritage which must be promoted. It is not that it really makes more money for us. Do you understand me? It is not what the TBAs are eating from. But it is what was handed over to us as our heritage and must not be taken away just like that! [Abike, Female, Age: 70, Primary Education].

Defiance against government's efforts to eliminate FGM/C

This subtheme emerged from data related to the defiant attitudes of the TBAs towards seminars and workshops that had been previously arranged by government and other organizations in the country with the aim of highlighting the disadvantages of intergenerational FGM/C proposals towards eliminating FGM/C as described in the following extracts.

The extract below shows that despite the attendance at the seminar organized for the TBA by the government, stopping FGMC is going to be a disadvantage to the women because their sexual urge may not be easily controlled as we are seeing around us.

I attended the workshops about this, but I was and am still convinced that the disadvantages of government's effort to stopping FGM/C will be enormous for our women. See what is happening nowadays in our communities. No one will be able to control these girls. We must fight anyone including the government to protect our girls from promiscuity. [Lukman, Male, Age: 45, Tertiary Education]

The following extract upholds the submission by the TBAs that FGM/C prevents promiscuity which explains why the tradition has been successfully continued. ~~In the extract below, the respondent upheld that FGMC prevents promiscuity, and it is why the tradition has been successful, hence the continuity.~~

No, I don't agree with the government's decision. The result will be very bad. As for me, I did it for all my children both boys and girls. None of my children are promiscuous because I circumcised them and took care of them all. I buried what was cut and properly watered the soil where I buried it so that chicken could not be able to unearth it. Otherwise, if unearthed, the girl child will be promiscuous. We are very careful about these things so that no one can come back and say FGM/C doesn't work. [Amope, Female, Age: 80, Primary Education].

A respondent bluntly declared that she will never support the government in stopping FGM/C as depicted in the following extract. ~~The response below is from a participant who bluntly declared that she will never support the government in stopping FGMC.~~

Ah! I am not in support of the government stopping the girl child circumcision. That is our campaign, and I am not hiding it. At least, it will not happen in my time. [Abike, Female, Age 70, Primary Education]

Elimination of FGM/C is described as an advantage to the girl child that no one would want to support as shown in the quote below. ~~The extract from a respondent below described elimination of FGMC as a disadvantage to the girl child that no one would like to support.~~

Personally, I don't agree with government's reasons to stop girl child circumcision. I have said it earlier that, based on my experience and the level of my education, and coming from a family that is traditionally practicing FGM/C, I do not see it being stopped by anyone, even the government or anyone else, not now. I did it (FGM/C) for my girls and there is no problem associated with it, I don't think it should be stopped. It will be a great disadvantage if anyone could agree with government. [Lukman, Male, Age: 45, Tertiary Education].

Debunking all "Western" information about dangers of FGM/C to women as lies

This subtheme emerged from data related to TBAs efforts in debunking most of the information and claims about the benefits of eliminating intergenerational FGM/C as just government's efforts to brainwash people, against the practice of FGM/C as depicted in the quotes below.

The information about the girls having reproductive challenges later in life was debunked by the respondent as shown in the quote below. ~~The information about the girls having reproductive challenges later in life was debunked by the respondent as shown in the quote below.~~

The girls that I circumcised did not have any problem. Even when grown up, these girls do not develop any of the problems we were told resulted from FGM/C, which we were now told can be associated with FGM/C. The girls are now big women and are all alive and doing well in their families. They got married to nice men and have lively children. [Amope, Female, Age 80, Primary Education]

The extract below shows that the respondent disagrees with the record of death sequel to FGM/C. He said that the girls he circumcised as infants are still alive today. ~~In the bid to debunk the information about death sequel to FGMC, the respondent below said that all the girls he circumcised are still alive today.~~

Every girl that we have circumcised is still alive even today. No child can die in the hand of circumcision surgeon and all our girls were circumcised. Anyone who say we put our girls in danger is a liar. My wife is circumcised, all my girls are circumcised and alive. They must stop spreading lies about FGM/C. [Olalekan, Male, Age 86, Primary Education]

In addition, the likelihood of lack of enjoyment during sex was further refuted but rather held that circumcised women enjoy sex more as shown in the quote below. ~~The likelihood of lack of enjoyment during sex was further debunked but rather held that circumcised women enjoy sex more as demonstrated in the extracts below.~~

That debunked FGM/C leads to women not enjoying sex is one of the lies the government is peddling about girl circumcision. But they have forgotten that it's only a small part of the clitoris that is cut and not the whole clitoris. it is not true at all. [Adeyemi, Male, Age 53, Tertiary Education]

It is only a false assumption that circumcised women do not enjoy sex. When a circumcised woman has sexual intercourse with men, she will glue to him as if they tie penis and vagina together, in that way, they both enjoy each other nicely [Olalekan, Male Age: 86, Primary Education]

Furthermore, the views that FGM/C have complications was refuted as just rumors as captured by the following extracts. ~~Furthermore, the views that FGM/C spreads sexually transmitted infections has been refuted by the TBAs as just rumors as captured in the following extracts:~~

There is nothing like transmission of sexual diseases by FGMC, bleeding and all so many other things that are being said about female circumcision. It is safe and cultural to do it (circumcision). [Mojirola, Female, Age 63, Secondary Education]

We were also told that female circumcision causes sexual transmitted diseases / infections which is not true at all! Honestly, I don't know what the government want to gain from spreading such lies for stopping it. Female circumcision does not have any negative side effects as it is being peddled around. [Amope, Female, Age 80, Primary Education].

Discussion

Formatted: Font color: Auto

The study was conducted to assess TBAs' attitudes towards the elimination of intergenerational FGM/C of girls in Osun state, Nigeria in order to determine their level of readiness and preparedness towards achieving its elimination.

Our findings show a deep defensive attitude towards elimination of FGM/C among the TBAs as community leaders and custodians of the intergenerational FGM/C which is reflected in the participants views that FGM/C is their traditional heritage and its elimination can be equated to eliminating their cultural identity. This finding conforms with the conclusion of Dunn that the cultural identity of the Africans is being eliminated by current civilization [23] and Obi, Adesoye, Labiran that also reported in their study that, culture/tradition is the greatest human inheritance, since it contributes to the preservation of history and identity of any race, thus confirming the TBAs' beliefs about their responsibility to preserve intergenerational FGM/C as their traditional heritage despite all its disadvantages to women, their families, and communities [24].

Findings from this study also depicted a strong defiance against the government's prevention interventions as indicated in the defiance shown by those TBAs who had attended seminars and workshops organised by the government on the elimination of intergenerational FGM/C, thus inconsistent with the previous findings by [25] who concluded that unsuccessful elimination of intergenerational FGM/C was due the government's inability to involve the major community stakeholders such as the TBAs.

Therefore, due to the TBAs' defiance against governments' efforts to eliminate FGM/C, there is a continuous non-alignment between government agencies and the views and beliefs of those responsible for promotion of historic traditions (such as FGM/C) always ending up in disagreement, if not properly handled. According to the TBAs', their voices and

understanding of their attitudes towards abandoning their age long custom, and not only inviting them to attend seminars or workshops on elimination of FGM/C is important in determining the community's readiness for development and implementation of interventions for eliminating intergenerational FGM/C of girls.

Most of the information and claims about the benefits of eliminating FGM/C were debunked by the TBAs as shown from the analysis. They claimed that it is just government's efforts to brainwash people which is disputed by previous report which supports the government's assertion on the benefits of eliminating intergenerational FGM/C. This finding is also in contrast with the conclusion of [26] who reported that more patients with complications of FGM/C such as difficult delivery, perineal laceration, hemorrhage, scar/keloid etc. were seen by nurses/midwives than doctors in Yenegoa, Bayelsa, Nigeria [26].

The TBAs are of the opinion that the involvement of people who are not traditionally entitled to circumcision and are not knowledgeable enough are the ones causing the complications as stated by the government. The TBAs' debunking of all "Western" information about dangers of FGM/C to women as lies may be due to their lack of knowledge of the dangers and complications of FGM/C. Similar findings were reported by [26] who concluded limited knowledge about complications may be reflected as negative attitudes towards prevention or elimination of intergenerational FGM/C in their lifetime.

Our findings show that FGM/C is going to continue in Osun state, Nigeria despite government's efforts to eliminate intergenerational FGM/C. This is confirming the report by the UNFPA which estimated that because of population growth, additional 4.6 million girls are projected to be at risk of undergoing female genital mutilation by 2030 [27]. It was also reported that the outbreak of COVID-19 between 2020 and 2022 aggravated the vulnerability

of girls and women who are at risk of FGM/C which indicated that 2 million cases of FGM/C could be recorded over the next decade due to COVID-19 alone [27].

Limitation ~~Limitations~~

Using local language to collect data could have distorted the presentation of the questions but back and forth translation of the questions minimized the language bias.

Author contributions

Formatted: Font: 18 pt, Bold

The two authors have equal contribution to the work.

Formatted: Font: 12 pt, Not Bold

Formatted: Font: 18 pt, Bold

~~Using local language to collect data could have distorted the presentation of the questions but back-to-back translation of the questions minimized the language bias. The co-author who is also a female and a feminist remained neutral in the way data were collected, analyzed and interpreted.~~

Conclusion

The current defensive attitudes of the TBAs as community leaders are worrisome because they are an indication of the community leaders' level of no readiness for intervention programmes for the elimination of intergenerational FGM/C in Osun state, Nigeria. We recommend that the government, policy makers and health professionals elicit and carefully consider the views of the community leaders such as TBAs who are custodians of the FGM/C before introducing measures to eliminate FGM/C in traditional communities. This may include having non-confrontational dialogue between all stakeholders for understanding of

the dangers and benefits of FGM/C to the nation which can be shared from everyone for best solutions to be found.

Acknowledgement

Our sincere appreciation goes to the management of the Ministry of Health in Osun state Nigeria and University of KwaZulu-Natal for granting us the ethical approval to carry out the study. We also thank the Nurses and the Traditional Birth Attendants for creating time to grant the interviews.

Data Availability Statement: This is a qualitative study that involves 8 traditional birth attendants who agreed to participate in this study based on anonymity that all information provided will be strictly confidential. All transcripts were anonymized including the extracts that support the study's findings. Transcripts are available for research purposes only in line with the approved research protocol. This is in fulfilment of the participants confidentiality as indicated in the approved research protocol from the Humanities and Social Sciences Research Ethics Committee [HSSREC/00000924/2019] and Osun state Ministry of Health [OSHREC/PRS/569T/157] dated 20th April 2020 and 19th August 2019 respectively.

Funding: The corresponding author received student scholarship from the University of KwaZulu-Natal, South Africa through the College of Health Sciences student Scholarship.

Competing interests: The authors have no competing interests of any form as regards this study.

~~Williams-Breault BD. Eradicating Female Genital Mutilation/Cutting: Human Rights-Based Approaches of Legislation, Education, and Community Empowerment. Health Hum Rights. 2018 Dec;20(2):223-233. PMID: 30568416; PMCID: PMC6293358.~~

References

1. World Health Organization. Female Genital Mutilation. Fact sheet. 2020 Retrieved February 20, 2022, from http://www.who.int/mediacentre/factsheets/fs241/en/
2. Waigwa S, Doos L, Bradbury-Jones, Taylor J. "Effectiveness of health education as an intervention designed to prevent female genital mutilation/cutting (FGM/C): a systematic review." Reproductive health 2018; **15**(1): 62.
3. United Nations, 2015. 'The Millennium Development Goals Report 2015', pg. 38. http://www.un.org/millenniumgoals/2015_MDG_Report/pdf/MDG%202015
4. UNICEF. Female Genital Mutilation/Cutting: A Global Concern UNICEF, New York, 2016.
5. World Health Organization. Female genital mutilation: Key facts. 2018 [cited 1 May 2022]. https://www.who.int/news-room/fact-sheets/detail/female-genital-mutilation
6. UNFPA-UNICEF Joint Programme on the Elimination of Female Genital Mutilation (2019).
7. Nigeria Demographic and Health Survey (2013). National Population Commission. Federal Republic of Nigeria.
8. Berg RC, Denison E. Health Care for Women International A Tradition in Transition: Factors Perpetuating and Hindering the Continuance of Female Genital Mutilation / Cutting (FGM / C) Summarized in a Systematic Review A Tradition in Transition: Factors Perpetuating Genital. 2013;9332(October 2017).
9. Yaya, S. and B. Ghose (2018). "Female Genital Mutilation in Nigeria: A Persisting Challenge for Women's Rights." Social Sciences 7(12): 244.

10. Reisel, D. and S. M. Creighton (2015). "Long term health consequences of Female Genital Mutilation (FGM)." Maturitas 80(1): 48-51.

11. Violence against Persons (Prohibition) Act,2015, Laws of the Federation of Nigeria

12. National Population Commission of Nigeria. Nigeria Population and Housing Census 2006. Abuja, Nigeria: National Population Commission of Nigeria.

13. Omole, F.K., Lukman, Y. and Baki, A.I. (2014), "Analysis of market typology and functions in the development of Osun state, Nigeria", International Journal of Development and Sustainability, Vol. 3 No. 1, pp. 55-69

14. Barret H. Alhassan Y. The REPLACE Community Readiness to End FGM Assessment. Practical Handbook (based on REPLACE Approach www.replacefgm2.eu) 2016, Coventry university, UK.

15. McCoy H.V, Malow R., Edwards R.W., Thurland A., Rosenberg R. A Strategy for Improving Community Effectiveness of HIV/AIDS Intervention Design: The Community Readiness Model in the Caribbean, Substance Use & Misuse, 2007; 42:10, 1579-1592, DOI: 10.1080/10826080701212535

16. Borrayo, E. A. (2007). "Using a community readiness model to help overcome breast health disparities among US Latinas." Substance use & misuse **42**(4): 603-619.

17. Duma, S. Women's journey of recovery from sexual assault trauma: a grounded theory.2006.

18. Van Teijlingen, E R, Hundley V. "The importance of pilot studies." (2001).

19. Braun V, Clarke V. Thematic analysis. in H. Cooper, P. M. Camic, D. L. Long, A. T. Panter, D. Rindskopf, & K. J. Sher (Eds.), *APA handbook of research methods in psychology, 2012;Vol. 2. Research designs: Quantitative, qualitative, neuropsychological, and biological* (pp. 57–71). American Psychological Association. https://doi.org/10.1037/13620-004

20. Louis Cohen L, Manion L, Morrison K. Action research. Research methods in education, Routledge 2013: 368-385.
21. Polit, D. and C. Beck. "Essentials of nursing research." Ethics 2012; **23**(2).
22. Fawcett J, Garity J. "Chapter 1: Research and evidence-based nursing practice." Evaluating research for evidence-based nursing: (2009); 3-20.
23. Dunn F. Is It Possible to End Female Circumcision in Africa? Clin J Obstet Gynecol. 2021;1: 007-013. https://doi.org/10.29328/journal.cjog.1001002
24. Obi A.I., Adesoye O., Labiran A. Female Genital Mutilation: Attitude and Practices among Women in Okada Community, Edo State JMBR: A Peer-review Journal of Biomedical Sciences December 2015, Vol. 14 No. 2 pp 138-150
25. Andarge, Masresha Yazew. *The difficulties of ending Female Genital Mutilation (FGM): Case of Afar pastoralist communities in Ethiopia*. *Social Policy for Development (SPD) (2014, December 12).* Retrieved from http://hdl.handle.net/2105/17487
26. Ibrahim I.A, Oyeyemi A.S, Ekine A.A. Knowledge, Attitude and Practice of Female Genital Mutilation among Doctors and Nurses in Bayelsa state, Niger-Delta of Nigeria. International Journal of Medicine and Biomedical Research Volume 2 Issue 1 January – April 2013
27. UNFPA. 2020. ‘Impact of the COVID-19 Pandemic on Family Planning and Ending Gender-Based Violence, Female Genital Mutilation and Child Marriage.’ Interim Technical Note.

Map of Osun State, Nigeria showing the various towns Map of Osun state, Nigeria showing the various towns.

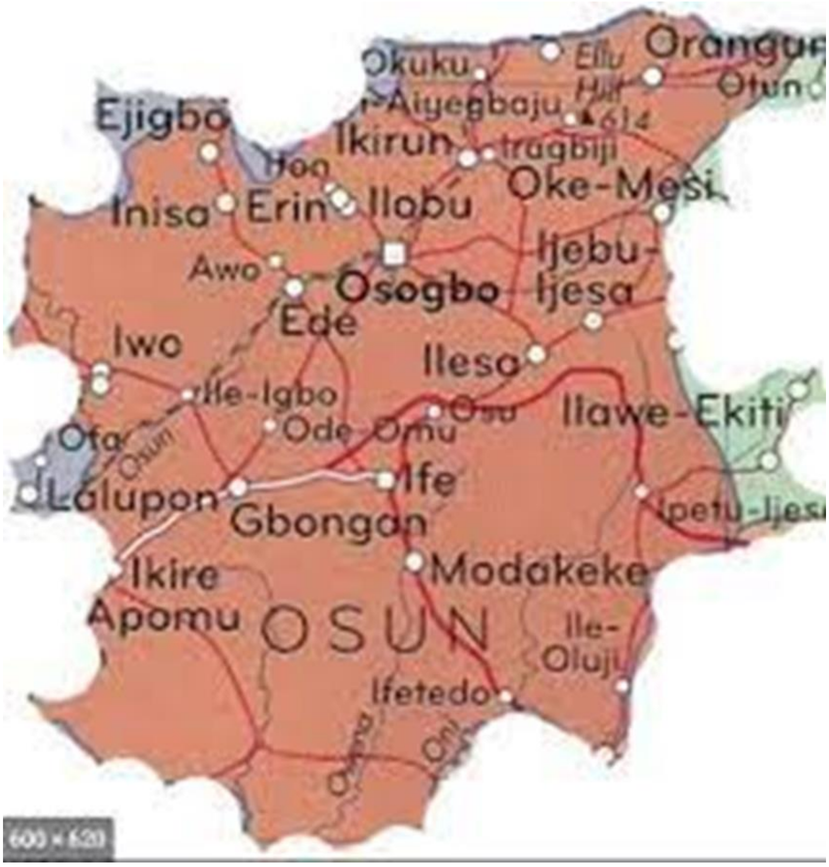

Adapted from https://dawncommission.org

Revised Manuscript with Track Changes

Full Title:

‘It Is What Was Handed Over to Us as Our Heritage and Must Not Be Taken Away Just Like That’: Traditional Birth Attendants’ Attitudes Towards the Elimination of Intergenerational Female Genital Mutilation/Cutting in Osun State, Nigeria.

Short Title:

Eliminating Intergenerational Female Genital Mutilation/Cutting in Osun State, Nigeria.

Authors:

ROSEMARY OMOLARA FAFOWORA[1¶*ID] orcid.org/0000-0003-2968-3784

SINEGUGU EVIDENCE DUMA[2¶ID] orcid.org/0000-0002-2489-8770

[1]Department of Nursing, School of Nursing and Public Health, College of Health Sciences, Howard Campus, University of KwaZulu-Natal, Durban, South Africa.

[2]Department of Nursing, School of Nursing and Public Health, College of Health Sciences, Howard Campus, University of KwaZulu-Natal, Durban, South Africa.

*Corresponding author

E-mail:rosemary.olagunju@gmail.com (RO)

~~¶The authors equally contributed to this work.~~

Abstract

Background

Female genital mutilation/cutting is a harmful practice that violates the sexual and reproductive health rights of women and girls. The practice is often perpetrated directly or indirectly from one generation to another as a way of preserving culture, thus making it difficult to tackle using ordinary prevention interventions. The purpose of the study was to assess the attitude of the traditional birth attendants as community leaders towards the elimination of intergenerational female genital mutilation/cutting and to determine their level of readiness and preparedness towards achieving it in Osun State, Nigeria.

Methodology/Results

A qualitative research design, using the adapted REPLACE community readiness tool to end female genital mutilation/cutting interview guide, was used to individually interview eight traditional birth attendants who were identified through purposive sampling method as community leaders and key informants. Thematic Analysis was used to analyze the data which yielded female genital mutilation/cutting as traditional heritage, defiance against government efforts and debunking all "western" information about dangers of female genital mutilation to women as lies as findings. These findings are worrisomeThe current defensive attitudes of the TBAs as community leaders and custodians of the FGMC tradition are an indication of low level of no readiness for any intervention to prevent or eliminate FGMC in Osun state, Nigeria.

Conclusion

Serious engagement and dialogue between policy makers and health professionals on FGMC and its effects on women is highly recommended for effective FGMC elimination strategies to be co-developed with community leaders. Such engagements should adopt a non-confrontational, respectful and honest approach so as to not alienate the TBAs.

Commented [SED1]: You can choose to use these words or maintain yours. I used these because that is how I concluded the article.

because they are an indication of the community leaders' level of no readiness for interventions for elimination of intergenerational female genital mutilation/cutting in Osun state, Nigeria.

Conclusion

We recommend that the government, policy makers and health professionals elicit and carefully consider the views of the community leaders such as traditional birth attendants who are custodians of the female genital mutilation/cutting before introducing measures to eliminate it in the traditional communities. This may include having non-confrontational dialogue between all stakeholders, for understanding of the dangers and benefits of female genital mutilation/cutting to the nation which can be shared from everyone for best solutions to be found.

Key Words: Replace Community Readiness Tool, Intergenerational Female Genital Mutilation/Cutting, Traditional Birth Attendant

Introduction

Female genital mutilation/cutting (FGM/C) is a public health problem globally. It comprises all procedures that involve partial or total removal of the external female genitalia, or other injury to the female genital organs for non-medical reasons. It is also referred to as multiple procedures on the female genital organs for non-medical reasons [1]. FGM/C has been described as a harmful practice that violates the sexual and reproductive health rights of women and girls [2,3]. It is estimated that more than 200 million girls and women alive today have undergone FGM/C across 30 countries including the Middle East, Asia, and Africa [4]. Of this, about 44 million are girls under the age of 15 years [5]. It is further projected that more than 3 million girls and women are currently at risk of FGM/C worldwide [4]. The

current estimates of 3.9 million girls mutilated annually will also rise to 4.6 million by 2030 because there is an increase in the population growth in the communities that still practice FGM/C [6]. It is reported that 20 million girls and women are estimated to have undergone FGM/C in Nigeria, with Osun state, where the study was conducted, having reached the highest prevalence of 76.6% of FGM/C [7].

In Nigeria, FGM/C is a deeply entrenched custom that is commonly performed by the traditional birth attendants (TBAs) who claim it as a heritage which confers honor on a woman and her family, a rite of passage to womanhood, preservation of chastity, enhancement of fertility, and prevention of death of the child during delivery among its benefits [8].

Despite its cultural importance, FGM/C has received considerable criticism worldwide because of its potential risk for various forms of health complications [9]. The short term and physical health complication of FGM/C includes but are not limited to pain, risk of hemorrhage, shock, tetanus/sepsis, inability to urinate, damage to adjacent organs such as the rectum and urinary tract, dislocation and fracture of the pelvic bones and the head of the femur due to struggle while being restrained, open sore in the genital region, risk of HIV/AIDS or Hepatitis B. While the long term and reproductive health consequences of FGM/C include difficulty in passing menstrual blood, vaginal fistula, painful sexual intercourse, and difficulty in passing urine which can lead to recurrent urinary tract infection, clitoral cyst, infertility, an increased risk of childbirth complication and newborn death, need for future surgeries to allow for sexual intercourse and childbirth, psychological trauma, and risk of death from hemorrhage or infection [10].

Furthermore, sexuality is an equal right that both males and females must benefit from. While men are being circumcised for health and religious reasons, women and girls are cut deliberately to remove their sexual feelings and subjugate them to the dictates of their men counterpart, and it is done without any consent from them.

In 2005, the Violence Against Persons (Prohibition) Act 2015 was passed to eliminate the FGM/C practice in Nigeria. Since then, several legal, community based, and health-based strategies had been instituted to eliminate the FGM/C practice [11]. These strategies were met with strong opposition from the TBAs, who remain the self-proclaimed custodians of the traditional responsibility of FGM/C from time immemorial, especially in Osun State, Nigeria. The TBAs believe in the supremacy of FGM/C as the traditional heritage, which is concerned about the wellbeing of the people, that it predates modern medicine and therefore should not be stopped. This cultural belief has facilitated the intergenerational preservation of FGM/C and explains their attitude towards the elimination of its practice. The intergenerational preservation of FGM/C is reflected on most women's belief that the elimination of FGM/C is tantamount to defaulting from their religion or traditions which could breed calamity for them and their offspring. These beliefs have been passed down from one generation to the other and contribute to the high prevalence of FGM/C in Osun State, Nigeria, where it has become major public health issue that requires public health approach-based prevention interventions. Because of their influence on the intergenerational preservation of FGM/C, we identified the TBAs as community leaders who are key towards the development and implementation of public health prevention intervention for FGM/C in Osun State, Nigeria. The purpose of the study was to assess the attitude of the TBAs as community leaders towards the elimination of intergenerational FGM/C of girls in Osun State, Nigeria, in order to determine their level of readiness and preparedness towards achieving its elimination.

Materials and Methods

Study setting

Osun State is an inland state in South-West Nigeria with a population of 3,423,535 [12]. The present Osun State was created in 1991 from the old Oyo State. The state shares boundary with Kwara State in the North, Oyo in the West, Ogun and Ondo in the South and Ekiti State on East. The state capital is Osogbo, which is a local government area on its own. In all, a total of thirty local government council areas and Ife east area council constitute the state. Among the major towns in the state are Ilesha, Ife, Ede, Ikirun, Ikire, Ejigbo and Osogbo [13]. Before this time, the state was part of the old Oyo State. Osun State experiences a tropical climate while the local vegetation is the lowland rainforest type.

Indigenes of Osun State belong to the Yoruba race and comprise the Oyo, Ife, Ijesa, Igbomina and Osun. Many non-indigenes and foreigners also reside in the state and live together in harmony. Yoruba and English are the languages used for official and business transactions. The people have a rich cultural heritage that is demonstrated in all spheres of their life including the performance of FGM/C. Their culture finds expression in their arts, literature, music, and other social activities.

This study was conducted among the Yoruba TBAs residing across the three senatorial districts in Osun State, Nigeria. The Yoruba people have a rich cultural heritage that is demonstrated in all spheres of their life including the performance of FGM/C in the Osun State, Nigeria.

Research design

A qualitative research design, using the adapted REPLACE community readiness to end FGM/C interview guide [14] was used to assess the attitudes of the TBAs as community leaders towards elimination of intergenerational FGM/C to determine their level of readiness and preparedness towards achieving its elimination in Osun State, Nigeria. This was the most suitable approach because it focuses on social and cultural contexts to understand how a problem is perceived and whether taking some type of action is seen as needed by community leaders to change the problem [15, 16]. The adapted REPLACE community readiness to end FGM/C interview guide [14] was translated to the local (Yoruba) language by a bilingual expert in Yoruba and English who is also a qualitative researcher for comparison of the language translation to avoid the loss of meaning in the translated interview guides. The same person was used for translating transcripts to English.

Sampling and recruitment of participants

Purposive sampling method was used select eight TBAs whom we were regarded as community leaders and key informants with rich information regarding intergenerational FGM/C in Osun State, Nigeria. The inclusion criteria for recruitment and participation in the study included only TBAs who were formally recognized as leaders in performing FGM/C, who had been previously involved in any of the Government's organized seminars or workshops on FGM/C elimination and or prevention intervention in Osun State, irrespective of the duration of the practice. The selection and recruitment of the participants commenced once the ethical approval from the Humanities and Social Sciences Research Ethics Committee [HSSREC/00000924/2019] and Osun state Ministry of Health [OSHREC/PRS/569T/157] was obtained. Meetings were held with the nurses in the maternity units across the three senatorial districts as gatekeepers to inform them about the purpose of the study and to seek their permission to meet the TBAs working in their respective Units. A

meeting with TBAs was held where the purpose of the study, the research eligibility criteria and request for voluntary participation from those who meet the inclusion criteria were discussed. Those who showed interest were recruited and were given the information sheet and the informed consent form to complete and sign. Arrangements for the interview session were then made by allowing the potential participants to choose suitable date and time within the first 7 days after the participants have given their consent. Before the commencement of the interview, the purpose of the study, and permission to audio record the interview sessions were explained again to the participants in the local language (Yoruba). A total sample size of eight traditional birth attendants as community leaders and key informants were recruited in the study. This small sample size is in line with the REPLACE community readiness tool, which suggests 6-12 key informants as sufficient to address issues in the community [14].

Pilot study

The first two TBAs out of the sample size were used for pilot study to ensure that the interview guide questions were easy and yielded the necessary data. The data generated from the pilot study were included in the main study data analysis. Contamination of data was not considered to be a concern because in qualitative studies data collection is progressive and the data from pilot studies can be used for the main study [17,18].

Data collection

Data collection and data analysis were conducted concurrently between October 2020 and March 2021. All COVID-19 precaution protocols like using the face mask, maintaining social distancing and hand sanitizing were observed during the interviews. In-depth interviews were conducted with the individual TBAs in their homes by the first author in Yoruba, the local language, using the interview guide adapted from the REPLACE Community Readiness to

End FGM/C Assessment tool [14]. The interviews were audio recorded and transcribed within 24 hours of recording when information was still fresh in the researcher's memory for data analysis purposes. Each interview lasted between 45-60 minutes to complete thus allowing each TBA to adequately share their views as attitudes towards elimination of intergenerational FGM/C in Osun state. Nigeria. The transcripts were stored in word document formats on a computer with encrypted password and was backed up to an external hard drive to prevent data loss in line with the University's principles for data storage.

Data analysis

The six steps, namely: familiarization, coding, searching for themes, reviewing themes, defining, and naming themes, and writing up as described by [19] were used to analyze the transcribed data.

The first author went through the transcripts reading and re-reading them to familiarise herself and make sense of the data. Notes were taken and preliminary ideas for codes were marked. In order to start organizing the data into meaningful groups, codes were assigned to briefly describe something interesting that was said by the participants. The first author grouped the newly generated codes with same meaning together to form themes. The second author, who also doubled as the inter coder at this stage, also did the same thing. The generated themes by the first and second authors were scrutinized by both of them to ensure that they were useful and accurate representation of the data by discarding some themes and creating new ones from the transcribed data. The first author then formulated the meaning of each theme so that the data can be understood. At this stage, appropriate naming of themes was made to succinctly interpret the data collected.

Ethical Permission

All ethical principles were adhered to including, consent form for voluntary participation, ethical approval from the Humanities and Social Sciences Research Ethics Committee [HSSREC/00000924/2019] and Osun state Ministry of Health [OSHREC/PRS/569T/157] were obtained.

The names assigned to each of the participants are not their actual names as we are aware of the ethical implication. However, using pseudonyms instead of participants' actual names ensured confidentiality and anonymity.

Measure to ensure trustworthiness

Measures to ensure trustworthiness and rigor of the findings were applied through the criteria of credibility, confirmability, dependability, and transferability [20].

To ensure credibility, comprehensive field notes, noting gesture and other non-verbal ques observed during the interviews were recorded and analyzed. Audit trail of all research processes were recorded. Analysis of the data gathered reflected a neutral interpretation of the data rather than the researcher's view as recommended by [21,22]. This was achieved by conducting a member checking on data whereby the analyzed data was summarized and provided to the participants for them to validate and determine the accuracy of the data as their views. For dependability and transferability, an audit trail was kept and the comprehensive and rich details of the study setting, and the socio-demographic characteristics of the participants are presented. To further establish trust and confidence in the findings, the authors compared their individually generated themes from the raw data to ensure that they were useful and accurate representation of the data by discarding some themes and creating new ones from the transcribed data. The senior author and research supervisor reviewed the

themes against raw data and helped in refining these to reflect participants' views to enhance confirmability. Common issues recur and main themes were identified to summarize all the views that have been captured in the data. One theme and three subthemes emerged that revealed the defensive attitude of the TBAs towards the elimination of intergenerational FGM/C of girls in Osun state, Nigeria.

Results

Eight (8) TBAs, comprising of three females and five males participated in the study on TBA's attitude towards the elimination of intergenerational FGM/C of girls in Osun State, Nigeria.

Table 1: Participants' Demographic Characteristics

Participant	Age	Gender	Religion	Educational level
Lukman	45	Male	Islam	Tertiary education
Abike	70	Female	Islam	Primary education
Adeyemi	53	Male	Islam	Tertiary education

Olalekan	86	Male	Islam	Primary education
Mojirola	63	Female	Christian	Secondary education
Amope	80	Female	Islam	Primary education
Fadipe	73	Male	Islam	Secondary education
Adebayo	75	Male	Islam	Primary education

These demographics reflects the TBAs in Osun State where the person who becomes a TBA is culturally determined through the lineage of the traditional circumcisers. It is a taboo for a woman who is married to perform FGM/C as TBA, but she can still retain her knives for performing FGM/C on her own children only (personal conversation with Mr Adeyemi, a male participant to the study, December 2020). This explains why most participants were male TBAs in the study as reflected in Table 1. Their ages ranged between 45 and 86 years. Seven of them practiced Islam and only one was Christian as a religion.

Defensive attitude towards elimination of intergenerational FGM/C

Analyzed data yielded "Defensive attitude towards elimination of FGM/C" as the main theme in the TBAs' attitude towards the elimination of intergenerational FGM/C of girls in Osun State, Nigeria. Three subthemes, including (i) FGM/C as a traditional heritage, (ii) Defiance

against government's efforts to eliminate intergenerational FGM/C and Debunking all "western" information about dangers of FGM/C as lies emerged under the main theme.

FGM/C as traditional heritage

The FGM/C as a traditional heritage sub theme emerged from data related TBAs' defensive attitudes towards elimination of intergenerational FGM/C as traditional and cultural beliefs that the practice is the identity and the only link between the current and past among generations as described in the following extracts.

The response from the respondent below upheld the fact that FGMC is their traditional heritage that cannot be stopped in their lifetime.

I am talking about our traditional heritage here. It can't be stopped; it can't stop now. The law, civilization coupled with ignorance is stopping people, but it cannot be stopped. [Lukman, Male, Age: 45, Tertiary Education]

Another respondent also mentioned that FGM/C is a traditional heritage that was handed down by their forefathers.

I think all they are saying about stopping of our tradition is done out of civilization and hatred, hatred of us and our traditional heritage. What has been established by our forefathers cannot perish. It is just like asking us to abandon our language because of civilization. There is no where we meet that we can do without speaking our language. Girl child circumcision cannot perish. [Abike, Female, Age:70, Primary Education]

Further, data under this subtheme showed that TBAsconsiderate as their primary responsibility to prevent intergenerational practice of FGM/C from extinction as depicted in the following extract.

Girl circumcision has been in existence from time immemorial. Therefore, it can be described as our cultural identity that was transmitted from generation to generation that no one is ready to allow it to die down in his or her own time, even if your government says it is bad for us. [Fadipe, Male, Age:73, Secondary Education]

It was also reported as shown in the extract below that the TBAs have a duty to protect FGM/C being their inheritance.

But you know, it is our heritage which must be promoted. It is not that it really makes more money for us. Do you understand me? It is not what the TBAs are eating from. But it is what was handed over to us as our heritage and must not be taken away just like that! [Abike, Female, Age: 70, Primary Education].

Defiance against government's efforts to eliminate FGM/C

This subtheme emerged from data related to the defiant attitudes of the TBAs towards seminars and workshops that had been previously arranged by government and other organizations in the country with the aim of highlighting the disadvantages of intergenerational FGM/C proposals towards eliminating FGM/C as described in the following extracts.

The extract below shows that despite the attendance at the seminar organized for the TBA by the government, stopping FGMC is going to be a disadvantage to the women because their sexual urge may not be easily controlled as we are seeing around us.

I attended the workshops about this, but I was and am still convinced that the disadvantages of government's effort to stopping FGM/C will be enormous for our women. See what is happening nowadays in our communities. No one will be able to control these girls. We must

fight anyone including the government to protect our girls from promiscuity. [Lukman, Male, Age: 45, Tertiary Education]

The following extract upholds the submission by the TBAs that FGM/C prevents promiscuity which explains why the tradition has been successfully continued.

No, I don't agree with the government's decision. The result will be very bad. As for me, I did it for all my children both boys and girls. None of my children are promiscuous because I circumcised them and took care of them all. I buried what was cut and properly watered the soil where I buried it so that chicken could not be able to unearth it. Otherwise, if unearthed, the girl child will be promiscuous. We are very careful about these things so that no one can come back and say FGM/C doesn't work. [Amope, Female, Age: 80, Primary Education].

A respondent bluntly declared that she will never support the government in stopping FGM/C as depicted in the following extract.

Ah! I am not in support of the government stopping the girl child circumcision. That is our campaign, and I am not hiding it. At least, it will not happen in my time. [Abike, Female, Age 70, Primary Education]

Elimination of FGM/C is described as an advantage to the girl child that no one would want to support as shown in the quote below.

Personally, I don't agree with government's reasons to stop girl child circumcision. I have said it earlier that, based on my experience and the level of my education, and coming from a family that is traditionally practicing FGM/C, I do not see it being stopped by anyone, even the government or anyone else, not now. I did it (FGM/C) for my girls and there is no problem associated with it, I don't think it should be stopped. It will be a great disadvantage if anyone could agree with government. [Lukman, Male, Age: 45, Tertiary Education].

Debunking all "Western" information about dangers of FGM/C to women as lies

This subtheme emerged from data ~~relatedto~~related to TBAs efforts in debunking most of the information and claims about the benefits of eliminating intergenerational FGM/C as just government's efforts to brainwash people, against the practice of FGM/C as depicted in the quotes below.

The information about the girls having reproductive challenges later in life was debunked by the respondent as shown in the quote below.

The girls that I circumcised did not have any problem. Even when grown up, these girls do not develop any of the problems we were told resulted from FGM/C, which we were now told can be associated with FGM/C. The girls are now big women and are all alive and doing well in their families. They got married to nice men and have lively children. [Amope, Female, Age 80, Primary Education]

The extract below shows that the respondent disagrees with the record of death sequel to FGM/C. He said that the girls he circumcised as infants are still alive today.

Every girl that we have circumcised is still alive even today. No child can die in the hand of circumcision surgeon and all our girls were circumcised. Anyone who say we put our girls in danger is a liar. My wife is circumcised, all my girls are circumcised and alive. They must stop spreading lies about FGM/C. [Olalekan, Male, Age 86, Primary Education]

In addition, the likelihood of lack of enjoyment during sex was further refuted but rather held that circumcised women enjoy sex more as shown in the quote below.

That debunked FGM/C leads to women not enjoying sex is one of the lies the government is peddling about girl circumcision. But they have forgotten that it's only a small part of the

clitoris that is cut and not the whole clitoris. it is not true at all. [Adeyemi, Male, Age 53, Tertiary Education]

It is only a false assumption that circumcised women do not enjoy sex. When a circumcised woman has sexual intercourse with men, she will glue to him as if they tie penis and vagina together, in that way, they both enjoy each other nicely [Olalekan, Male Age: 86, Primary Education]

Furthermore, the views that FGM/C have complications was refuted as just rumors as captured by the following extracts.

There is nothing like transmission of sexual diseases by FGMC, bleeding and all so many other things that are being said about female circumcision. It is safe and cultural to do it (circumcision). [Mojirola, Female, Age 63, Secondary Education]

We were also told that female circumcision causes sexual transmitted diseases / infections which is not true at all! Honestly, I don't know what the government want to gain from spreading such lies for stopping it. Female circumcision does not have any negative side effects as it is being peddled around. [Amope, Female, Age 80, Primary Education].

Discussion

The study was conducted to assess TBAs' attitudes towards the elimination of intergenerational FGM/C of girls in Osun state, Nigeria in order to determine their level of readiness and preparedness towards achieving its elimination. Attitudes towards intergenerational FGM/C can vary among TBAs in Osun state, Nigeria as they represent a diverse group with different beliefs and perspectives. It is important to note that TBAs attitudes [may also] reflect a broader societal attitudes and cultural norms prevalent that is prevalent in the region.

Commented [SED2]: I would not add this

In recent years, there have been significant efforts to promote the elimination of FGM/C in Nigeria. These efforts include education and awareness campaigns, legal measures, and community engagement programs. However, despite these efforts, report shows that the interviewed TBAs are still holding on to the traditional beliefs [and still] which support the practice.

Our findings show a deep defensive attitude towards elimination of FGM/C among the TBAs as community leaders and custodians of the intergenerational FGM/C which is reflected in the participants views that FGM/C is their traditional heritage and equating its elimination can be equated to eliminating their cultural identity. This finding conforms with the conclusion of Dunn [2018] that the cultural identity of the Africans is being eliminated by current civilization in their research article that shed light on how possible it is to end female circumcision in Africa. [23] . and SObi, Adesoye, Labiranakeah et al (2019) that also reportidentified tradition/culture as one of the contributory factors to the persistent practice of FGM/C in their ed in their studyresearch article thatwhich discussed the narratives from women and men in northern Ghana about the persistent in FGM/C despite its illegality, culture/tradition is the greatest human inheritance, since it contributes to the preservation of history and identity of any race, thus confirming the TBAs' beliefs about their responsibility to preserve intergenerational FGM/C as their traditional heritage despite all its disadvantages to women, their families, and communities [24].

Our findings revealed that The TBAs were of the beliefbelieved that FGM/C is a cultural tradition that must not be taken away in a similar the way that others traditions were eroded by . Besides, many of them also condemn the insincerity on the side of the colonial masters that who gradually stripped their subjects off their cultural heritage. -Despite its cultural importance, however, FGM/C has received considerable criticism worldwide because of its potential risk for various forms of health complications. This was supported by Yaya and Ghose, 2018 in their study with the aims of measuring the trends of FGM/C in Nigeria and its persisting challenge for women's right where various complications of FGM/C were identified, among which are clitoral cyst formation, hemorrhage, fistula, obstetric complications, urinary infection and retention, vagina tears and psychological trauma [9].

Commented [SED3]: Our data revealed that the TPAs are still holding.....

Commented [SED4]: ? Erasing their cultural identity

Commented [SED5]: Similar conclusion was made by Dunn (2018), whose research on ......in Africa reported that the cultural identity of the Africans was eliminated by current civilization ....

Commented [SED6]: Sakeah et al (2019) further identified tradition/culture as one of the contributory factors to the persistent practice of FGM/C in Ghana despite it being considered illegal in that country.

Commented [SED7]: This sounds like someone's work. I do not have time to check it on Turnitin but trust that you will have the reference at least!

Commented [SED8]: This does not seem to fit here for me. Perhaps you can use it in the introduction/ background section.

Findings from this study also depicted a strong defiance against the government's prevention interventions as indicated in the defiance shown by those TBAs who had attended seminars and workshops organised by the government on the elimination of intergenerational FGM/C, thus inconsistent with the previous findings by Andarge Masresha Yazew (2014) where factors contributing to the difficulties of ending FGM/C in Afar pastoralist communities in Ethiopia were [identified [255]. who It was concludednoted that unsuccessful elimination of intergenerational FGM/C was due the government's inability to involve the major community stakeholders such as the TBAs.

Therefore, due to If not properly handled, the TBAs' defiance against governments' efforts to eliminate FGM/C, can result in there is a continuous non-alignment and disagreements between government agencies and the views and beliefs of those responsible for promotion of historic traditions (such as FGM/C) as was reported by Dunn [23]. always ending up in disagreement, if not properly handled. This is similar to what Dunn [23] mentioned in an article that discussed the possibility to end FGM/C. According to the TBAs', their voices and understanding of their attitudes towards abandoning their age long custom, and not only inviting them to attend seminars or workshops on government should see them as stakeholders in the elimination of FGM/C, which is important in determining the community's readiness for development and implementation of interventions for eliminating intergenerational FGM/C of girls. This is in line with the research paper presented by Masresha Yazew Andarge (2014) where the difficulties of ending FGM/C in Afar pastoralist communities in Ethiopia were identified (26). It was suggested in the that to eradicate FGM/C, Recognising it is important to understand how deep the cultural influence is in continuing the tradition in the name of sustaining culture. the profound extent of cultural influence that underlies the perpetuation of traditions as it plays a pivotal role in preserving and safeguarding cultural heritage cannot be underestimated The TBAs interviewed insisted

Commented [SED9]: The findings on TBa's strong defiance against the government's prevention interventions such as seminars and workshops organised by the government on the elimination of intergenerational FGM/C is contrary to the findings by …..(2014) who reported that that unsuccessful elimination of intergenerational FGM/C in Ethiopia was due the government's inability to involve the major community stakeholders such as the TBAs.
.

Formatted: Underline, Font color: Blue

Formatted: Underline, Font color: Blue

Formatted: Underline, Font color: Blue

Formatted: Underline, Font color: Blue

Formatted: Underline, Font color: Blue

Formatted: Underline, Font color: Blue

that for by the government to be in the , successful in the campaign against FGM/C, they must be carri.

[Most of the information and claims about the benefits of eliminating FGM/C. were debunked by the TBAs as shown from the analysis]. According to the They TBAs all the government's efforts to eliminate FGMC was perceived claimed that it is justas the government's efforts to brainwash people, thus debunking all information and claims about the benefits of eliminating FGM/C as reported previously by XYZ who concluded that SEE GREEN which is disputed by previous report which supports the government's assertion on the benefits of eliminating intergenerational FGM/CIbrahim I.A et al. 2013. and Yenegoa, Bayelsa, Nigeria [26] who both reported that more patients with complications of FGM/C such as difficult delivery, perineal laceration, hemorrhage, scar/keloid etc. were seen by nurses/midwives than doctors This finding is also in contrast with the conclusion of Ibrahim I.A et al. 2013 in their study in Yenegoa, Bayelsa, Nigeria [26] where it was [26] who reported that more patients with complications of FGM/C such as difficult delivery, perineal laceration, hemorrhage, scar/keloid etc. were seen by nurses/midwives than doctors, thus indicating that emiminating FGMC will be beneficial in preventing these complications. However, limited or The TBAs lack knowledge about complications of FMGC may be reflected as negative attitudes towards prevention or elimination of intergenerational FGM/C. in their lifetime which may be the case of the TBAs claiming that the complications are all lies and an effort the white people to rip them of their age long tradition. On the other hand, they holding the viewpoint that the complications from FGM/C are arising due to the participation of informal "fake" TBAs is a dangerous viewpoint that needs to be corrected if FMGC is to be eliminated. individuals who are not typically burdened with such responsibilities. in Yenegoa, Bayelsa, Nigeria [26].

Formatted: Underline, Font color: Blue

Formatted: Underline, Font color: Blue

Formatted: Underline, Font color: Blue

Formatted: Highlight

Commented [SED10]: Delete this

Formatted: Highlight

Formatted: Highlight

Formatted: Highlight

Commented [SED11]: I do not know if you need to say this or just that they concluded

Formatted: Highlight

The TBAs are of the opinion that the involvement of people who are not traditionally entitled to circumcision and are not knowledgeable enough are the ones causing the complications as stated by the government. The TBAs' debunking of all "Western" information about dangers of FGM/C to women as lies may be due to their lack of knowledge of the dangers and complications of FGM/C. Similar findings were reported by [26] who concluded limited knowledge about complications may be reflected as negative attitudes towards prevention or elimination of intergenerational FGM/C in their lifetime.

Our findings show that FGM/C is going to continue silently in Osun state, Nigeria despite government's efforts to eliminate intergenerational FGM/C. Apart from factors raised above, COVID 19 pandemic has had various impacts on global health and welfare, including the continuation of harmful practices such as FGM/C. The pandemic has presented challenges in combating FGM/C and protecting girls and women at risk.

This is confirming the report by the UNFPA (2020) which estimated that because of population growth, additional 4.6 million girls are projected to be at risk of undergoing female genital mutilation by 2030 [27]. In addition to that, it was also projected that 2 million cases of FGM/C could be recorded over the next decade due to COVID-19 alone [27] . . It was also reported that the outbreak of COVID-19 between 2020 and 2022 aggravated the vulnerability of girls and women who are at risk of FGM/C which indicated that 2 million cases of FGM/C could be recorded over the next decade due to COVID-19 alone [27].

Strength and Limitation

Using the adapted REPLACE community readiness to end FGM/C interview guide [14] was used to assess the attitudes of the TBAs as community leaders towards elimination of

Commented [SED12]: I am sure you are trying to respond to a comment by reviewers but you did not give me those – so I do not know exactly how to assist you, but I would write this paragraph differently eg.

The social isolation of women and young girls within their families during Covid-19 pandemic also put more girls at risk for FGM/C and protecting girls and women at risk. Both formal and informal and formal TBAs could continue with the practice without anyone stopping them. This therefore confirmed the report by the UNFPA (2020) which estimated that additional 4.6 million girls could be at risk of undergoing female genital mutilation by 2030. YOU CAN SEE HOW BEST TO CHANGE THIS.

Commented [SED13]: This study used a qualitative approach thus encouraging the TBAs as custodians of FGMC traditions to voice their views on FGMC freely with the researchers. Such openness helped in identifying deeply held beliefs that need to be addressed for effective elimination of FGMC at community level. However, the fact that the gender and professions of the researcher (a woman and a nurse) could be perceived as a limitation because most TBAs were men. Some of their responses could be seen as men exerting their dominance on women. YOU CAN SAY IN A NICER WAY. LIMIT THIS TO ONE STRNGTH AND ONE LIMITATion.

intergenerational FGM/C to determine their level of readiness and preparedness towards achieving its elimination in Osun State, Nigeria. This approach was found suitable because it focuses on social and cultural contexts to understand how a problem is perceived and whether taking some type of action is seen as needed by community leaders to change the problem [15, 16].

Several limitations were encountered in this study. Using local language to collect data could have distorted the presentation of the questions but back and forth translation of the questions minimized the language bias.

Formatted: Font: Not Bold

As a female interviewer, there is likelihood of introducing bias during the interview on the FGM/C. However, conscious efforts were made to ensure that the questions were asked in a way that does not influence the response of the participants, the analysis and the interpretation of data collected.

Another limitation is the reliability of the responses provided by the TBAs because they may not provide an honest response about their beliefs especially now that there have been campaigns against FGM, and there is legal repercussions to having practiced FGM

Formatted: Font: (Default) Times New Roman, 12 pt

Formatted: Font: 12 pt

Using local language to collect data could have distorted the presentation of the questions but back and forth translation of the questions minimized the language bias.

Conclusion

The current defensive attitudes of the TBAs as community leaders are worrisome because they are an indication of the community leaders' level of no readiness for intervention programmes for the elimination of intergenerational FGM/C in Osun state, Nigeria.We recommend that the government, policy makers and health professionals elicit and carefully consider the views of the community leaders such as TBAs who are custodians of the FGM/C before introducing measures to eliminate FGM/C in traditional communities. This may include having non-confrontational dialogue between all stakeholders for understanding of the dangers and benefits of FGM/C to the nation which can be shared from everyone for best solutions to be found.

Commented [SED14]: I would say;

The current defensive attitudes of the TBAs as community leaders and custodians of the FGMC tradition are an indication of low level of no readiness for any intervention to prevent or eliminate FGMC in ……Serious engagement and dialogue between policy makers and health professionals on FGMC and its effects on women is highly recommended for effective FGMC elimination strategies to be co-developed with community leaders. Such engagements should adopt a non-confrontational, respectful and honest approach so as to not alienate the TBAs.

Acknowledgement

Our sincere appreciation goes to the management of the Ministry of Health in Osun state Nigeria and University of KwaZulu-Natal for granting us the ethical approval to carry out the study. We also thank the Nurses and the Traditional Birth Attendants for creating time to grant the interviews.

Data Availability Statement: This is a qualitative study that involves 8 traditional birth attendants who agreed to participate in this study based on anonymity that all information provided will be strictly confidential. All transcripts were anonymized including the extracts that support the study's findings. Transcripts are available for research purposes only in line with the approved research protocol. This is in fulfilment of the participants confidentiality as indicated in the approved research protocol from the Humanities and Social Sciences Research Ethics Committee [HSSREC/00000924/2019] and Osun state Ministry of Health [OSHREC/PRS/569T/157] dated 20th April 2020 and 19th August 2019 respectively.

Funding: The corresponding author received student scholarship from the University of KwaZulu-Natal, South Africa through the College of Health Sciences student Scholarship.

Competing interests: The authors have no competing interests of any form as regards this study.

Author Contributions

Conceptualization: Rosemary Omolara Fafowora, Sinegugu Evidence Duma.

Formal analysis: Rosemary Omolara Fafowora, Sinegugu Duma.

Investigation: Rosemary Omolara Fafowora.

Methodology: Rosemary Omolara Fafowora.

Supervision: Sinegugu Evidence Duma.

Validation: Sinegugu Evidence Duma.

Writing - original draft: Rosemary Omolara Fafowora

Writing -reviewing & editing: Sinegugu Evidence Duma

Formatted: Font: Bold

References

1. World Health Organization. Female Genital Mutilation. Fact sheet. 2020 Retrieved February 20, 2022, from http://www.who.int/mediacentre/factsheets/fs241/en/
2. Waigwa S, Doos L, Bradbury-Jones, Taylor J. "Effectiveness of health education as an intervention designed to prevent female genital mutilation/cutting (FGM/C): a systematic review." Reproductive health 2018; **15**(1): 62.
3. United Nations, 2015. 'The Millennium Development Goals Report 2015', pg. 38. http://www.un.org/millenniumgoals/2015_MDG_Report/pdf/MDG%202015
4. UNICEF. Female Genital Mutilation/Cutting: A Global Concern UNICEF, New York, 2016.
5. World Health Organization. Female genital mutilation: Key facts. 2018 [cited 1 May 2022]. https://www.who.int/news-room/fact-sheets/detail/female-genital-mutilation
6. UNFPA-UNICEF Joint Programme on the Elimination of Female Genital Mutilation (2019).
7. Nigeria Demographic and Health Survey (2013). National Population Commission. Federal Republic of Nigeria.
8. Berg RC, Denison E. Health Care for Women International A Tradition in Transition: Factors Perpetuating and Hindering the Continuance of Female Genital Mutilation / Cutting (FGM / C) Summarized in a Systematic Review A Tradition in Transition: Factors Perpetuating Genital. 2013;9332(October 2017).

9. Yaya, S. and B. Ghose (2018). "Female Genital Mutilation in Nigeria: A Persisting Challenge for Women's Rights." Social Sciences 7(12): 244.
10. Reisel, D. and S. M. Creighton (2015). "Long term health consequences of Female Genital Mutilation (FGM)." Maturitas 80(1): 48-51.
11. Violence against Persons (Prohibition) Act,2015, Laws of the Federation of Nigeria
12. National Population Commission of Nigeria. Nigeria Population and Housing Census 2006. Abuja, Nigeria: National Population Commission of Nigeria.
13. Omole, F.K., Lukman, Y. and Baki, A.I. (2014), "Analysis of market typology and functions in the development of Osun state, Nigeria", International Journal of Development and Sustainability, Vol. 3 No. 1, pp. 55-69
14. Barret H. Alhassan Y. The REPLACE Community Readiness to End FGM Assessment. Practical Handbook (based on REPLACE Approach www.replacefgm2.eu)2016, Coventry university, UK.
15. McCoy H.V, Malow R., Edwards R.W., Thurland A., Rosenberg R. A Strategy for Improving Community Effectiveness of HIV/AIDS Intervention Design: The Community Readiness Model in the Caribbean, Substance Use & Misuse,2007; 42:10, 1579-1592, DOI: 10.1080/10826080701212535
16. Borrayo, E. A. (2007). "Using a community readiness model to help overcome breast health disparities among US Latinas." Substance use & misuse **42**(4): 603-619.

17. Duma, S. Women's journey of recovery from sexual assault trauma: a grounded theory.2006.

18. Van Teijlingen, E R, Hundley V. "The importance of pilot studies."(2001).

19. Braun V, Clarke V. Thematic analysis. in H. Cooper, P. M. Camic, D. L. Long, A. T. Panter, D. Rindskopf, & K. J. Sher (Eds.), *APA handbook of research methods in psychology, 2012;Vol. 2. Research designs: Quantitative, qualitative, neuropsychological, and biological* (pp. 57–71). American Psychological Association. https://doi.org/10.1037/13620-004

20. Louis Cohen L, Manion L, Morrison K. Action research. Research methods in education, Routledge 2013:368-385.

21. Polit, D. and C. Beck. "Essentials of nursing research." Ethics2012; **23**(2).

22. Fawcett J, Garity J. "Chapter 1: Research and evidence-based nursing practice." Evaluating research for evidence-based nursing: (2009); 3-20.

23. Dunn F. Is It Possible to End Female Circumcision in Africa? Clin J Obstet Gynecol. 2021;1: 007-013. https://doi.org/10.29328/journal.cjog.1001002

24. Sakeah E, Debpuur C, Aborigo RA, Oduro AR, Sakeah JK, Moyer CA (2019) Persistent female genital mutilation despite its illegality: Narratives from women and women in northern Ghana. PLoS ONE 14(14):e0214923. https://doi.org/10.1371 journal pone 0214923.Obi A.I., Adesoye O., LabiranA. Female Genital Mutilation: Attitude and Practices among Women in Okada Community, Edo State JMBR: A Peer-review Journal of Biomedical Sciences December 2015, Vol. 14 No. 2 pp 138-150

24.

25. Andarge, MasreshaYazew. *The difficulties of ending Female Genital Mutilation (FGM): Case of Afar pastoralist communities in*

Formatted: English (United States)

Ethiopia. *Social Policy for Development (SPD) (2014, December 12)*. Retrieved from http://hdl.handle.net/2105/17487

26. Ibrahim I.A, Oyeyemi A.S, Ekine A.A. Knowledge, Attitude and Practice of Female Genital Mutilation among Doctors and Nurses in Bayelsa state, Niger-Delta of Nigeria. International Journal of Medicine and Biomedical Research Volume 2 Issue 1 January – April 2013
27. UNFPA. 2020. 'Impact of the COVID-19 Pandemic on Family Planning and Ending Gender-Based Violence, Female Genital Mutilation and Child Marriage.' Interim Technical Note.

The political Map of Osun State, Nigeria ~~showing the various towns~~

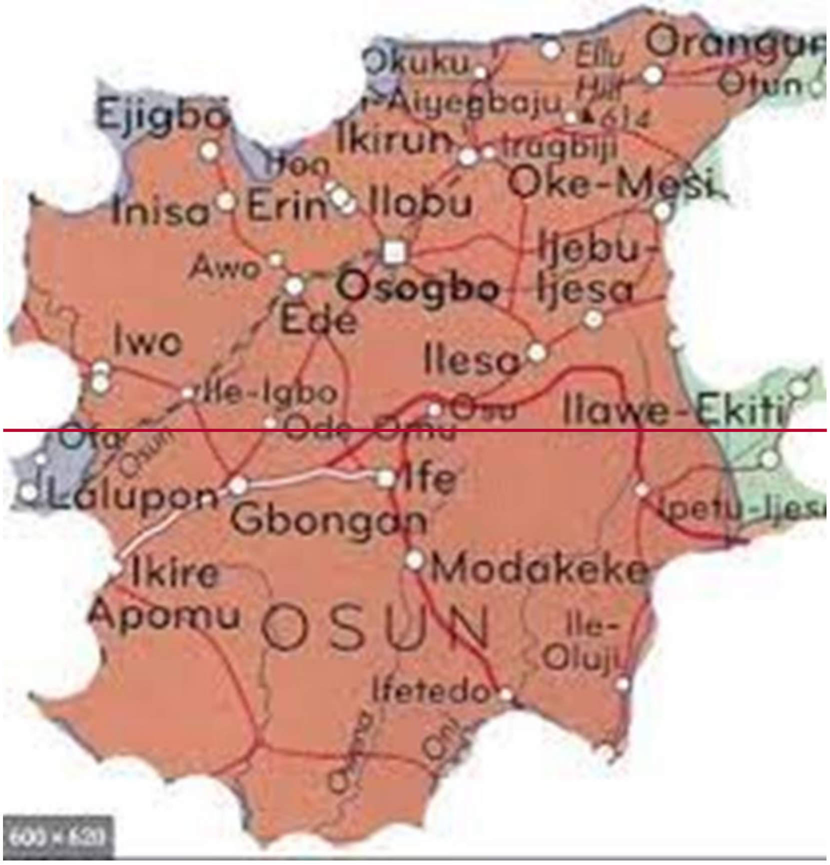

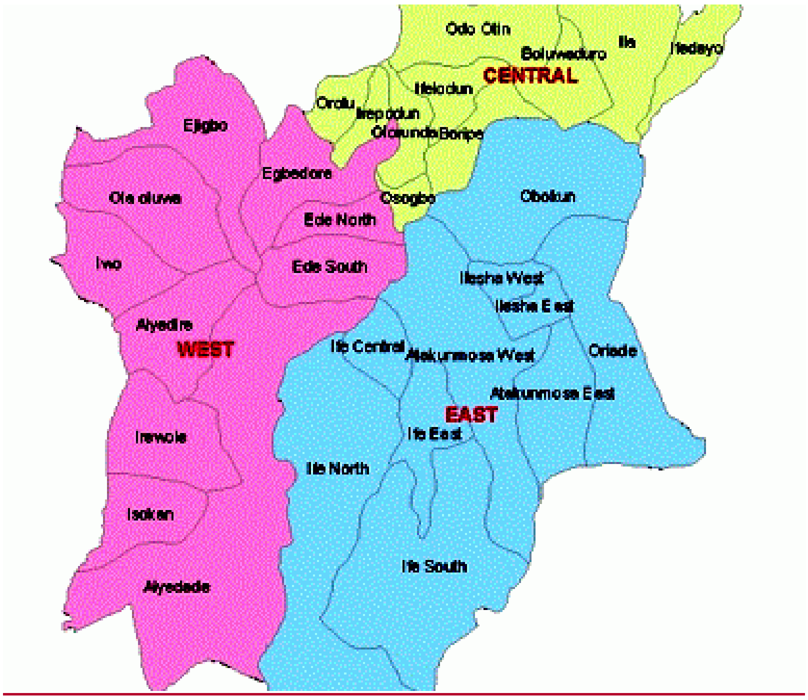

Adapted from https://dawncommission.org

Full Title:

'It Is What Was Handed Over to Us as Our Heritage and Must Not Be Taken Away Just Like That': Traditional Birth Attendants' Attitudes Towards the Elimination of Intergenerational Female Genital Mutilation/Cutting in Osun State, Nigeria.

Short Title:

Eliminating Intergenerational Female Genital Mutilation/Cutting in Osun State, Nigeria.

Authors:

ROSEMARY OMOLARA FAFOWORA[1¶*ID] orcid.org/0000-0003-2968-3784

SINEGUGU EVIDENCE DUMA[2¶ID] orcid.org/0000-0002-2489-8770

[1]Department of Nursing, School of Nursing and Public Health, College of Health Sciences, Howard Campus, University of KwaZulu-Natal, Durban, South Africa.

[2]Department of Nursing, School of Nursing and Public Health, College of Health Sciences, Howard Campus, University of KwaZulu-Natal, Durban, South Africa.

*Corresponding author

E-mail:rosemary.olagunju@gmail.com (RO)

~~¶The authors equally contributed to this work.~~

Abstract

Background

Female genital mutilation/cutting is a harmful practice that violates the sexual and reproductive health rights of women and girls. The practice is often perpetrated directly or indirectly from one generation to another as a way of preserving culture, thus making it difficult to tackle using ordinary prevention interventions. The purpose of the study was to assess the attitude of the traditional birth attendants as community leaders towards the elimination of intergenerational female genital mutilation/cutting (FGM/C) of girls and to determine their level of readiness and preparedness towards achieving it in Osun State, Nigeria.

Methodology/Results

A qualitative research design, using the adapted REPLACE community readiness tool to end female genital mutilation/cutting interview guide, was used to individually interview eight traditional birth attendants who were identified through purposive sampling method as community leaders and key informants. Thematic Analysis was used to analyze the data which yielded female genital mutilation/cutting as traditional heritage, defiance against government efforts and debunking all "western" information about dangers of female genital mutilation to women as lies as findings. These findings are worrisome because they are an indication of the community leaders' level of no readiness for interventions for elimination of intergenerational female genital mutilation/cutting in Osun state, Nigeria.

Conclusion

We recommend that the government, policy makers and health professionals elicit and carefully consider the views of the community leaders such as traditional birth attendants who are custodians of the female genital mutilation/cutting before introducing measures to eliminate it in the traditional communities. This may include having non-confrontational dialogue between all stakeholders, for understanding of the dangers and benefits of female genital mutilation/cutting to the nation which can be shared from everyone for best solutions to be found.

Key Words: Replace Community Readiness Tool, Intergenerational Female Genital Mutilation/Cutting, Traditional Birth Attendant

Introduction

Female genital mutilation/cutting (FGM/C) is a public health problem globally. It comprises all procedures that involve partial or total removal of the external female genitalia, or other injury to the female genital organs for non-medical reasons. It is also referred to as multiple procedures on the female genital organs for non-medical reasons [1]. FGM/C has been described as a harmful practice that violates the sexual and reproductive health rights of women and girls [2,3]. It is estimated that more than 200 million girls and women alive today have undergone FGM/C across 30 countries including the Middle East, Asia, and Africa [4]. Of this, about 44 million are girls under the age of 15 years [5]. It is further projected that more than 3 million girls and women are currently at risk of FGM/C worldwide [4]. The current estimates of 3.9 million girls mutilated annually will also rise to 4.6 million by 2030 because there is an increase in the population growth in the communities that still practice FGM/C [6]. It is reported that 20 million girls and women are estimated to have undergone

FGM/C in Nigeria, with Osun state, where the study was conducted, having reached the highest prevalence of 76.6% of FGM/C [7].

In Nigeria, FGM/C is a deeply entrenched custom that is commonly performed by the traditional birth attendants (TBAs) who claim it as a heritage which confers honor on a woman and her family, a rite of passage to womanhood, preservation of chastity, enhancement of fertility, and prevention of death of the child during delivery among its benefits [8].

Despite its cultural importance, FGM/C has received considerable criticism worldwide because of its potential risk for various forms of health complications [9]. The short term and physical health complication of FGM/C includes but are not limited to pain, risk of hemorrhage, shock, tetanus/sepsis, inability to urinate, damage to adjacent organs such as the rectum and urinary tract, dislocation and fracture of the pelvic bones and the head of the femur due to struggle while being restrained, open sore in the genital region, risk of HIV/AIDS or Hepatitis B. While the long term and reproductive health consequences of FGM/C include difficulty in passing menstrual blood, vaginal fistula, painful sexual intercourse, and difficulty in passing urine which can lead to recurrent urinary tract infection, clitoral cyst, infertility, an increased risk of childbirth complication and newborn death, need for future surgeries to allow for sexual intercourse and childbirth, psychological trauma, and risk of death from hemorrhage or infection [10].

Furthermore, sexuality is an equal right that both males and females must benefit from. While men are being circumcised for health and religious reasons, women and girls are cut deliberately to remove their sexual feelings and subjugate them to the dictates of their men counterpart, and it is done without any consent from them.

In 2005, the Violence Against Persons (Prohibition) Act 2015 was passed to eliminate the FGM/C practice in Nigeria. Since then, several legal, community based, and health-based strategies had been instituted to eliminate the FGM/C practice [11]. These strategies were met with strong opposition from the TBAs, who remain the self-proclaimed custodians of the traditional responsibility of FGM/C from time immemorial, especially in Osun State, Nigeria. The TBAs believe in the supremacy of FGM/C as the traditional heritage, which is concerned about the wellbeing of the people, that it predates modern medicine and therefore should not be stopped. This cultural belief has facilitated the intergenerational preservation of FGM/C and explains their attitude towards the elimination of its practice. The intergenerational preservation of FGM/C is reflected on most women's belief that the elimination of FGM/C is tantamount to defaulting from their religion or traditions which could breed calamity for them and their offspring. These beliefs have been passed down from one generation to the other and contribute to the high prevalence of FGM/C in Osun State, Nigeria, where it has become major public health issue that requires public health approach-based prevention interventions. Because of their influence on the intergenerational preservation of FGM/C, we identified the TBAs as community leaders who are key towards the development and implementation of public health prevention intervention for FGM/C in Osun State, Nigeria. The purpose of the study was to assess the attitude of the TBAs as community leaders towards the elimination of intergenerational FGM/C of girls in Osun State, Nigeria, in order to determine their level of readiness and preparedness towards achieving its elimination.

Materials and Methods

Study setting

Osun State is an inland state in South-West Nigeria with a population of 3,423,535 [12]. The present Osun State was created in 1991 from the old Oyo State. The state shares boundary

with Kwara State in the North, Oyo in the West, Ogun and Ondo in the South and Ekiti State on East. The state capital is Osogbo, which is a local government area on its own. In all, a total of thirty local government council areas and Ife east area council constitute the state. Among the major towns in the state are Ilesha, Ife, Ede, Ikirun, Ikire, Ejigbo and Osogbo [13]. Before this time, the state was part of the old Oyo State. Osun State experiences a tropical climate while the local vegetation is the lowland rainforest type.

Indigenes of Osun State belong to the Yoruba race and comprise the Oyo, Ife, Ijesa, Igbomina and Osun. Many non-indigenes and foreigners also reside in the state and live together in harmony. Yoruba and English are the languages used for official and business transactions. The people have a rich cultural heritage that is demonstrated in all spheres of their life including the performance of FGM/C. Their culture finds expression in their arts, literature, music, and other social activities.

This study was conducted among the Yoruba TBAs residing across the three senatorial districts in Osun State, Nigeria. The Yoruba people have a rich cultural heritage that is demonstrated in all spheres of their life including the performance of FGM/C in the Osun State, Nigeria.

Research design

A qualitative research design, using the adapted REPLACE community readiness to end FGM/C interview guide [14] was used to assess the attitudes of the TBAs as community leaders towards elimination of intergenerational FGM/C to determine their level of readiness and preparedness towards achieving its elimination in Osun State, Nigeria. This was the most suitable approach because it focuses on social and cultural contexts to understand how a problem is perceived and whether taking some type of action is seen as needed by community leaders to change the problem [15, 16]. The adapted REPLACE community readiness to end

FGM/C interview guide [14] was translated to the local (Yoruba) language by a bilingual expert in Yoruba and English who is also a qualitative researcher for comparison of the language translation to avoid the loss of meaning in the translated interview guides. The same person was used for translating transcripts to English.

Sampling and recruitment of participants

Purposive sampling method was used select eight TBAs whom we were regarded as community leaders and key informants with rich information regarding intergenerational FGM/C in Osun State, Nigeria. The inclusion criteria for recruitment and participation in the study included only TBAs who were formally recognized as leaders in performing FGM/C, who had been previously involved in any of the Government's organized seminars or workshops on FGM/C elimination and or prevention intervention in Osun State, irrespective of the duration of the practice. The selection and recruitment of the participants commenced once the ethical approval from the Humanities and Social Sciences Research Ethics Committee [HSSREC/00000924/2019] and Osun state Ministry of Health [OSHREC/PRS/569T/157] was obtained. Meetings were held with the nurses in the maternity units across the three senatorial districts as gatekeepers to inform them about the purpose of the study and to seek their permission to meet the TBAs working in their respective Units. A meeting with TBAs was held where the purpose of the study, the research eligibility criteria and request for voluntary participation from those who meet the inclusion criteria were discussed. Those who showed interest were recruited and were given the information sheet and the informed consent form to complete and sign. Arrangements for the interview session were then made by allowing the potential participants to choose suitable date and time within the first 7 days after the participants have given their consent. Before the commencement of the interview, the purpose of the study, and permission to audio record the interview sessions

were explained again to the participants in the local language (Yoruba). A total sample size of eight traditional birth attendants as community leaders and key informants were recruited in the study. This small sample size is in line with the REPLACE community readiness tool, which suggests 6-12 key informants as sufficient to address issues in the community [14].

Pilot study

The first two TBAs out of the sample size were used for pilot study to ensure that the interview guide questions were easy and yielded the necessary data. The data generated from the pilot study were included in the main study data analysis. Contamination of data was not considered to be a concern because in qualitative studies data collection is progressive and the data from pilot studies can be used for the main study [17,18].

Data collection

Data collection and data analysis were conducted concurrently between October 2020 and March 2021. All COVID-19 precaution protocols like using the face mask, maintaining social distancing and hand sanitizing were observed during the interviews. In-depth interviews were conducted with the individual TBAs in their homes by the first author in Yoruba, the local language, using the interview guide adapted from the REPLACE Community Readiness to End FGM/C Assessment tool [14]. The interviews were audio recorded and transcribed within 24 hours of recording when information was still fresh in the researcher's memory for data analysis purposes. Each interview lasted between 45-60 minutes to complete thus allowing each TBA to adequately share their views as attitudes towards elimination of intergenerational FGM/C in Osun state. Nigeria. The transcripts were stored in word document formats on a computer with encrypted password and was backed up to an external hard drive to prevent data loss in line with the University's principles for data storage.

Data analysis

The six steps, namely: familiarization, coding, searching for themes, reviewing themes, defining, and naming themes, and writing up as described by [19] were used to analyze the transcribed data.

The first author went through the transcripts reading and re-reading them to familiarise herself and make sense of the data. Notes were taken and preliminary ideas for codes were marked. In order to start organizing the data into meaningful groups, codes were assigned to briefly describe something interesting that was said by the participants. The first author grouped the newly generated codes with same meaning together to form themes. The second author, who also doubled as the inter coder at this stage, also did the same thing. The generated themes by the first and second authors were scrutinized by both of them to ensure that they were useful and accurate representation of the data by discarding some themes and creating new ones from the transcribed data. The first author then formulated the meaning of each theme so that the data can be understood. At this stage, appropriate naming of themes was made to succinctly interpret the data collected.

Ethical Permission

All ethical principles were adhered to including, consent form for voluntary participation, ethical approval from the Humanities and Social Sciences Research Ethics Committee [HSSREC/00000924/2019] and Osun state Ministry of Health [OSHREC/PRS/569T/157] were obtained.

The names assigned to each of the participants are not their actual names as we are aware of the ethical implication. However, using pseudonyms instead of participants' actual names ensured confidentiality and anonymity.

Measure to ensure trustworthiness

Measures to ensure trustworthiness and rigor of the findings were applied through the criteria of credibility, confirmability, dependability, and transferability [20].

To ensure credibility, comprehensive field notes, noting gesture and other non-verbal ques observed during the interviews were recorded and analyzed. Audit trail of all research processes were recorded. Analysis of the data gathered reflected a neutral interpretation of the data rather than the researcher's view as recommended by [21,22]. This was achieved by conducting a member checking on data whereby the analyzed data was summarized and provided to the participants for them to validate and determine the accuracy of the data as their views. For dependability and transferability, an audit trail was kept and the comprehensive and rich details of the study setting, and the socio-demographic characteristics of the participants are presented. To further establish trust and confidence in the findings, the authors compared their individually generated themes from the raw data to ensure that they were useful and accurate representation of the data by discarding some themes and creating new ones from the transcribed data. The senior author and research supervisor reviewed the themes against raw data and helped in refining these to reflect participants' views to enhance confirmability. Common issues recur and main themes were identified to summarize all the views that have been captured in the data. One theme and three subthemes emerged that revealed the defensive attitude of the TBAs towards the elimination of intergenerational FGM/C of girls in Osun state, Nigeria.

Results

Eight (8) TBAs, comprising of three females and five males participated in the study on TBA's attitude towards the elimination of intergenerational FGM/C of girls in Osun State, Nigeria.

Table 1: Participants' Demographic Characteristics

Participant	Age	Gender	Religion	Educational level
Lukman	45	Male	Islam	Tertiary education
Abike	70	Female	Islam	Primary education
Adeyemi	53	Male	Islam	Tertiary education
Olalekan	86	Male	Islam	Primary education
Mojirola	63	Female	Christian	Secondary education
Amope	80	Female	Islam	Primary education

Fadipe	73	Male	Islam	Secondary education
Adebayo	75	Male	Islam	Primary education

These demographics reflects the TBAs in Osun State where the person who becomes a TBA is culturally determined through the lineage of the traditional circumcisers. It is a taboo for a woman who is married to perform FGM/C as TBA, but she can still retain her knives for performing FGM/C on her own children only (personal conversation with Mr Adeyemi, a male participant to the study, December 2020). This explains why most participants were male TBAs in the study as reflected in Table 1. Their ages ranged between 45 and 86 years. Seven of them practiced Islam and only one was Christian as a religion.

Defensive attitude towards elimination of intergenerational FGM/C

Analyzed data yielded "Defensive attitude towards elimination of FGM/C" as the main theme in the TBAs' attitude towards the elimination of intergenerational FGM/C of girls in Osun State, Nigeria. Three subthemes, including (i) FGM/C as a traditional heritage, (ii) Defiance against government's efforts to eliminate intergenerational FGM/C and Debunking all "western" information about dangers of FGM/C as lies emerged under the main theme.

FGM/C as traditional heritage

The FGM/C as a traditional heritage sub theme emerged from data related TBAs' defensive attitudes towards elimination of intergenerational FGM/C as traditional and cultural beliefs

that the practice is the identity and the only link between the current and past among generations as described in the following extracts.

The response from the respondent below upheld the fact that FGMC is their traditional heritage that cannot be stopped in their lifetime.

I am talking about our traditional heritage here. It can’t be stopped; it can’t stop now. The law, civilization coupled with ignorance is stopping people, but it cannot be stopped. [Lukman, Male, Age: 45, Tertiary Education]

Another respondent also mentioned that FGM/C is a traditional heritage that was handed down by their forefathers.

I think all they are saying about stopping of our tradition is done out of civilization and hatred, hatred of us and our traditional heritage. What has been established by our forefathers cannot perish. It is just like asking us to abandon our language because of civilization. There is no where we meet that we can do without speaking our language. Girl child circumcision cannot perish. [Abike, Female, Age:70, Primary Education]

Further, data under this subtheme showed that TBAsconsiderate as their primary responsibility to prevent intergenerational practice of FGM/C from extinction as depicted in the following extract.

Girl circumcision has been in existence from time immemorial. Therefore, it can be described as our cultural identity that was transmitted from generation to generation that no one is ready to allow it to die down in his or her own time, even if your government says it is bad for us. [Fadipe, Male, Age:73, Secondary Education]

It was also reported as shown in the extract below that the TBAs have a duty to protect FGM/C being their inheritance.

But you know, it is our heritage which must be promoted. It is not that it really makes more money for us. Do you understand me? It is not what the TBAs are eating from. But it is what was handed over to us as our heritage and must not be taken away just like that! [Abike, Female, Age: 70, Primary Education].

Defiance against government's efforts to eliminate FGM/C

This subtheme emerged from data related to the defiant attitudes of the TBAs towards seminars and workshops that had been previously arranged by government and other organizations in the country with the aim of highlighting the disadvantages of intergenerational FGM/C proposals towards eliminating FGM/C as described in the following extracts.

The extract below shows that despite the attendance at the seminar organized for the TBA by the government, stopping FGMC is going to be a disadvantage to the women because their sexual urge may not be easily controlled as we are seeing around us.

I attended the workshops about this, but I was and am still convinced that the disadvantages of government's effort to stopping FGM/C will be enormous for our women. See what is happening nowadays in our communities. No one will be able to control these girls. We must fight anyone including the government to protect our girls from promiscuity. [Lukman, Male, Age: 45, Tertiary Education]

The following extract upholds the submission by the TBAs that FGM/C prevents promiscuity which explains why the tradition has been successfully continued.

No, I don't agree with the government's decision. The result will be very bad. As for me, I did it for all my children both boys and girls. None of my children are promiscuous because I circumcised them and took care of them all. I buried what was cut and properly watered the

soil where I buried it so that chicken could not be able to unearth it. Otherwise, if unearthed, the girl child will be promiscuous. We are very careful about these things so that no one can come back and say FGM/C doesn't work. [Amope, Female, Age: 80, Primary Education].

A respondent bluntly declared that she will never support the government in stopping FGM/C as depicted in the following extract.

Ah! I am not in support of the government stopping the girl child circumcision. That is our campaign, and I am not hiding it. At least, it will not happen in my time. [Abike, Female, Age 70, Primary Education]

Elimination of FGM/C is described as an advantage to the girl child that no one would want to support as shown in the quote below.

Personally, I don't agree with government's reasons to stop girl child circumcision. I have said it earlier that, based on my experience and the level of my education, and coming from a family that is traditionally practicing FGM/C, I do not see it being stopped by anyone, even the government or anyone else, not now. I did it (FGM/C) for my girls and there is no problem associated with it, I don't think it should be stopped. It will be a great disadvantage if anyone could agree with government. [Lukman, Male, Age: 45, Tertiary Education].

Debunking all "Western" information about dangers of FGM/C to women as lies

This subtheme emerged from data ~~relatedto~~related to TBAs efforts in debunking most of the information and claims about the benefits of eliminating intergenerational FGM/C as just government's efforts to brainwash people, against the practice of FGM/C as depicted in the quotes below.

The information about the girls having reproductive challenges later in life was debunked by the respondent as shown in the quote below.

The girls that I circumcised did not have any problem. Even when grown up, these girls do not develop any of the problems we were told resulted from FGM/C, which we were now told can be associated with FGM/C. The girls are now big women and are all alive and doing well in their families. They got married to nice men and have lively children. [Amope, Female, Age 80, Primary Education]

The extract below shows that the respondent disagrees with the record of death sequel to FGM/C. He said that the girls he circumcised as infants are still alive today.

Every girl that we have circumcised is still alive even today. No child can die in the hand of circumcision surgeon and all our girls were circumcised. Anyone who say we put our girls in danger is a liar. My wife is circumcised, all my girls are circumcised and alive. They must stop spreading lies about FGM/C. [Olalekan, Male, Age 86, Primary Education]

In addition, the likelihood of lack of enjoyment during sex was further refuted but rather held that circumcised women enjoy sex more as shown in the quote below.

That debunked FGM/C leads to women not enjoying sex is one of the lies the government is peddling about girl circumcision. But they have forgotten that it's only a small part of the clitoris that is cut and not the whole clitoris. it is not true at all. [Adeyemi, Male, Age 53, Tertiary Education]

It is only a false assumption that circumcised women do not enjoy sex. When a circumcised woman has sexual intercourse with men, she will glue to him as if they tie penis and vagina together, in that way, they both enjoy each other nicely [Olalekan, Male Age: 86, Primary Education]

Furthermore, the views that FGM/C have complications was refuted as just rumors as captured by the following extracts.

There is nothing like transmission of sexual diseases by FGMC, bleeding and all so many other things that are being said about female circumcision. It is safe and cultural to do it (circumcision). [Mojirola, Female, Age 63, Secondary Education]

We were also told that female circumcision causes sexual transmitted diseases / infections which is not true at all! Honestly, I don't know what the government want to gain from spreading such lies for stopping it. Female circumcision does not have any negative side effects as it is being peddled around. [Amope, Female, Age 80, Primary Education].

Discussion

The study was conducted to assess TBAs' attitudes towards the elimination of intergenerational FGM/C of girls in Osun state, Nigeria in order to determine their level of readiness and preparedness towards achieving its elimination. Attitudes towards intergenerational FGM/C can vary among TBAs in Osun state, Nigeria as they represent a diverse group with different beliefs and perspectives. It is important to note that TBAs attitude may also reflect a broader societal attitudes and cultural norms prevalent that is prevalent in the region.

In recent years, there have been significant efforts to promote the elimination of FGM/C in Nigeria. These efforts include education and awareness campaigns, legal measures, and community engagement programs. However, despite these efforts, report shows that the TBAs are still hold on to the traditional beliefs and still support the practice.

Our findings show a deep defensive attitude towards elimination of FGM/C among the TBAs as community leaders and custodians of the intergenerational FGM/C which is reflected in the participants views that FGM/C is their traditional heritage and its elimination can be

equated to eliminating their cultural identity. This finding conforms with the conclusion of Dunn [2018] that the cultural identity of the Africans is being eliminated by current civilization in their research article that shed light on how possible it is to end female circumcision in Africa. [23] and SObi, Adesoye, Labiranakeah et al (2019) that also reportidentified tradition/culture as one of the contributory factors to the persistent practice of FGM/C in their ed in their studyresearch article thatwhich discussed the narratives from women and men in northern Ghana about the persistent in FGM/C despite its illegality, culture/tradition is the greatest human inheritance, since it contributes to the preservation of history and identity of any race, thus confirming the TBAs' beliefs about their responsibility to preserve intergenerational FGM/C as their traditional heritage despite all its disadvantages to women, their families, and communities [24]. The TBAs were of the belief that FGM/C is a cultural tradition that must not be taken away the way others were eroded. Besides, many of them also condemn the insincerity on the side of the colonial masters that gradually stripped their subjects off their cultural heritage. -Despite its cultural importance, however, FGM/C has received considerable criticism worldwide because of its potential risk for various forms of health complications. This was supported by Yaya and Ghose, 2018 in their study with the aims of measuring the trends of FGM/C in Nigeria and its persisting challenge for women's right where various complications of FGM/C were identified, among which are clitoral cyst formation, hemorrhage, fistula, obstetric complications, urinary infection and retention, vagina tears and psychological trauma [9].

Findings from this study also depicted a strong defiance against the government's prevention interventions as indicated in the defiance shown by those TBAs who had attended seminars and workshops organised by the government on the elimination of intergenerational FGM/C, thus inconsistent with the previous findings by Andarge Masresha Yazew (2014) where factors contributing to the difficulties of ending FGM/C in Afar pastoralist communities in Ethiopia were [identified [255]. who It was concludednoted that unsuccessful elimination of intergenerational FGM/C was due the government's inability to involve the major community stakeholders such as the TBAs.

Therefore, due to the TBAs' defiance against governments' efforts to eliminate FGM/C, there is a continuous non-alignment between government agencies and the views and beliefs of those responsible for promotion of historic traditions (such as FGM/C) always ending up in disagreement, if not properly handled. This is similar to what Dunn [23] mentioned in an article that discussed the possibility to end FGM/C. According to the TBAs~~'~~, the~~ir~~ ~~voices and understanding of their attitudes towards abandoning their age long custom, and not only inviting them to attend seminars or workshops on~~ government should see them as stakeholders in the elimination of FGM/C, which is important in determining the community's readiness for development and implementation of interventions for eliminating intergenerational FGM/C of girls. ~~This is in line with the research paper presented by Masresha Yazew Andarge (2014) where the difficulties of ending FGM/C in Afar pastoralist communities in Ethiopia were identified (26). It was suggested in the that to eradicate FGM/C,~~ Recognising ~~it is important to understand how deep the cultural influence is in continuing the tradition in the name of sustaining culture.~~ the profound extent of cultural influence that underlies the perpetuation of traditions as it plays a pivotal role in preserving and safeguarding cultural heritage cannot be underestimated ~~The TBAs interviewed insisted that for~~ by the government ~~to be~~in the successful ~~in the~~ campaign against FGM/C~~, they must be carri.~~

Formatted: Underline, Font color: Blue

Formatted: Underline, Font color: Blue

Formatted: Underline, Font color: Blue

Formatted: Underline, Font color: Blue

Formatted: Underline, Font color: Blue

Formatted: Underline, Font color: Blue

Formatted: Underline, Font color: Blue

Formatted: Underline, Font color: Blue

Formatted: Underline, Font color: Blue

Most of the information and claims about the benefits of eliminating FGM/C were debunked by the TBAs as shown from the analysis. The~~y~~ TBAs claimed that it is just government's efforts to brainwash people which is disputed by previous report which supports the government's assertion on the benefits of eliminating intergenerational FGM/C. This finding is also in contrast with the conclusion of Ibrahim I.A et al. 2013 in their study in Yenegoa, Bayelsa, Nigeria [26] where it was ~~[26] who~~ reported that more patients with complications

of FGM/C such as difficult delivery, perineal laceration, hemorrhage, scar/keloid etc. were seen by nurses/midwives than doctors. However, limited or lack knowledge about complications may be reflected as negative attitudes towards prevention or elimination of intergenerational FGM/C in their lifetime which may be the case of the TBAs claiming that the complications are all lies and an effort the white people to rip them of their age long tradition. On the other hand, they hold the viewpoint that the complications from FGM/C are arising due to the participation of individuals who are not typically burdened with such responsibilities.in Yenegoa, Bayelsa, Nigeria [26].

The TBAs are of the opinion that the involvement of people who are not traditionally entitled to circumcision and are not knowledgeable enough are the ones causing the complications as stated by the government. The TBAs' debunking of all "Western" information about dangers of FGM/C to women as lies may be due to their lack of knowledge of the dangers and complications of FGM/C. Similar findings were reported by [26] who concluded limited knowledge about complications may be reflected as negative attitudes towards prevention or elimination of intergenerational FGM/C in their lifetime.

Our findings show that FGM/C is going to continue silently in Osun state, Nigeria despite government's efforts to eliminate intergenerational FGM/C. Apart from factors raised above, COVID 19 pandemic has had various impacts on global health and welfare, including the continuation of harmful practices such as FGM/C. The pandemic has presented challenges in combating FGM/C and protecting girls and women at risk.

This is confirming the report by the UNFPA (2020) which estimated that because of population growth, additional 4.6 million girls are projected to be at risk of undergoing female genital mutilation by 2030 [27]. In addition to that, it was also projected that 2 million

cases of FGM/C could be recorded over the next decade due to COVID-19 alone [27] . . It was also reported that the outbreak of COVID-19 between 2020 and 2022 aggravated the vulnerability of girls and women who are at risk of FGM/C which indicated that 2 million cases of FGM/C could be recorded over the next decade due to COVID-19 alone [27].

Strength and Limitation

Using the adapted REPLACE community readiness to end FGM/C interview guide [14] was used to assess the attitudes of the TBAs as community leaders towards elimination of intergenerational FGM/C to determine their level of readiness and preparedness towards achieving its elimination in Osun State, Nigeria. This approach was found suitable because it focuses on social and cultural contexts to understand how a problem is perceived and whether taking some type of action is seen as needed by community leaders to change the problem [15, 16].

Several limitations were encountered in this study. Using local language to collect data could have distorted the presentation of the questions but back and forth translation of the questions minimized the language bias.

As a female interviewer, there is likelihood of introducing bias during the interview on the FGM/C. However, conscious efforts were made to ensure that the questions were asked in a way that does not influence the response of the participants, the analysis and the interpretation of data collected.

Another limitation is the reliability of the responses provided by the TBAs because they may not provide an honest response about their beliefs especially now that there have been campaigns against FGM, and there is legal repercussions to having practiced FGM

Formatted: Font: Not Bold

Formatted: Font: (Default) Times New Roman, 12 pt

Formatted: Font: 12 pt

~~Using local language to collect data could have distorted the presentation of the questions but back and forth translation of the questions minimized the language bias.~~

Conclusion

The current defensive attitudes of the TBAs as community leaders are worrisome because they are an indication of the community leaders' level of no readiness for intervention programmes for the elimination of intergenerational FGM/C in Osun state, Nigeria.We recommend that the government, policy makers and health professionals elicit and carefully consider the views of the community leaders such as TBAs who are custodians of the FGM/C before introducing measures to eliminate FGM/C in traditional communities. This may include having non-confrontational dialogue between all stakeholders for understanding of the dangers and benefits of FGM/C to the nation which can be shared from everyone for best solutions to be found.

Acknowledgement

Our sincere appreciation goes to the management of the Ministry of Health in Osun state Nigeria and University of KwaZulu-Natal for granting us the ethical approval to carry out the study. We also thank the Nurses and the Traditional Birth Attendants for creating time to grant the interviews.

Data Availability Statement: All relevant data are within the paper and its supporting information file. ~~This is a qualitative study that involves 8 traditional birth attendants who agreed to participate in this study based on anonymity that all information provided will be~~

strictly confidential. All transcripts were anonymized including the extracts that support the study's findings. Transcripts are available for research purposes only in line with the approved research protocol. This is in fulfilment of the participants confidentiality as indicated in the approved research protocol from the Humanities and Social Sciences Research Ethics Committee [HSSREC/00000924/2019] and Osun state Ministry of Health [OSHREC/PRS/569T/157] dated 20th April 2020 and 19th August 2019 respectively.

Funding: The corresponding author(s) received student scholarship from the University of KwaZulu-Natal, South Africa through the College of Health Sciences student Scholarship.no specific funding for this work.

Competing interests: The authors have no competing interests of any form as regards this study.

Author Contributions

Conceptualization: Rosemary Omolara Fafowora, Sinegugu Evidence Duma.

Formal analysis: Rosemary Omolara Fafowora.

Investigation: Rosemary Omolara Fafowora.

Methodology: Rosemary Omolara Fafowora.

Supervision: Sinegugu Evidence Duma.

Validation: Sinegugu Evidence Duma.

Writing - original draft: Rosemary Omolara Fafowora

Writing -reviewing & editing: Sinegugu Evidence Duma

Formatted: Font: Bold

References

1. World Health Organization. Female Genital Mutilation. Fact sheet. 2020 Retrieved February 20, 2022, from http://www.who.int/mediacentre/factsheets/fs241/en/
2. Waigwa S, Doos L, Bradbury-Jones, Taylor J. "Effectiveness of health education as an intervention designed to prevent female genital mutilation/cutting (FGM/C): a systematic review." Reproductive health 2018; **15**(1): 62.
3. United Nations, 2015. 'The Millennium Development Goals Report 2015', pg. 38. http://www.un.org/millenniumgoals/2015_MDG_Report/pdf/MDG%202015
4. UNICEF. Female Genital Mutilation/Cutting: A Global Concern UNICEF, New York, 2016.
5. World Health Organization. Female genital mutilation: Key facts. 2018 [cited 1 May 2022]. https://www.who.int/news-room/fact-sheets/detail/female-genital-mutilation
6. UNFPA-UNICEF Joint Programme on the Elimination of Female Genital Mutilation (2019).

7. Nigeria Demographic and Health Survey (2013). National Population Commission. Federal Republic of Nigeria.
8. Berg RC, Denison E. Health Care for Women International A Tradition in Transition: Factors Perpetuating and Hindering the Continuance of Female Genital Mutilation / Cutting (FGM / C) Summarized in a Systematic Review A Tradition in Transition: Factors Perpetuating Genital. 2013;9332(October 2017).
9. Yaya, S. and B. Ghose (2018). "Female Genital Mutilation in Nigeria: A Persisting Challenge for Women’s Rights." Social Sciences 7(12): 244.
10. Reisel, D. and S. M. Creighton (2015). "Long term health consequences of Female Genital Mutilation (FGM)." Maturitas 80(1): 48-51.
11. Violence against Persons (Prohibition) Act,2015, Laws of the Federation of Nigeria
12. National Population Commission of Nigeria. Nigeria Population and Housing Census 2006. Abuja, Nigeria: National Population Commission of Nigeria.
13. Omole, F.K., Lukman, Y. and Baki, A.I. (2014), “Analysis of market typology and functions in the development of Osun state, Nigeria”, International Journal of Development and Sustainability, Vol. 3 No. 1, pp. 55-69
14. Barret H. Alhassan Y. The REPLACE Community Readiness to End FGM Assessment. Practical Handbook (based on REPLACE Approach www.replacefgm2.eu)2016, Coventry university, UK.
15. McCoy H.V, Malow R., Edwards R.W., Thurland A., Rosenberg R. A Strategy for Improving Community Effectiveness of HIV/AIDS

Intervention Design: The Community Readiness Model in the Caribbean, Substance Use & Misuse,2007; 42:10, 1579-1592, DOI: 10.1080/10826080701212535

16. Borrayo, E. A. (2007). "Using a community readiness model to help overcome breast health disparities among US Latinas." Substance use & misuse **42**(4): 603-619.
17. Duma, S. Women's journey of recovery from sexual assault trauma: a grounded theory.2006.
18. Van Teijlingen, E R, Hundley V. "The importance of pilot studies."(2001).
19. Braun V, Clarke V. Thematic analysis. in H. Cooper, P. M. Camic, D. L. Long, A. T. Panter, D. Rindskopf, & K. J. Sher (Eds.), *APA handbook of research methods in psychology, 2012;Vol. 2. Research designs: Quantitative, qualitative, neuropsychological, and biological* (pp. 57–71). American Psychological Association. https://doi.org/10.1037/13620-004
20. Louis Cohen L, Manion L, Morrison K. Action research. Research methods in education, Routledge 2013:368-385.
21. Polit, D. and C. Beck. "Essentials of nursing research." Ethics2012; **23**(2).
22. Fawcett J, Garity J. "Chapter 1: Research and evidence-based nursing practice." Evaluating research for evidence-based nursing: (2009); 3-20.
23. Dunn F. Is It Possible to End Female Circumcision in Africa? Clin J Obstet Gynecol. 2021;1: 007-013. https://doi.org/10.29328/journal.cjog.1001002
24. Sakeah E, Debpuur C, Aborigo RA, Oduro AR, Sakeah JK, Moyer CA (2019) Persistent female genital mutilation despite its illegality: Narratives from women and women in northern Ghana. PLoS ONE 14(14):e0214923. https://doi.org/10.1371 journal pone 0214923.Obi A.I., Adesoye O.,

LabiranA. Female Genital Mutilation: Attitude and Practices among Women in Okada Community, Edo State JMBR: A Peer-review Journal of Biomedical Sciences December 2015, Vol. 14 No. 2 pp 138-150

Formatted: English (United States)

24.

25. Andarge, MasreshaYazew. *The difficulties of ending Female Genital Mutilation (FGM): Case of Afar pastoralist communities in Ethiopia*. *Social Policy for Development (SPD) (2014, December 12)*. Retrieved from http://hdl.handle.net/2105/17487

26. Ibrahim I.A, Oyeyemi A.S, Ekine A.A. Knowledge, Attitude and Practice of Female Genital Mutilation among Doctors and Nurses in Bayelsa state, Niger-Delta of Nigeria. International Journal of Medicine and Biomedical Research Volume 2 Issue 1 January – April 2013

27. UNFPA. 2020. 'Impact of the COVID-19 Pandemic on Family Planning and Ending Gender-Based Violence, Female Genital Mutilation and Child Marriage.' Interim Technical Note.

The political Map of Osun State, Nigeria ~~showing the various towns~~

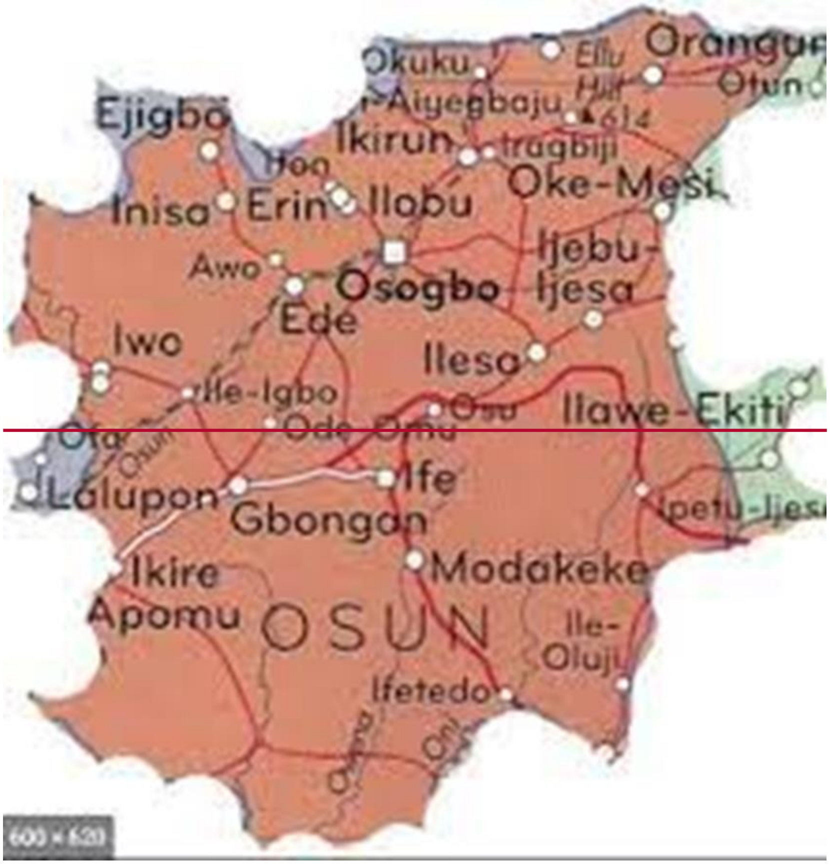

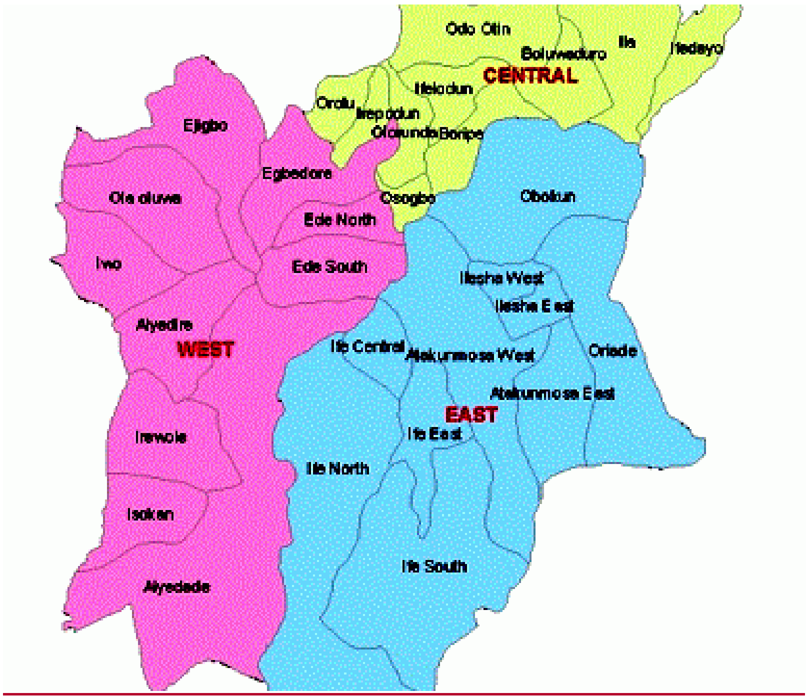

Adapted from https://dawncommission.org

School of Nursing and Public
Health, College of Health
Sciences, Howard Campus,
University of Kwazulu-Natal,
Durban, South Africa.

Dear Editor,

PLos One Journal

REBUTTAL LETTER

Thank you for taking time to go through the manuscript and for the constructive criticism.

Methodology

The Community Readiness Model (CRM) is a research tool that was developed in determining a community's stage of readiness in abandoning a particular harmful behaviour in the community. It is essential because interventions to move communities to the next stage differ for each stage of readiness and unless an intervention is consistent with the community's current stage, such intervention is likely to fail.

The CRM identifies 9 stages of community readiness for prevention. Readiness is assessed by conducting key informant interview to gather information on six community dimensions to guide the community preparedness to move their readiness level forward. The five key dimensions are:

Several studies support the use of the Community Readiness Model to promote acceptability and effectiveness of prevention interventions in other countries. Adopting such a model may be useful for prevention of FGM/C in Osun state. CRM therefore may be useful in addressing previous failures of the current interventions in Osun state, by ensuring that the views of women as a community affected by intergenerational FGM/C are incorporated in the effective framework for prevention of women-to-women intergenerational FGM/C in Osun State.

In the context of the model adapted, the traditional birth attendants are the key informants and community leaders.

Exclusion and inclusion criteria

In the study, the traditional birth attendants were individuals recognized for the conduct of FGM/C in the study area irrespective of the duration of the practice and but those that have never been involved in any prevention of FGM/C programs were excluded to avoid biases.

Ethical issues

It was stated that all ethical principles were adhered to. Also, informed consent forms were issued to those who voluntarily participated in the study including options to choose their convenient date, time, and venue of the interview.

Additional technique of data gathering

To ensure credibility, comprehensive field notes, noting gesture and other non-verbal ques observed during the interviews were recorded and analyzed. This was stated in measure to ensure trustworthiness section.

We sincerely hope that we have been able to throw more light on the issues raised.

Thank you so much

Yours sincerely

Fafowora Rosemary O.

Corresponding author

School of Nursing and Public

Health, College of Health

Sciences, Howard Campus,

University of Kwazulu-Natal,

Durban, South Africa.

Dear Editor,

PLos One Journal

REBUTTAL LETTER

Thank you for taking time to go through the manuscript and for the constructive criticism.

Major comments

1. Results: the text between the quotes have been inserted as reflected in the revised marked copy
2. Discussion: this section has also been reviewed with limitation added.
3. This has been addressed as circumcision is done for both health and religious reasons.
4. The corresponding author is a Nigerian studying in South Africa while the co-author is a South African
5. Back-and-forth translation was done on the interview guide to ensure that there is no loss of meaning as indicated in the marked copy.
6. The time range for the interview was also indicted in the methodology(45-60mins).
7. Authors contribution was also indicated in the methodology. In all, there is an equal contribution to the paper.
8. The map has been included after the references.

Thank you

Rosemary O. Fafowora

Corresponding author

School of Nursing and Public Health

College of Health Sciences

University of KwaZulu Natal

Durban, South Africa

21st June 2023

Dear Editor,

PLos One Journal

REBUTTAL LETTER

Thank you for taking time to go through the manuscript and for the constructive criticism.

1. Reference
   Obi A.I., Adesoye O., LabiranA. Female Genital Mutilation: Attitude and Practices among Women in Okada Community, Edo State JMBR: A Peer-review Journal of Biomedical Sciences December 2015, Vol. 14 No. 2 pp 138-150
   The above reference was retracted and substituted wit h a more recent reference.

2. Discussion
   Findings have been further reviewed according to other literatures and summary of key findings.
   The cited references were also briefly described as requested.
3. Strength and limitation of the key findings of the study has also been reviewed as shown in the manuscript.
4. A new map has been added to the manuscript indicating the three senatorial districts that constitute osun state where the study was carried out.
5. The contributions of each author have been highlighted as requested.

We sincerely hope that we have been able to throw more light on the issues raised.

Yours sincerely

Rosemary O, Fafowora
Correspondent Author
