# Supplementary material for: ‘It is what was handed over to us as our heritage and must not be taken away just like that’: Traditional birth attendants’ attitudes towards the elimination of intergenerational female genital mutilation/cutting in Osun State, Nigeria
Source: PLOS Glob Public Health. 2024 May 20;4(5):e0001585. doi: 10.1371/journal.pgph.0001585 (PMC11104658; doi:10.1371/journal.pgph.0001585)
Supplement: Attachment — Submitted filename: Response to reviewers.docx [file pgph.0001585.s002.docx]

School of Nursing and Public

Health, College of Health

Sciences, Howard Campus,

University of Kwazulu-Natal,

Durban, South Africa.

Dear Editor,

PLos One Journal

**REBUTTAL LETTER**

Thank you for taking time to go through the manuscript and for the constructive criticism.

**Methodology**

The Community Readiness Model (CRM) is a research tool that was developed in determining a community’s stage of readiness in abandoning a particular harmful behaviour in the community. It is essential because interventions to move communities to the next stage differ for each stage of readiness and unless an intervention is consistent with the community’s current stage, such intervention is likely to fail.

The CRM identifies 9 stages of community readiness for prevention. Readiness is assessed by conducting key informant interview to gather information on six community dimensions to guide the community preparedness to move their readiness level forward. The five key dimensions are:

Several studies support the use of the Community Readiness Model to promote acceptability and effectiveness of prevention interventions in other countries. Adopting such a model may be useful for prevention of FGM/C in Osun state. CRM therefore may be useful in addressing previous failures of the current interventions in Osun state, by ensuring that the views of women as a community affected by intergenerational FGM/C are incorporated in the effective framework for prevention of women-to-women intergenerational FGM/C in Osun State.

In the context of the model adapted, the traditional birth attendants are the key informants and community leaders.

**Exclusion and inclusion criteria**

In the study, the traditional birth attendants were individuals recognized for the conduct of FGM/C in the study area irrespective of the duration of the practice and but those that have never been involved in any prevention of FGM/C programs were excluded to avoid biases.

**Ethical issues**

It was stated that all ethical principles were adhered to. Also, informed consent forms were issued to those who voluntarily participated in the study including options to choose their convenient date, time, and venue of the interview.

**Additional technique of data gathering**

To ensure credibility, comprehensive field notes, noting gesture and other non-verbal ques observed during the interviews were recorded and analyzed. This was stated in measure to ensure trustworthiness section.

We sincerely hope that we have been able to throw more light on the issues raised.

Thank you so much

Yours sincerely

Fafowora Rosemary O.

Corresponding author
