# Supplementary material for: ‘It is what was handed over to us as our heritage and must not be taken away just like that’: Traditional birth attendants’ attitudes towards the elimination of intergenerational female genital mutilation/cutting in Osun State, Nigeria
Source: PLOS Glob Public Health. 2024 May 20;4(5):e0001585. doi: 10.1371/journal.pgph.0001585 (PMC11104658; doi:10.1371/journal.pgph.0001585)
Supplement: Attachment — Submitted filename: Response to the reviewer.docx [file pgph.0001585.s003.docx]

School of Nursing and Public

Health, College of Health

Sciences, Howard Campus,

University of Kwazulu-Natal,

Durban, South Africa.

Dear Editor,

PLos One Journal

**REBUTTAL LETTER**

Thank you for taking time to go through the manuscript and for the constructive criticism.

Major comments

1. Results: the text between the quotes have been inserted as reflected in the revised marked copy
2. Discussion: this section has also been reviewed with limitation added.
3. This has been addressed as circumcision is done for both health and religious reasons.
4. The corresponding author is a Nigerian studying in South Africa while the co-author is a South African
5. Back-and-forth translation was done on the interview guide to ensure that there is no loss of meaning as indicated in the marked copy.
6. The time range for the interview was also indicted in the methodology(45-60mins).
7. Authors contribution was also indicated in the methodology. In all, there is an equal contribution to the paper.
8. The map has been included after the references.

Thank you

Rosemary O. Fafowora

Corresponding author
