# Supplementary material for: ‘It is what was handed over to us as our heritage and must not be taken away just like that’: Traditional birth attendants’ attitudes towards the elimination of intergenerational female genital mutilation/cutting in Osun State, Nigeria
Source: PLOS Glob Public Health. 2024 May 20;4(5):e0001585. doi: 10.1371/journal.pgph.0001585 (PMC11104658; doi:10.1371/journal.pgph.0001585)
Supplement: Attachment — Submitted filename: Rebuttal Letter.docx [file pgph.0001585.s004.docx]

School of Nursing and Public Health

College of Health Sciences

University of KwaZulu Natal

Durban, South Africa

21^st^ June 2023

Dear Editor,

PLos One Journal

**REBUTTAL LETTER**

Thank you for taking time to go through the manuscript and for the constructive criticism.

1. Reference

Obi A.I., Adesoye O., LabiranA. Female Genital Mutilation: Attitude and Practices among Women in Okada Community, Edo State JMBR: A Peer-review Journal of Biomedical Sciences December 2015, Vol. 14 No. 2 pp 138-150

The above reference was retracted and substituted wit h a more recent reference.

2. Discussion

Findings have been further reviewed according to other literatures and summary of key findings.

The cited references were also briefly described as requested.

3. Strength and limitation of the key findings of the study has also been reviewed as shown in the manuscript.
4. A new map has been added to the manuscript indicating the three senatorial districts that constitute osun state where the study was carried out.
5. The contributions of each author have been highlighted as requested.

We sincerely hope that we have been able to throw more light on the issues raised.

Yours sincerely

Rosemary O, Fafowora

Correspondent Author
