# Supplementary material for: ‘It is what was handed over to us as our heritage and must not be taken away just like that’: Traditional birth attendants’ attitudes towards the elimination of intergenerational female genital mutilation/cutting in Osun State, Nigeria
Source: PLOS Glob Public Health. 2024 May 20;4(5):e0001585. doi: 10.1371/journal.pgph.0001585 (PMC11104658; doi:10.1371/journal.pgph.0001585)
Supplement: Attachment — Submitted filename: 1.docx 2.docx [file pgph.0001585.s006.docx]

1. World Health Organization. Female Genital Mutilation. Fact sheet. 2020 Retrieved February 20, 2022, from http://www.who.int/mediacentre/factsheets/fs241/en/
2. Hassan H., Abd-ELhakam F., Ebrahim R., Mohammed M. Study Females' Attitude toward Female Genital Mutilation. Journal of Gynecology and Women’s Health, 2022, 24(3): 1-9 DOI: 10.19080/JGWH.2022.24.556137
3. Mohammed M., Abd-ELhakam F., Hassan H., Ebrahim R. Study Females' Intention to practice Female Genital Mutilation for their Daughters at Beni-Suef. Public Health Open Access, 2022; 6(2):1-8: DOI: 10.23880/phoa-16000224
4. Ebrahim R., Abd-ELhakam F., Hassan H., Mohammed M. Relationship between Females' Knowledge and Attitude and Intention of Female Genital Mutilation. [Research in Psychology and Behavioral Sciences](http://www.sciepub.com/journal/RPBS), 2023; 11(1): 1-6. DOI: 10.12691/rpbs-11-1-1
5. Abd-ELhakam F., Ebrahim R., Mohammed M., Hassan H. Relationship between Socio Demographic Data and Female Genital Mutilation. Archives of Medical Case Reports and Case Study, 2022; 6(5); 1-7. DOI:10.31579/2692- 9392/160
6. Hassan H., Abd-ELhakam F., Ebrahim R., Mohammed M. Call for Change Enhancement Upper Egyptian Females' Knowledge Regarding Effect of Female Genital Mutilation. American Journal of Public Health Research, 2023; 11(1): 5-12. DOI: 10.12691/ajphr-11-1-2
7. Waigwa S, Doos L, Bradbury-Jones, Taylor J. "Effectiveness of health education as an intervention designed to prevent female genital mutilation/cutting (FGM/C): a systematic review." Reproductive health 2018; 15(1): 62.
8. United Nations, 2015. ‘The Millennium Development Goals Report 2015’, pg. 38. http://www.un.org/millenniumgoals/2015_MDG_Report/pdf/MDG%202015
9. Hassan H., Abd-ELhakam F., Zakria M., El-sayed R. Female Genital Mutilation in Northern Upper Egypt: Prospects of Reasons for Performing and Refusing. Nursing and Care Open Access Journal, 2023; 9(1):6‒10. DOI: 10.15406/ncoaj.2023.09.00251
10. Abd- Elhakam F., Ibrahim R., Mohamed M., Hassan H. Prevalence of Female Genital Mutilation at Beni-Suef Governorate, Egypt. Egyptian Journal of Health Care, (EJHC), 2023; 14 (1): 564-577. Doi: [10.21608/EJHC.2023.285610](https://doi.org/10.21608/ejhc.2023.285610)
11. Ebrahim R., Mohammed M., Hassan H., Abd-ELhakam F. Relationship between Personal Characteristics and the Person Performing Female Genital Mutilation. American Journal of Public Health Research, 2023; 11(2): 69-74. DOI: 10.12691/ajphr-11-2-5
12. Abd-ELhakam F., Ebrahim R., Mohammed M., Hassan H. Female Genital Mutilation: Females' Related Knowledge. EC Nursing Health, 2023; 5(4): 29-38.
13. Hassan H., Mohammed M., Abd-ELhakam F., Ebrahim R. Female Genital Mutilation: Study Sexual Issues. Journal of Gynecology and Women’s Health, 2023, 24(4): 1-8. DOI: 10.19080/JGWH.2023.23.556145
14. UNICEF. Female Genital Mutilation/Cutting: A Global Concern UNICEF, New York, 2016.
15. Hassan H., Ebrahim R., Mohammed M., Abd-ELhakam F. Female Genital Mutilation: Impact on Knowledge, Attitude, Sexual Score Domains and Intention of Females in Northern Upper Egypt. Archives of Medical Case Reports and Case Study, 2022; 6(5): 1-8. DOI: <https://doi.org/10.31579/2692-9392/161>
16. Abd-ELhakam F., Ebrahim R., Mohammed M., Hassan H. Female Genital Mutilation: Relationship Association between Upper Egyptian Females' Knowledge and Attitude. International Journal of Clinical Studies & Medical Case Reports, 2023; 29(4): 1-5. DOI: 10.46998/IJCMCR.2023.29.000716
17. World Health Organization. Female genital mutilation: Key facts. 2018 [cited 1 May 2022]. https://www.who.int/news-room/fact-sheets/detail/female-genital-mutilation
18. Hassan H., Abd-ELhakam F., Ebrahim R., Mohammed M. Female Genital Mutilation: Females' Intention in Northern Upper Egypt. Journal of Obstetrics Gynecology and Reproductive Sciences, 2023; 7(1): 1-11. DOI: 10.31579/2578-8965/144
19. Saber N., Abdallah A., Hassan H. Validation of School-Based Health Education on Knowledge and Attitude of Female Students towards Female Genital Mutilation. International Egyptian Journal of Nursing Sciences and Research (IEJNSR), 2023; 4 (1): 85-102.
20. UNFPA-UNICEF Joint Programme on the Elimination of Female Genital Mutilation (2019).
21. Hassan H., Ebrahim R., Mohammed M., Abd-ELhakam F. Prevalence and Determinants of Female Genital Mutilation at Beni-Suef. Journal of Gynecology and Women’s Health, 2022, 24(3): 1-10. DOI: 10.19080/JGWH.2022.23.556138
22. Abd-ELhakam F., Ebrahim R., Mohammed M., Hassan H. Relationship between Women's Intention for Female Genital Mutilation and their Exposure to Complication and Pressure. Journal of Community Medicine and Public Health Reports 2022; 3(10): 1-9. Doi.Org/10.38207/JCMPHR/2022/DEC031002139
23. Hassan H., Abd-ELhakam F., Ebrahim R., Mohammed M. Prevalence of Female Genital Mutilation in Northern Upper Egypt. [American Journal of Nursing Research](http://www.sciepub.com/journal/AJNR), 2023, 11(1): 12-20. DOI: 10.12691/ajnr-11-1-2
24. Nigeria Demographic and Health Survey (2013). National Population Commission. Federal Republic of Nigeria.
25. Berg RC, Denison E. Health Care for Women International A Tradition in Transition: Factors Perpetuating and Hindering the Continuance of Female Genital Mutilation /Cutting (FGM / C) Summarized in a Systematic Review A Tradition in Transition: Factors Perpetuating Genital. 2013; 9332(October 2017).
26. Yaya, S. and B. Ghose (2018). "Female Genital Mutilation in Nigeria: A Persisting Challenge for Women’s Rights." Social Sciences 7(12): 244.
27. Reisel, D. and S. M. Creighton (2015). "Long term health consequences of Female Genital Mutilation (FGM)." Maturitas 80(1): 48-51.
28. Violence against Persons (Prohibition) Act, 2015, Laws of the Federation of Nigeria
29. National Population Commission of Nigeria. Nigeria Population and Housing Census. 2006. Abuja, Nigeria: National Population Commission of Nigeria.
30. Omole, F.K., Lukman, Y. and Baki, A.I. (2014), “Analysis of market typology and functions in the development of Osun state, Nigeria”, International Journal of Development and Sustainability, Vol. 3 No. 1, pp. 55-69
31. Barret H. Alhassan Y. The REPLACE Community Readiness to End FGM Assessment. Practical Handbook (based on REPLACE Approach www.replacefgm2.eu) 2016, Coventry university, UK.
32. McCoy H.V, Malow R., Edwards R.W., Thurland A., Rosenberg R. A Strategy for Improving Community Effectiveness of HIV/AIDS Intervention Design: The Community Readiness Model in the Caribbean, Substance Use & Misuse, 2007; 42:10, 1579-1592, DOI: 10.1080/10826080701212535
33. Borrayo, E. A. (2007). "Using a community readiness model to help overcome breast health disparities among US Latinas." Substance use & misuse 42(4): 603-619.
34. Duma, S. Women's journey of recovery from sexual assault trauma: a grounded theory.2006.
35. Van Teijlingen, E R, Hundley V. "The importance of pilot studies." (2001).
36. Braun V, Clarke V. Thematic analysis. in H. Cooper, P. M. Camic, D. L. Long, A. T. Panter, D. Rindskopf, & K. J. Sher (Eds.), APA handbook of research methods in psychology, 2012;Vol. 2. Research designs: Quantitative, qualitative, neuropsychological, and biological (pp. 57–71). American Psychological Association. https://doi.org/10.1037/13620-004
37. Louis Cohen L, Manion L, Morrison K. Action research. Research methods in education, Routledge 2013: 368-385.
38. Polit, D. and C. Beck. "471 Essentials of nursing research." Ethics 2012; 23(2).
39. Fawcett J, Garity J. "Chapter 1: Research and evidence-based nursing practice." Evaluating research for evidence-based nursing: (2009); 3-20.
40. Dunn F. Is It Possible to End Female Circumcision in Africa? Clin J Obstet Gynecol. 2021; 1: 007-013. https://doi.org/10.29328/journal.cjog.1001002
41. Obi A.I., Adesoye O., Labiran A. Female Genital Mutilation: Attitude and Practices among Women in Okada Community, Edo State JMBR: A Peer-review Journal of Biomedical Sciences December 2015, Vol. 14 No. 2 pp 138-150
42. Andarge, Masresha Yazew. The difficulties of ending Female Genital Mutilation (FGM): Case of Afar pastoralist communities in Ethiopia. Social Policy for Development (SPD) (2014, December 12). Retrieved from http://hdl.handle.net/2105/17487
43. Ibrahim I.A, Oyeyemi A.S, Ekine A.A. Knowledge, Attitude and Practice of Female Genital Mutilation among Doctors and Nurses in Bayelsa state, Niger-Delta of Nigeria. International Journal of Medicine and Biomedical Research Volume 2 Issue January – April 2013
44. UNFPA. 2020. ‘Impact of the COVID-19 Pandemic on Family Planning and Ending Gender-Based Violence, Female Genital Mutilation and Child Marriage.’ Interim Technical Note.
