# Supplementary material for: ‘It is what was handed over to us as our heritage and must not be taken away just like that’: Traditional birth attendants’ attitudes towards the elimination of intergenerational female genital mutilation/cutting in Osun State, Nigeria
Source: PLOS Glob Public Health. 2024 May 20;4(5):e0001585. doi: 10.1371/journal.pgph.0001585 (PMC11104658; doi:10.1371/journal.pgph.0001585)
Supplement: Attachment — Submitted filename: RESPONSE TO REVIEWER.docx [file pgph.0001585.s007.docx]

School of Nursing and Public Health

College of Health Sciences

University of KwaZulu Natal

Durban, South Africa

21^st^ September 2023

Dear Editor,

PLos One Journal

**REBUTTAL LETTER**

Thank you for taking time to go through the manuscript and for the constructive criticism.

1. The authors only adapted the REPLACE community readiness model as it was stated in the manuscript. However, with the themes that emerged, the authors have been able to identify the corresponding stage and same documented according to the model.
2. The statement that looks like the result of the study has been removed as advised.
3. The authors stated that we adapted the REPLACE community readiness model. In the model the community leaders are described as the key informants and the key informants are the TBAs. The references are there.

Secondly, the nurses were not our target population. They were only used as gate keepers to have access to the TBAs living in the community where they are practicing. They did not refuse because it is also part of their activities after the researcher explained the essence and neutrality of the study. Further explanation has been included in the sampling criteria.

Like it was mentioned in the methodology part of the research, the TBAs were purposely selected. These group of people are the ones that are traditionally performing the female circumcision and were willing to lend their voice.

4. Positionality of the investigator: this has been discussed under strength and limitation.
5. The TBAs that were interviewed are the traditional people from different towns and it is through the interview that was conducted that the issue of lineage came up.
6. References

**The authors observed that most of our references were deleted and replaced with some other references. The reviewer has suggested the addition of these references throughout the manuscript but did not indicate its value as additional references.**

**We, therefore, want to state that we will not be able to add those references because we did not use or read them to know how relevant they are as the references that we already have are relevant to this study.**

7. The last paragraph of the discussion has been looked into as suggested.

We sincerely hope that we have been able to throw more light on the issues raised.

Yours sincerely

Rosemary O, Fafowora

Correspondent Author
